# Supplementary material for: iPhemap: an atlas of phenotype to genotype relationships of human iPSC models of neurological diseases
Source: EMBO Mol Med. 2017 Oct 19;9(12):1742–62. doi: 10.15252/emmm.201708191 (PMC5731211; doi:10.15252/emmm.201708191)
Supplement: Supplementary file 1 — Appendix [file EMMM-9-1742-s001.pdf]

Appendix

iPhemap: An atlas of phenotype to genotype relationships of human iPSC models of neurological diseases

Ethan W. Hollingsworth[1,2], Jacob E. Vaughn[1,2], Josh C. Orack[1,2], Chelsea Skinner[1,2], Jamil Khouri[1,2], Sofia B. Lizarraga[3], Mark E. Hester[4], Fumihiro Watanabe[1], Kenneth S. Kosik[5] and Jaime Imitola[1,2,6]

Table of Contents

1. **Appendix Figures S1 to S9**
2. **Appendix Tables S1 to S12**

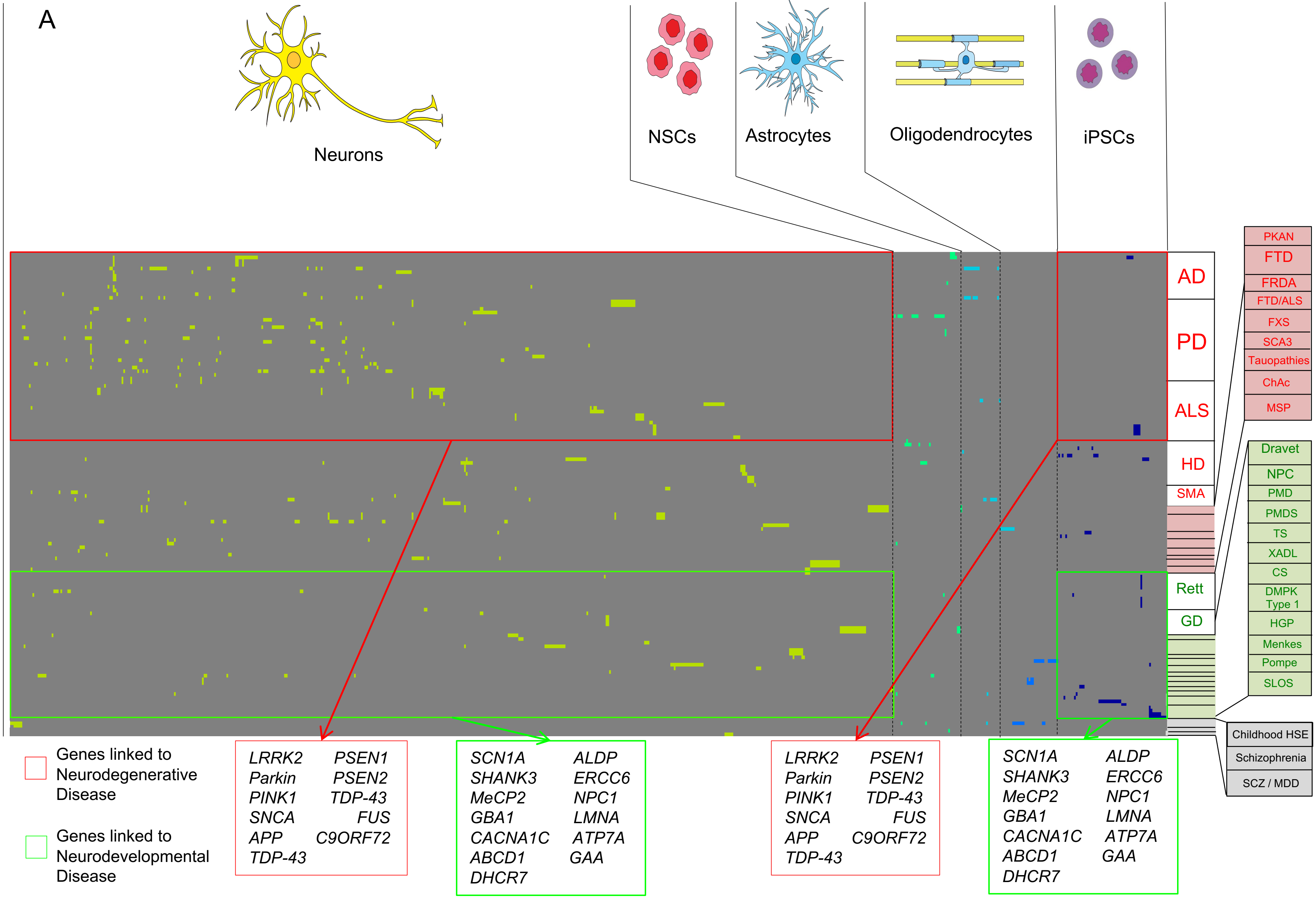

Appendix Figure S1. Heatmap of the relationship between phenotypes and developmental stages *in vitro*. Colored boxes denote the relationship of similar genes at stages of differentiation. Phenotypes are in the X-axis and disease abbreviations are on the Y-axis, grouped by their disease category: neurodegenerative (red) neurodevelopmental (green), and viral-induced and psychiatric (black). The majority of phenotypes reported in neurodegenerative diseases were in mature cell types, while phenotypic abnormalities were observed through all cell types in genes linked to neurodevelopmental disorders. Quantifications can be found in **Figure 4** and a full table of phenotypes, organized from left to right, in **Appendix Table S4**.

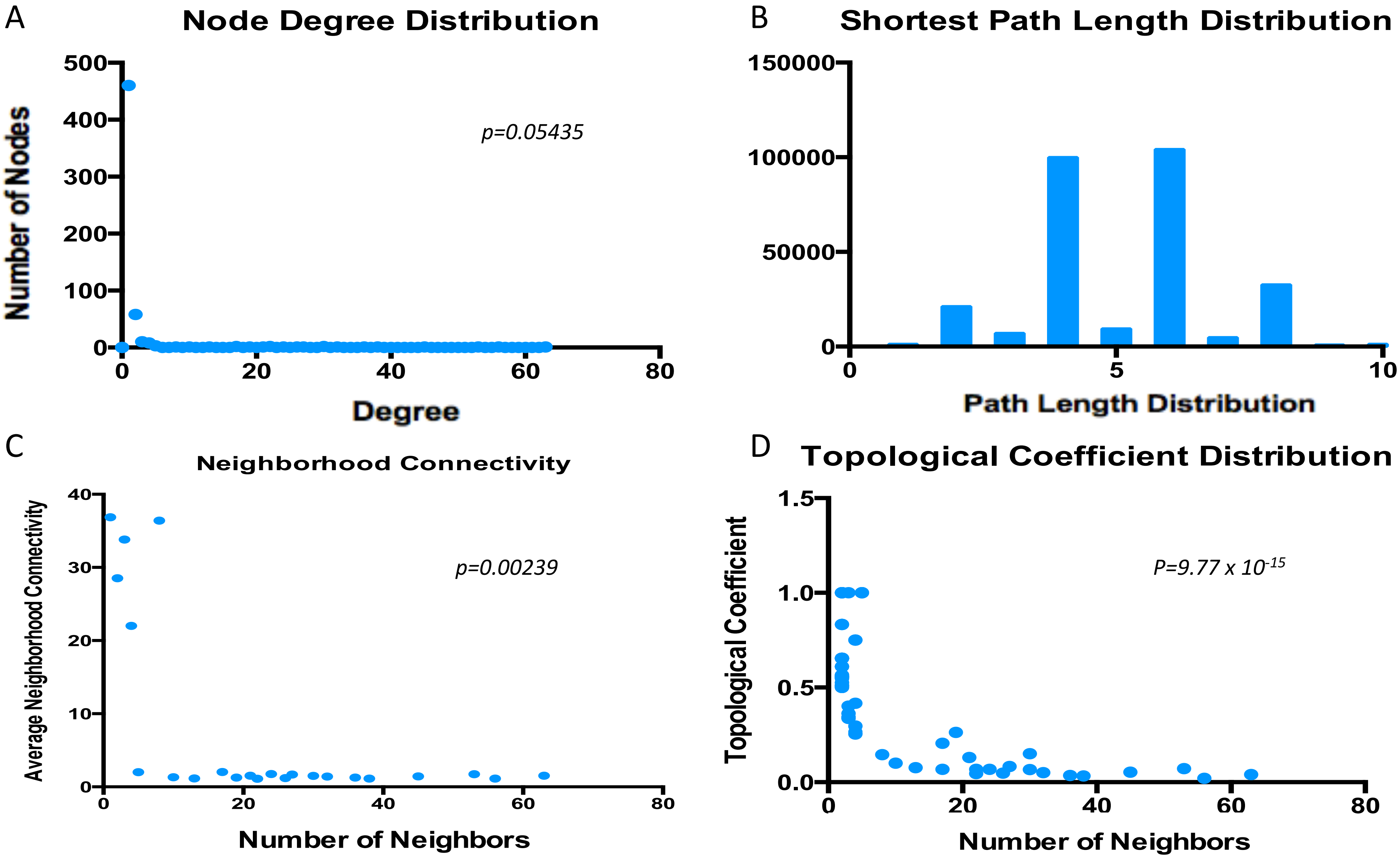

Appendix Figure S2. Statistical pathway analysis of overlapping phenogenetic network. A) Node degree distribution plot of overlapping network. Fitting these data with a power-law distribution returned the fitted line equation, $y=53.358x^{-1.163}$, with the following statistical parameters: R^2 = 0.717 and r= 0.922, which suggests that this network is in accord with a scale-free network (*n*=64, F(1,62)=3.85). **B)** Shortest path length distribution plot illustrating the smallest number of phenotypes between genes. **C)** Neighborhood connectivity (*n*=23, F(1,21)=11.91) and **D)** Topological coefficient distribution (*n*=100, F(1,98)=70.71) are both statistically significant for a power-law distribution, which demonstrates that a majority of phenotypes are distinct to a single gene within the network.

2017 Patient-Derived iPSC Phenogenetic Map

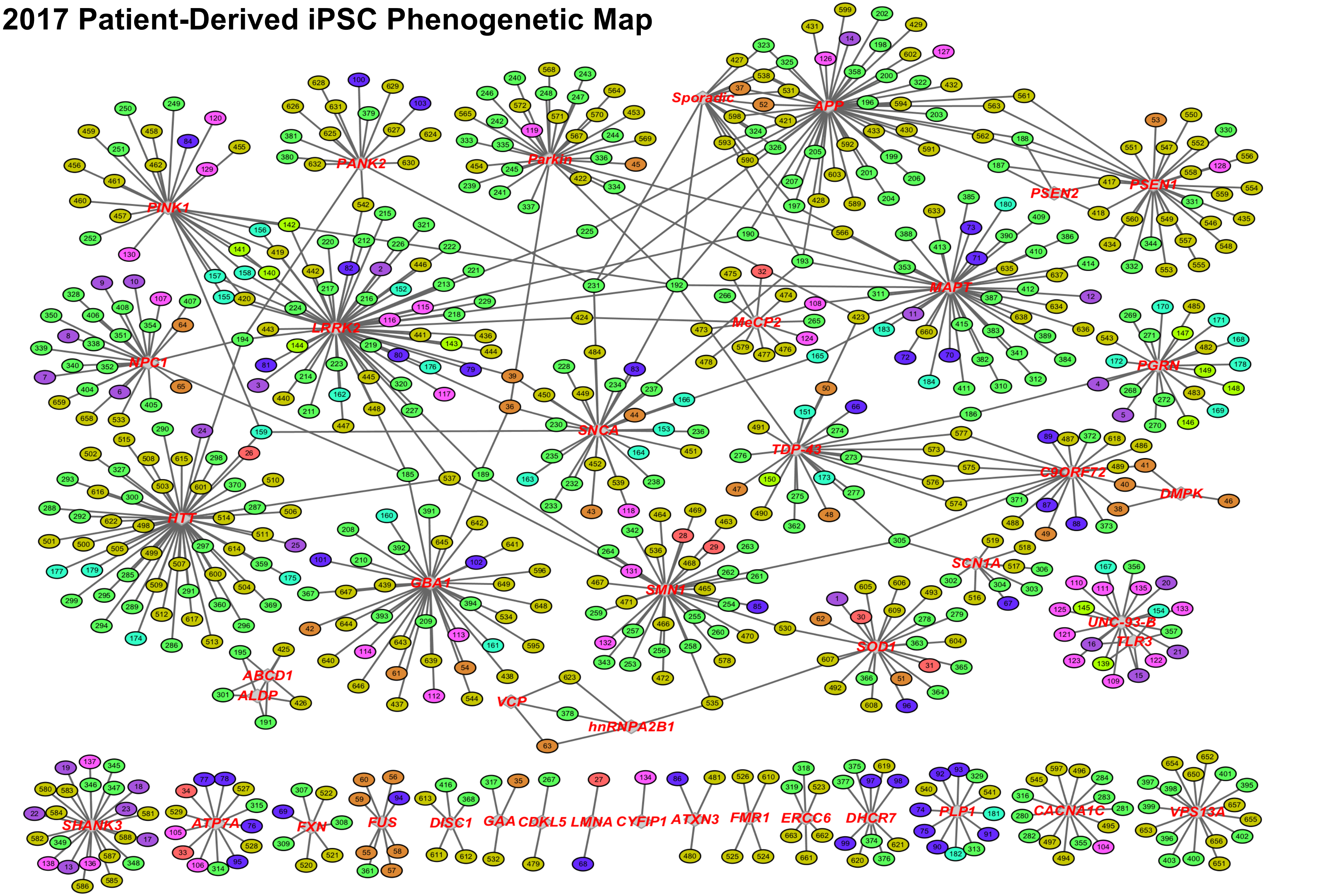

Appendix Figure S3. Extended patient-derived iPSC phenogenetic map version 2017, including non-overlapping genes. Phenotypes are colored by taxonomy described in **Figure EV2** and identifying numbers of each phenotype are listed in **Appendix Table S10**. This extended network view was generated through the same method of analysis as **Figure 5.**

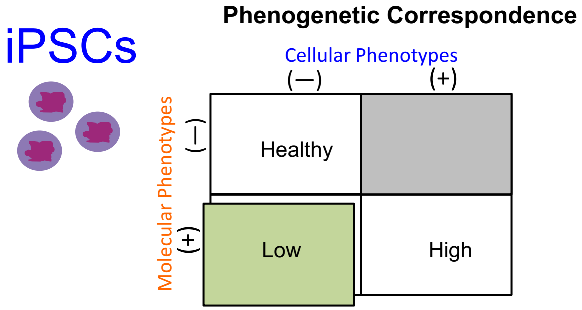

Appendix Figure S4A-D. Phenogenetic correspondence of iPSCs. The treemap view illustrates the correspondence of cellular to molecular phenotypes from iPSCs. Low phenogenetic correspondence, characterized by the absence of cellular phenotypes, was noted for the downregulated and upregulated functional annotations of iPSCs with mutations in **A-D)** *LRRK2* and *SNCA*.

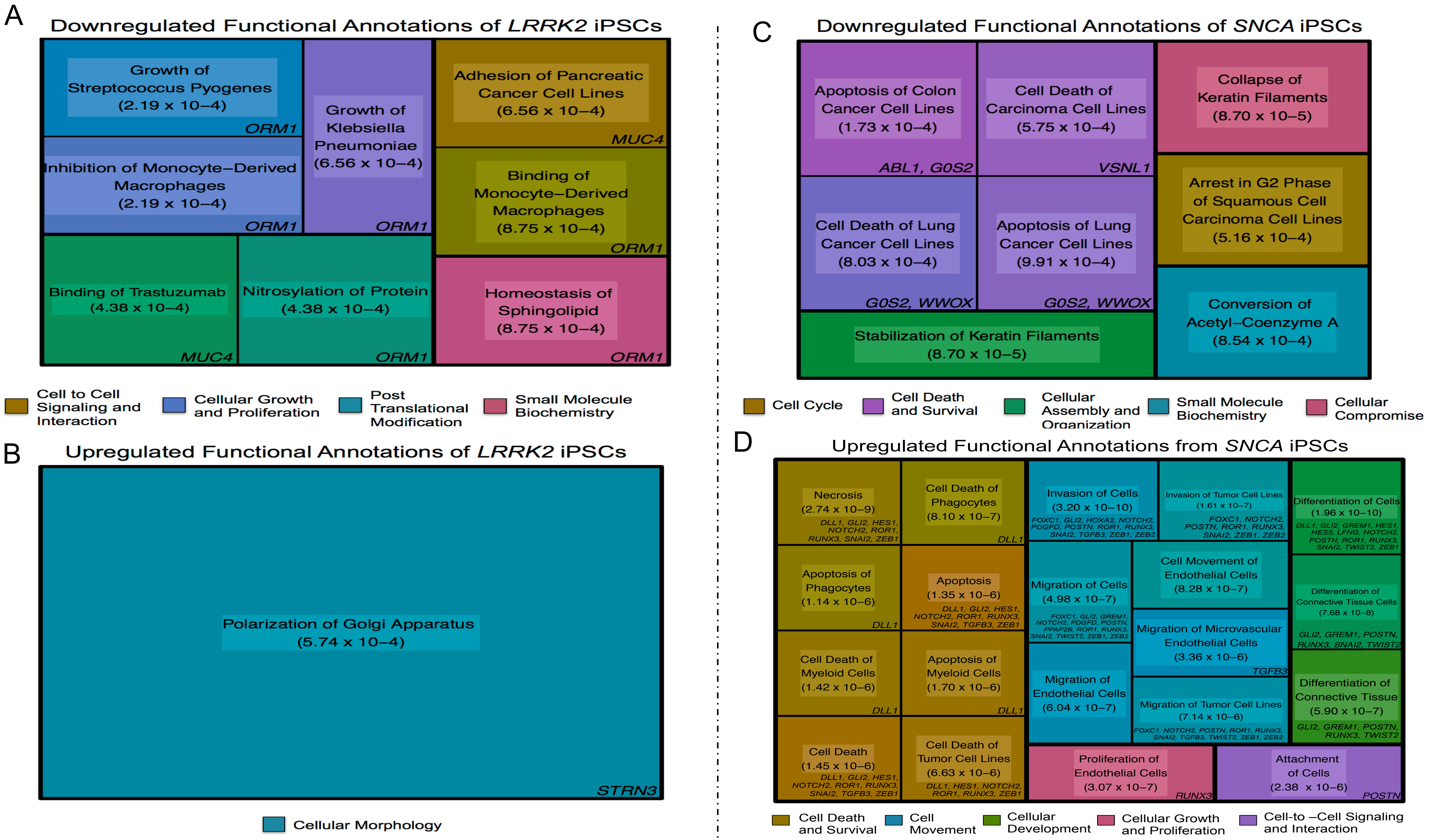

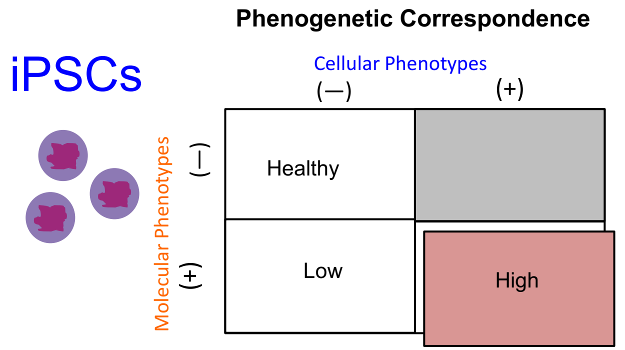

Appendix Figure S4E-J. Phenogenetic correlation of iPSCs. The treemap view illustrates the correlation of cellular to molecular phenotypes from iPSCs. **E-F**) Downregulated and upregulated functional annotations of iPSCs with mutations in *FXN*. Altered expression of *DLK1*, *GDF15*, and *IGFBP7*, which are critical to motor neuron function[9-11], was found in these cells. iPSCs with mutant **G-H**) *ERCC6* and **I-J**) *HTT* show abnormalities in expression of genes related to migration, like *CXCR4* (middle), and genes involved in cytokinesis and cell viability (right), respectively. These molecular changes are indicative of high phenogenetic correspondence.

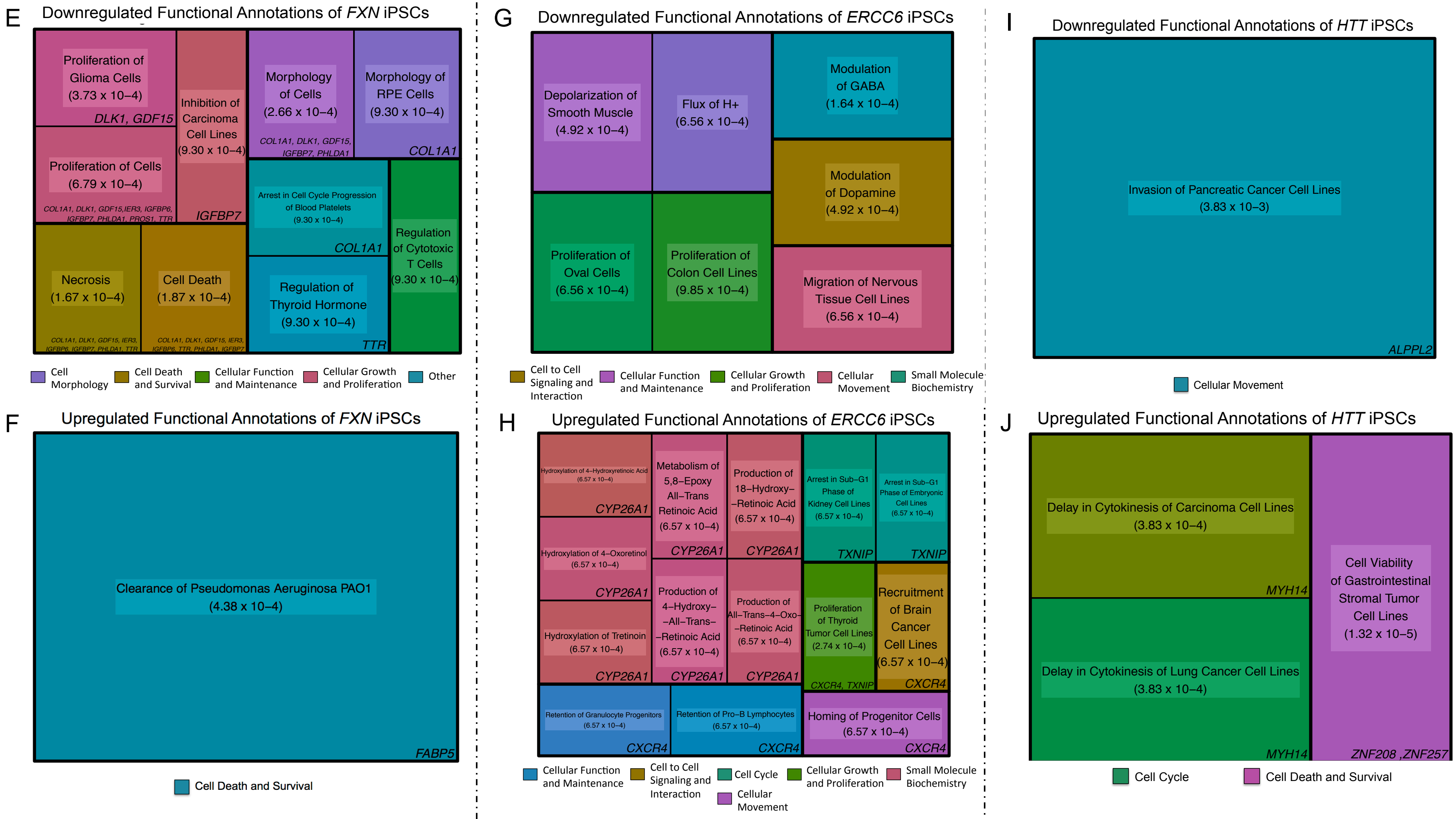

iPSCs

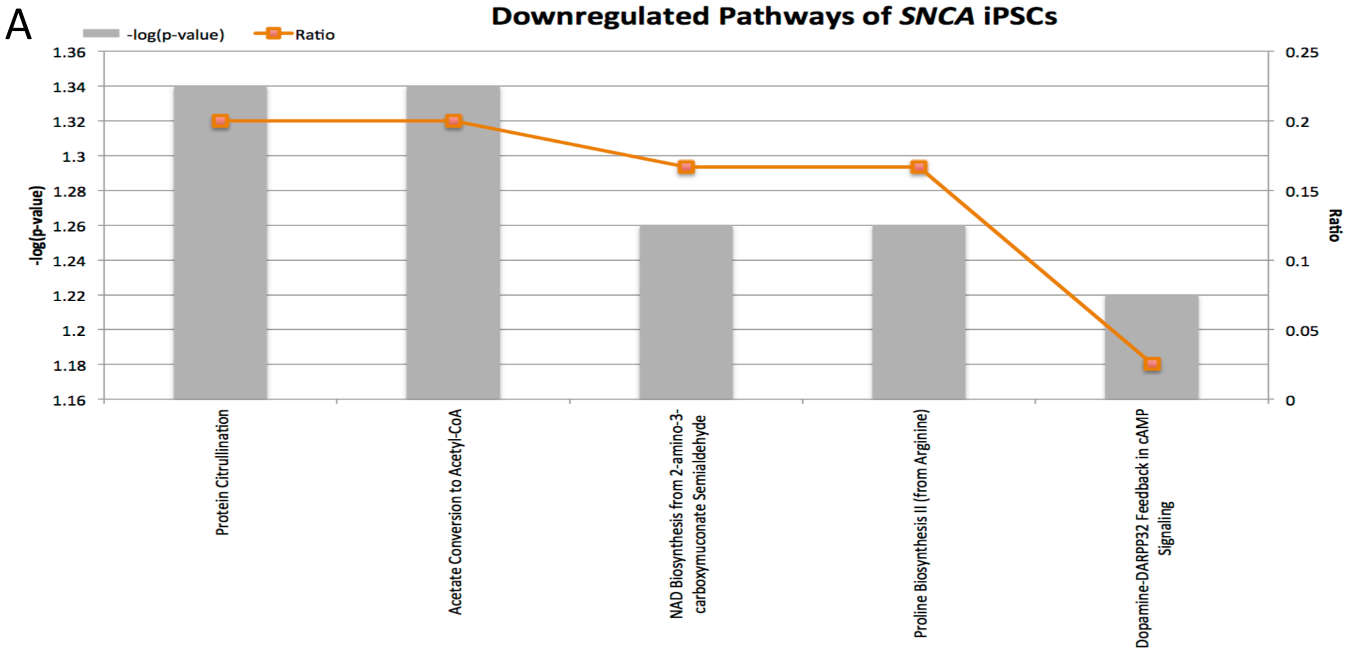

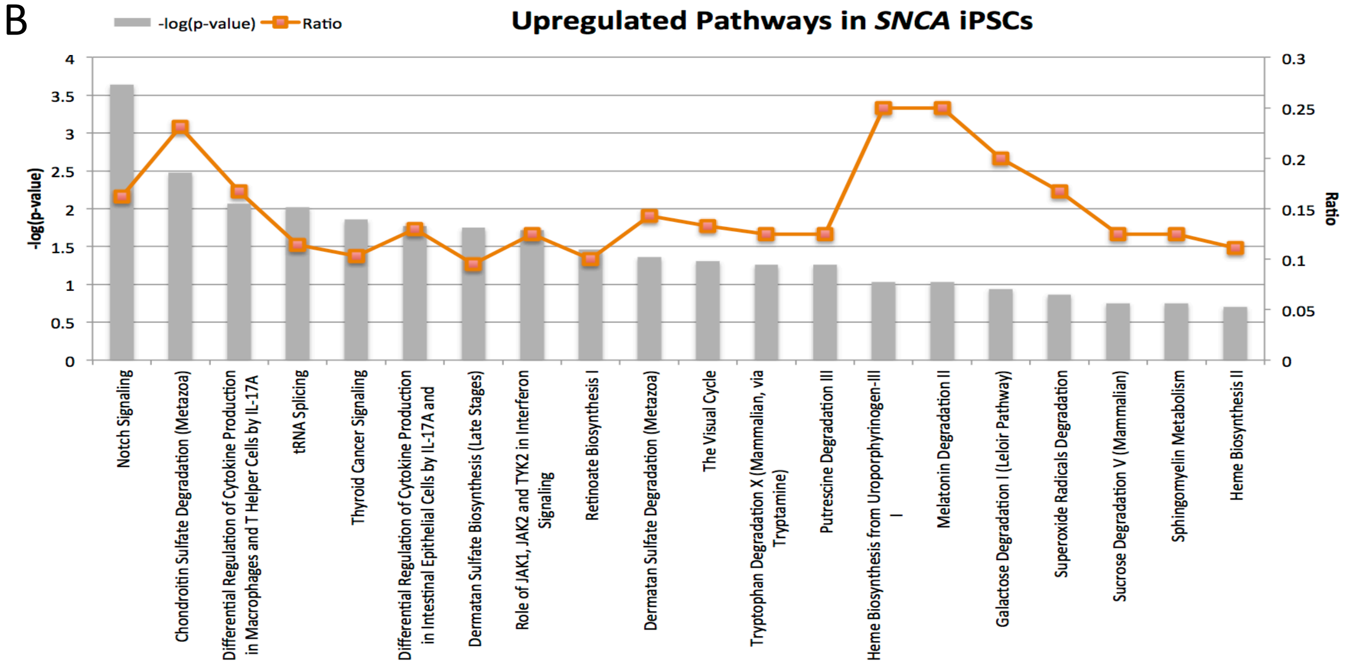

Appendix Figure S5A, B. Extended molecular profile data of iPSCs containing *SNCA* mutation with **A**) downregulation of molecular pathways, such as dopamine signaling, and **B**) upregulation of metabolic pathways and Notch signaling, as seen in the treemaps of **Appendix Figure S4C, D.**

iPSCs

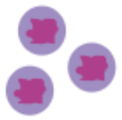

C

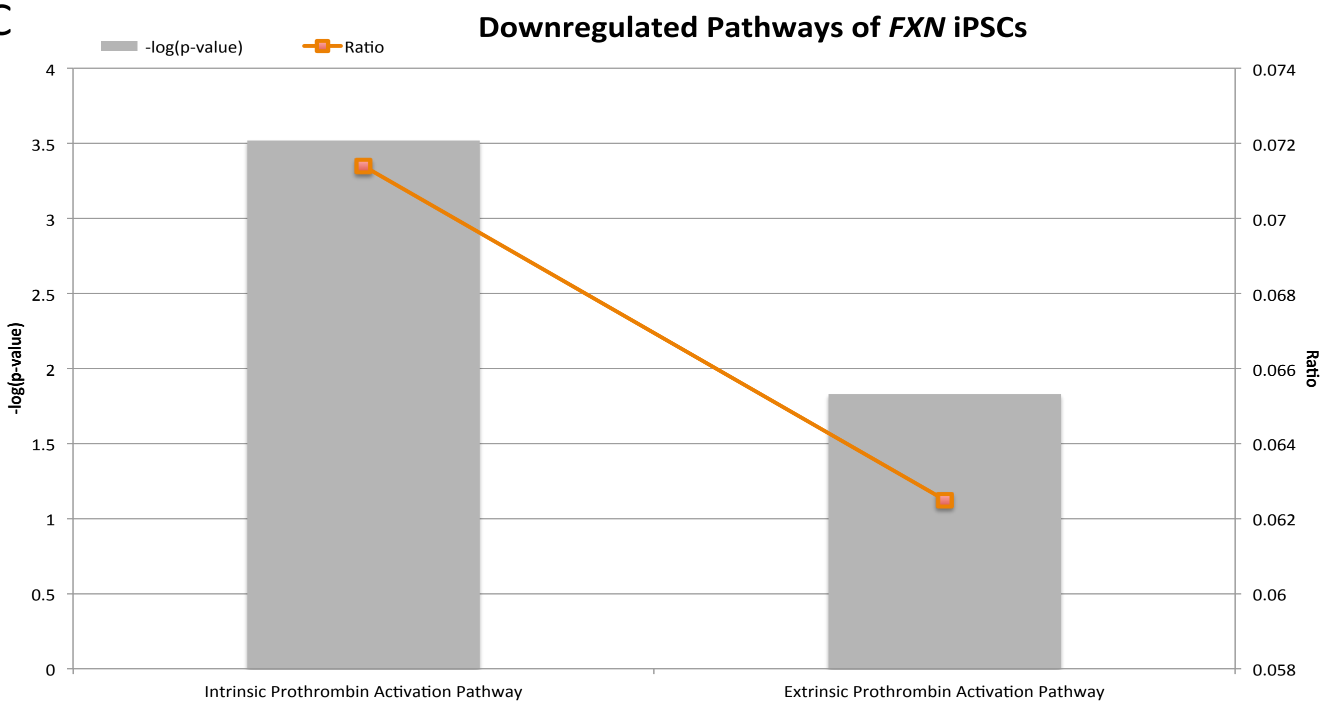

D

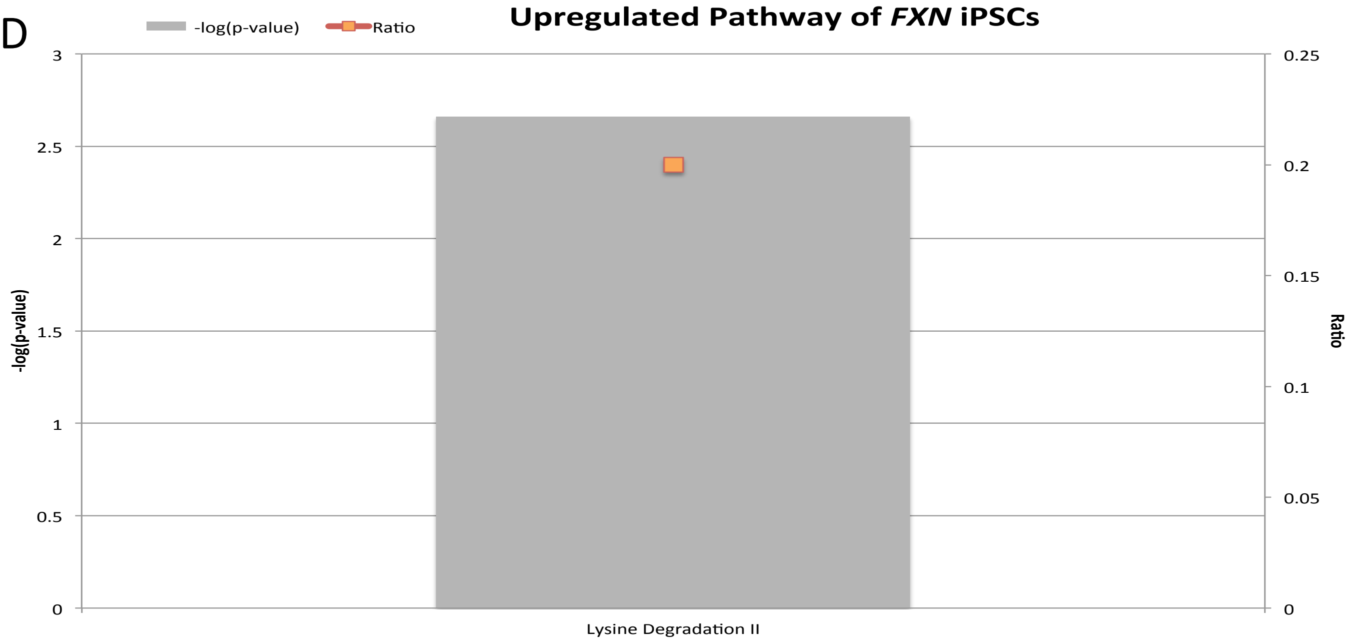

Appendix Figure S5C, D. Extended molecular profile data of iPSCs containing *FXN* mutations with **C**) downregulation of thrombin activation signaling pathways, and **D**) upregulation of amino acid catabolic pathway, as seen in **Appendix Figure S4E, F.**

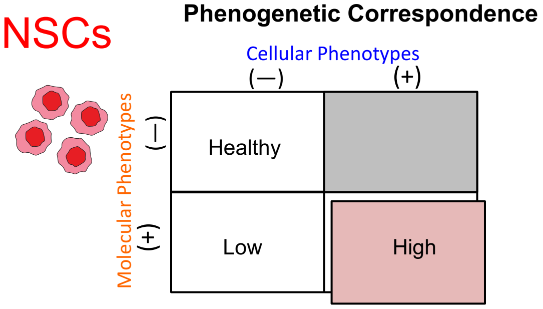

Appendix Figure S6. Phenogenetic correlation of NSCs. In this treemap view, high phenogenetic correlation was established for NSCs with mutations in *LRRK2*. **A**) Upregulated functional annotations of these cells show increased expression of genes associated with nitric oxide process and apoptosis. **B**) Downregulated functional annotations indicate dysregulation of neural progenitor processes, with a decrease in *NR2E1*, *PLP1*, and *PAX6*, which are all involved in neurogenesis and NSC self-renewal[12-14].

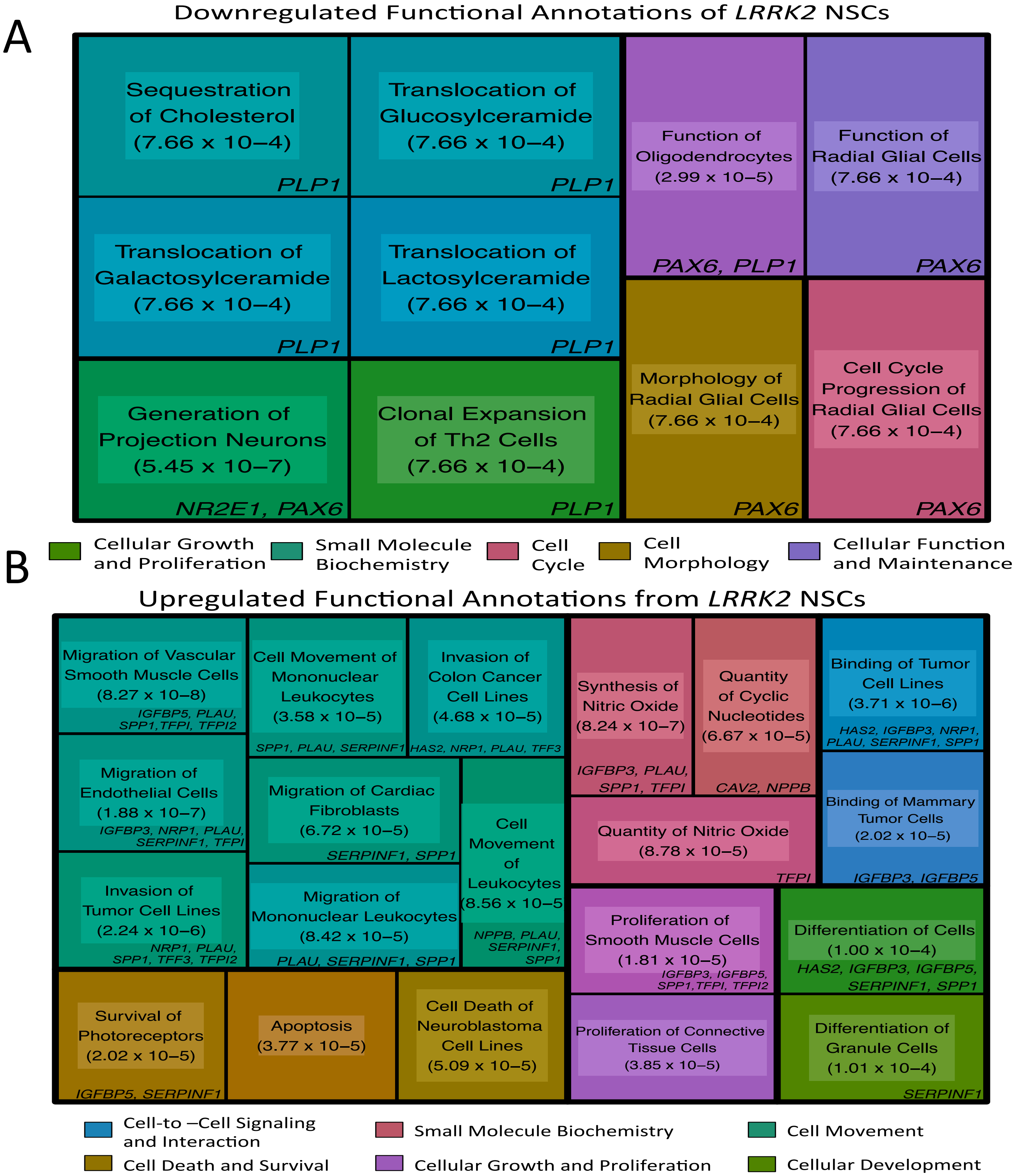

NSCs

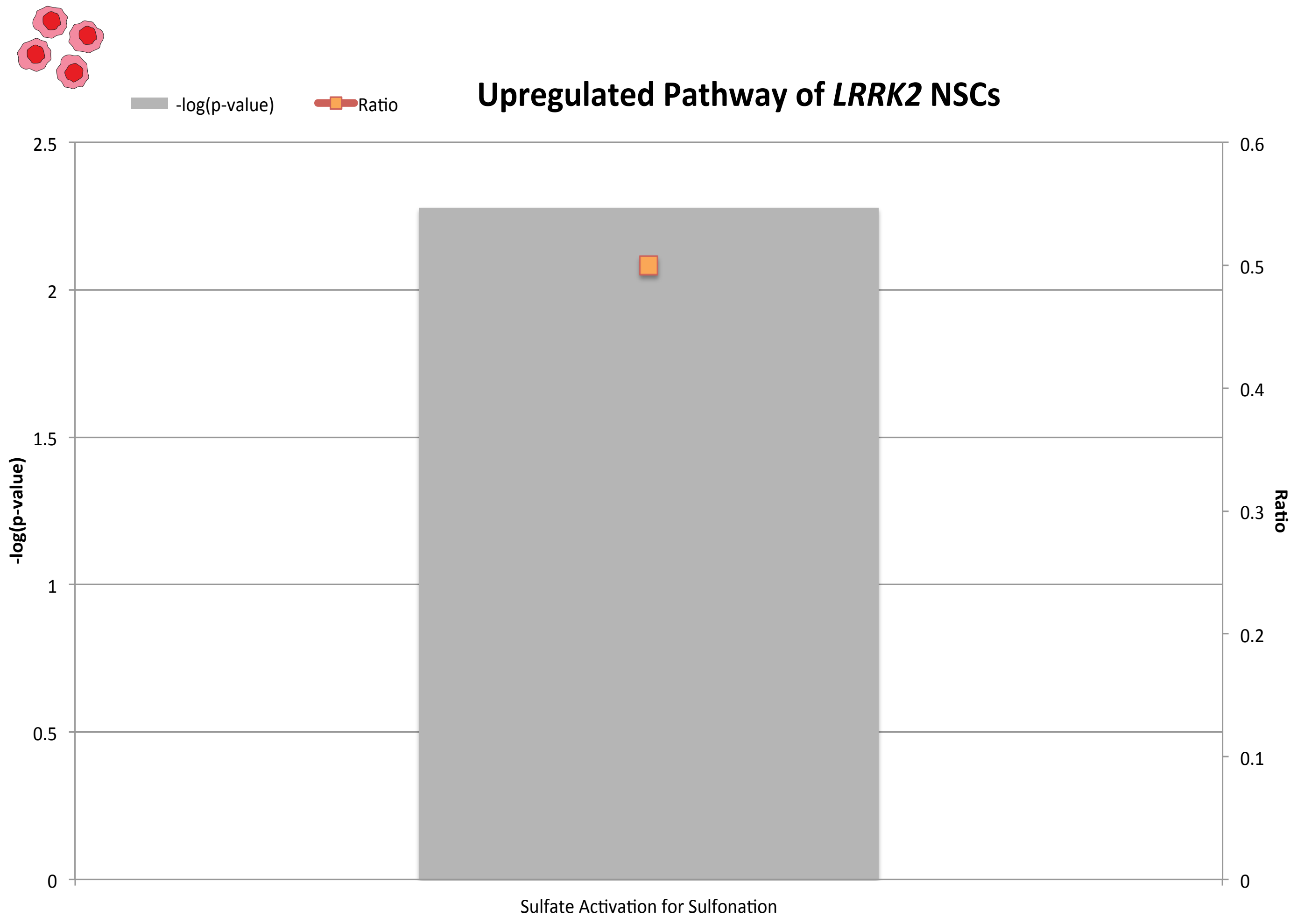

Appendix Figure S7. Extended molecular profile data of NSCs containing mutant *LRRK2* with upregulation of a post-translational modification pathway, induced by transcriptional dysregulation as featured in treemaps of **Appendix Figure S6**.

Neurons

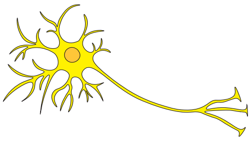

A

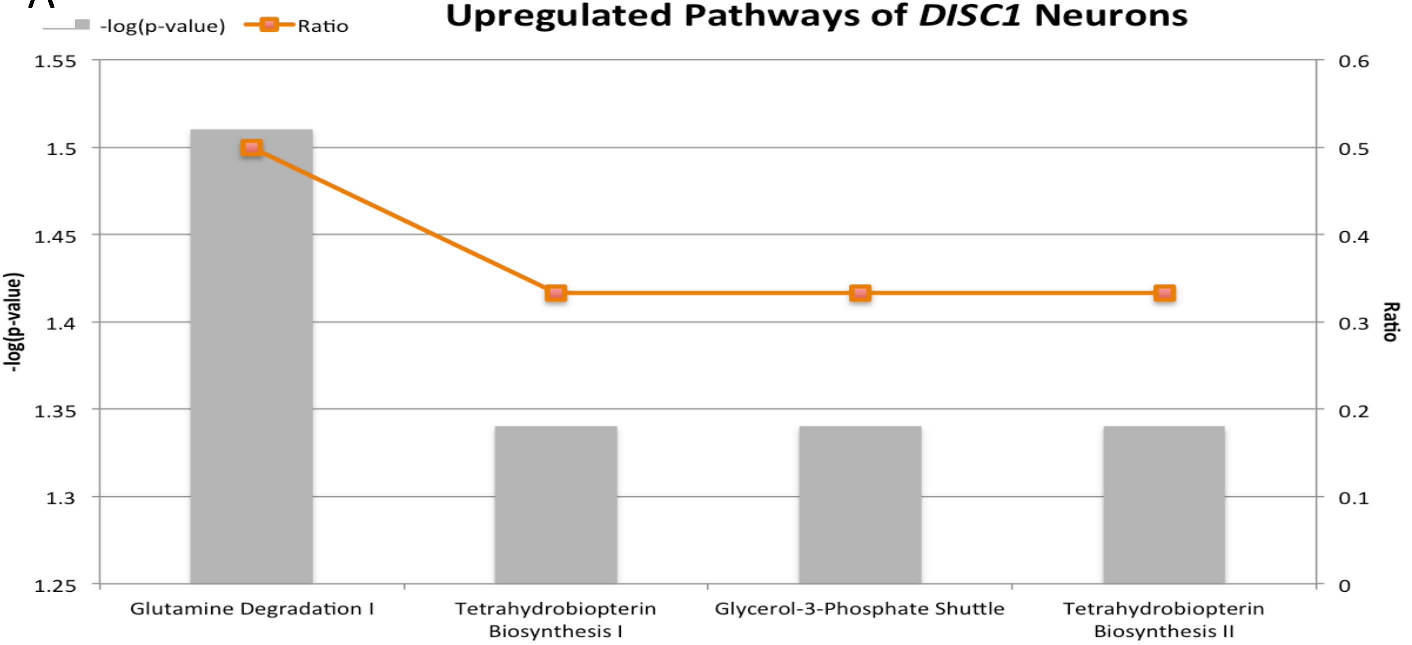

B

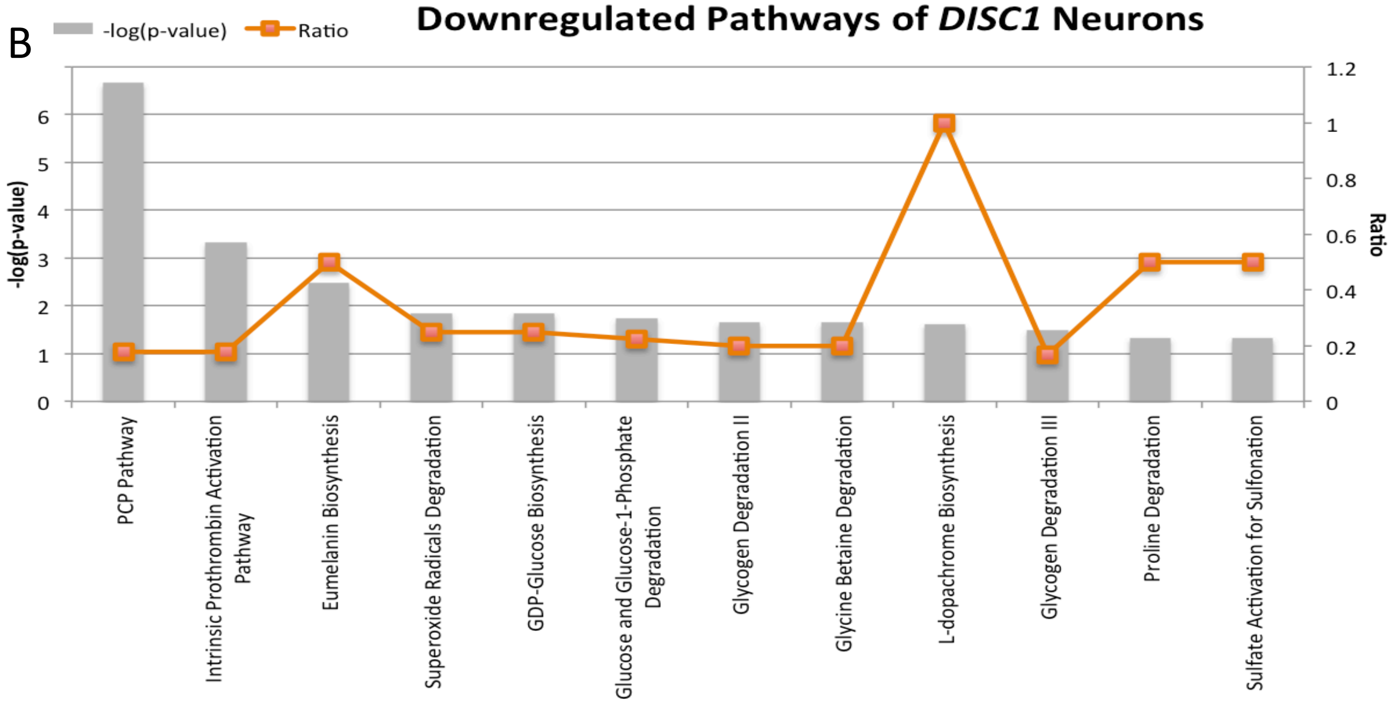

Appendix Figure S8. Extended molecular profile data of neurons with mutations in *DISC1.* **A**) Upregulation of amino acid cofactor pathways and **B**) downregulation of superoxide radical and glucose metabolism pathways, induced by transcriptional dysregulation as seen in treemaps in **Figure 6D, E**.

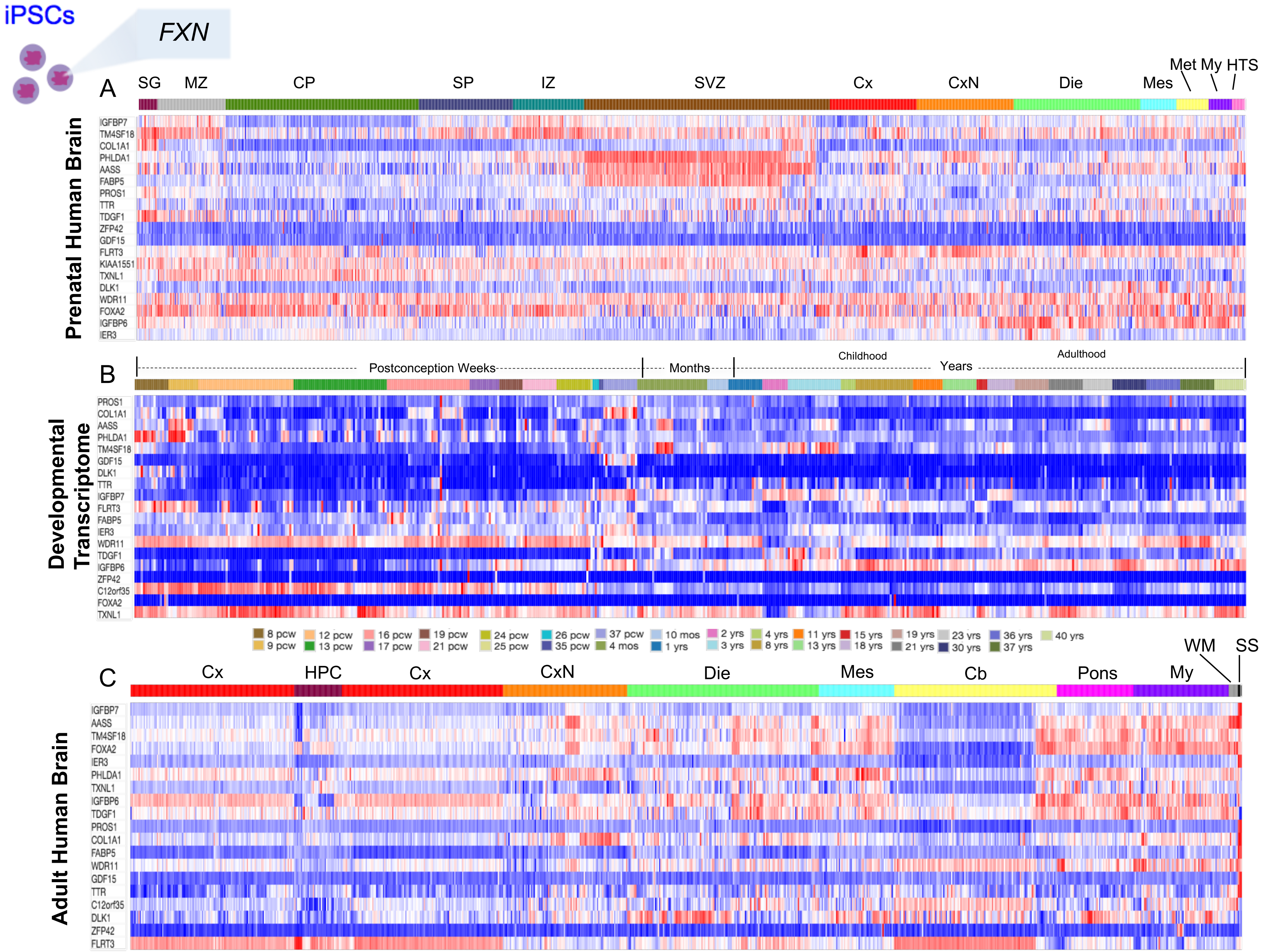

iPSCs
FXN
A
SG
MZ
CP
SP
IZ
SVZ
Cx
CxN
Die
Mes
Met
My
HTS
Prenatal Human Brain
IGFBP7
TM4SF18
COL1A1
PHLDA1
AASS
FABP5
PROS1
TTR
TDGF1
ZFP42
GDF15
FLRT3
KIAA1551
TXNL1
DLK1
WDR11
FOXA2
IGFBP6
IER3
B
Postconception Weeks
Months
Childhood
Years
Adulthood
Developmental Transcriptome
PROS1
COL1A1
AASS
PHLDA1
TM4SF18
GDF15
DLK1
TTR
IGFBP7
FLRT3
FABP5
IER3
WDR11
TDGF1
IGFBP6
ZFP42
C12orf35
FOXA2
TXNL1
8 pcw
9 pcw
12 pcw
13 pcw
16 pcw
17 pcw
19 pcw
21 pcw
24 pcw
25 pcw
26 pcw
35 pcw
37 pcw
4 mos
10 mos
1 yrs
2 yrs
3 yrs
4 yrs
8 yrs
11 yrs
13 yrs
15 yrs
18 yrs
19 yrs
21 yrs
23 yrs
30 yrs
36 yrs
37 yrs
40 yrs
C
Cx
HPC
Cx
CxN
Die
Mes
Cb
Pons
My
WM
SS
Adult Human Brain
IGFBP7
AASS
TM4SF18
FOXA2
IER3
PHLDA1
TXNL1
IGFBP6
TDGF1
PROS1
COL1A1
FABP5
WDR11
GDF15
TTR
C12orf35
DLK1
ZFP42
FLRT3

Appendix Figure S9A-C. Expression patterning of dysregulated genes from iPSCs. Heatmaps show localization of dysregulated gene expression in the progenitor structures (SVZ) of the prenatal human brain, the late weeks of postconception in the brain during development, and in the pons and myencephalon (My) adult human brain from iPSCs with **A-C**) *FXN* gene mutations. HTS, Hindbrain transient structures; SG, Subpial granular zone; SP, subplate zone; SS, sulci and spaces.

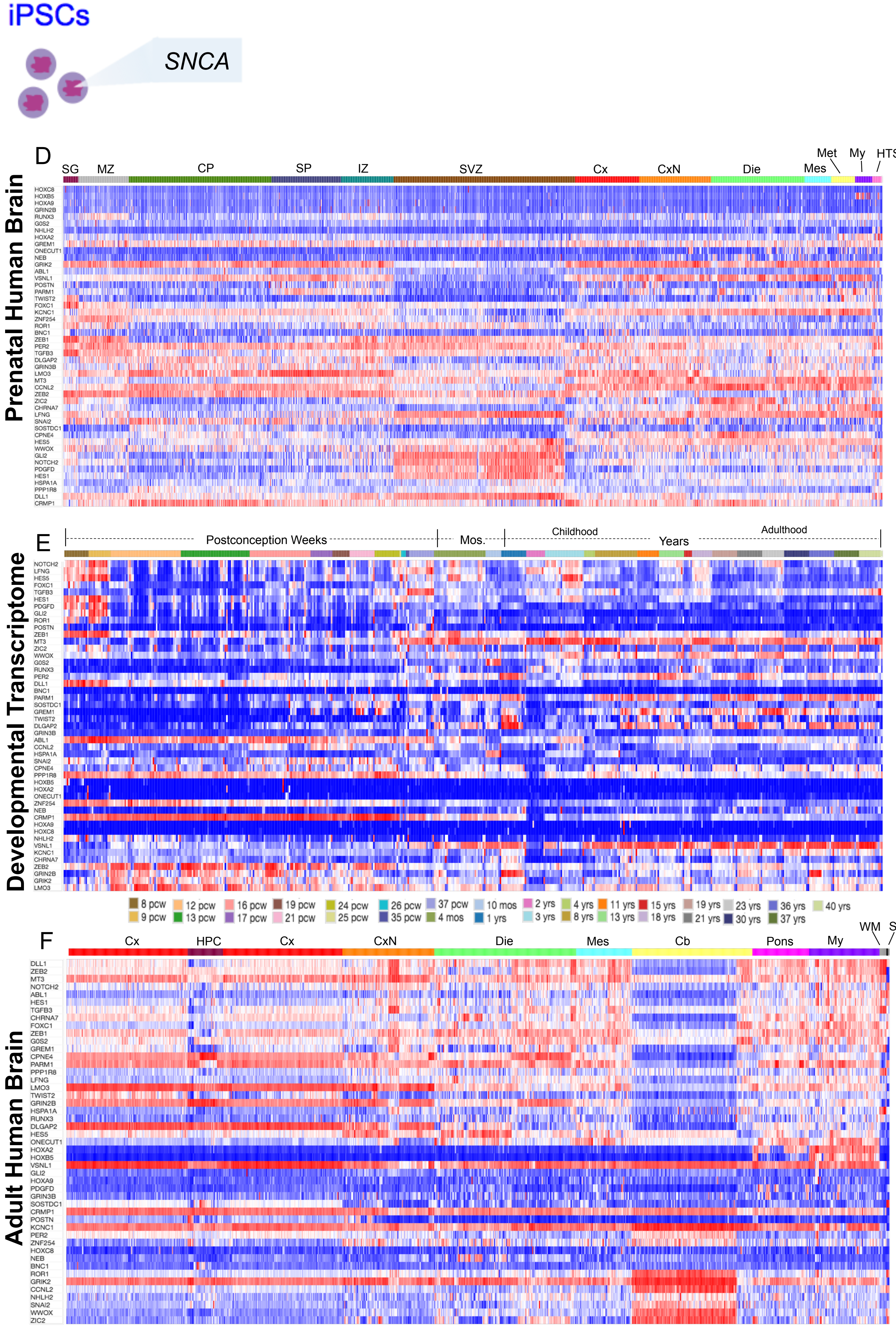

Appendix Figure S9D-F. Expression patterning of dysregulated genes from iPSCs. Heatmaps show localization of dysregulated gene expression in the developing cortex (CP, SP) and progenitor zones (SVZ) of the prenatal human brain, expression in the very early weeks of postconception during development, and in the cerebellum and globus pallidus (CxN) in the adult human brain from iPSCs with **D-F**) *SNCA* gene mutations. HTS, Hindbrain transient structures; SG, Subpial granular zone; SP, subplate zone; SS, sulci and spaces.

Appendix Table S1. **List of Published Studies Included in Meta-Analysis**

Disease Classification	First Author	Disease	Gene/ Mutation	Species	Year	Reprogramming Method	Starting Cell Type	Number of Disease Patients Studied	Number of Control Patients	Utilization of Gene-Editing or Isogenetic Cell Lines	Generated Cell Type	GEO ID	Platform Name	Platform GEO	PMID
Neurodegenerative	Muratore	Alzheimer's Disease	APP/ V717I	Homo sapiens	2014	Lentiviruses encoding Oct4, SOX2, c-MYC and KLF4	Fibroblast	2	4	No	Neurons	-	-	-	PMID: 24524897
Neurodegenerative	Kondo	Alzheimer's Disease	APP/ E693Δ, APP/ V717I, Sporadic/ AD8K213	Homo sapiens	2013	Human complementary DNA by episomal vectors	Fibroblast	3, 2, 2	3	No	Neurons, Astrocytes	GSE43326	HuGene-1_0-st	GPL6244	PMID: 23434393
Neurodegenerative	Yagi	Alzheimer's Disease	PSEN1/ A246E, PSEN2/ N141I	Homo sapiens	2011	Retroviral transduction of OCT4, SOX2, KLF4, LIN28 and NANOG	Fibroblast	1, 1	1	No	Neurons	GSE28450	Agilent-022060	GPL10123	PMID: 21900357
Neurodegenerative	Sproul	Alzheimer's Disease	PSEN1/ A246E	Homo sapiens	2014	Oct4, KLF4, Sox2, and c-Myc retroviruses	Fibroblast	4	1	No	NSCs	-	-	-	PMID: 24416243
Neurodegenerative	Koch	Alzheimer's Disease	PSEN1/ L166P	Homo sapiens	2012	Lentiviral Backbone (1-α (EF1α) promoter and IRES)	Fibroblast	1	1	No	Neurons	-	-	-	PMID: 22510327
Neurodegenerative	Isreal	Alzheimer's Disease	APP/ Duplication	Homo sapiens	2012	MMLV vectors containing the complementary DNAs for OCT4, SOX2 , KLF4, c-MYC and ± EGFP	Fibroblast	2	2	No	Neurons	-	-	-	PMID: 22278060
Neurodegenerative	Woodard	Parkinson's Disease	GBA1/ N370S	Homo sapiens	2014	Sendai virus of OCT4, SOX2, KLF4 and c-MYC	Fibroblast	2	4	Yes	Neurons	GSE62642	HiSeq 2500	GPL16791	PMID: 25456120
Neurodegenerative	Schöndorf	Parkinson's Disease	GBA1/ L444P, GBA1/ N370S	Homo sapiens	2014	Retroviruses encoding OCT4, SOX2, KLF4 an d c-MYC	Fibroblast	4	2	Yes	Neurons	-	-	-	PMID: 24905578
Neurodegenerative	Liu	Parkinson's Disease	LRRK2/ G2019S	Homo sapiens	2012	Retroviruses expressing OCT4, SOX2, KLF4	Fibroblast	2	1	Yes	NSCs	GSE34061	HiSeq 2000	GPL11154	PMID: 23075850
Neurodegenerative	Nguyen	Parkinson's Disease	LRRK2/ G2019S	Homo sapiens	2011	Retroviruses of OCT4, SOX2,	Fibroblast	1	1	No	Neurons	-	-	-	PMID: 21362567

						and KLF4									
Neurodegenerative	Reinhardt	Parkinson's Disease	LRRK2/ G2019S	Homo sapiens	2013	Retroviruses containing human Oct3/4, Sox2, Klf4, and c-Myc	Fibroblast	2	4	Yes	Neurons	GSE43364	illumina HumanHT-12 v4	GPL10558	PMID: 23472874
Neurodegenerative	Sanders	Parkinson's Disease	LRRK2/ G2019S, LRRK2/R1441C	Homo sapiens	2013	Retroviruses of OCT4, SOX2, KLF4 and CMYC	Fibroblast	5, 3	3	Yes	NSCs	-	-	-	PMID: 24148854
Neurodegenerative	Sánchez-Danés	Parkinson's Disease	LRRK2/ G2019S	Homo sapiens	2012	Retroviruses encoding FLAG-tagged OCT4, SOX2 and KLF4	Fibroblast	4	4	No	Neurons	-	-	-	PMID: 22407749
Neurodegenerative	Cooper	Parkinson's Disease	LRRK2/ G2019S, LRRK2/R1441C, PINK1/ Q456X	Homo sapiens	2012	Retroviruses of OCT4, SOX2, KLF4 and c-MYC	Fibroblast	3, 2, 2	2	No	Neurons	-	-	-	PMID: 22764206
Neurodegenerative	Reyes	Parkinson's Disease	SNCA/ Triplication	Homo sapiens	2014	Retroviruses of Oct4, Sox2, c-Myc, Klf4	Fibroblast	3	1	No	Neurons	-	-	-	PMID: 25046995
Neurodegenerative	Byers	Parkinson's Disease	SNCA/ Triplication	Homo sapiens	2011	Retroviral gene insertion of OCT4, SOX2, KLF4, and c-MYC	Fibroblast	1	1	No	Neurons	-	-	-	PMID: 22110584
Neurodegenerative	Devine	Parkinson's Disease	SNCA/ Triplication	Homo sapiens	2011	pMXs-cMyc #13375, pMXs-Klf4 #13370, pMXs-Oct4 #13366, pMXs-Sox2 #13367 reprogramming factors from Addgene	Fibroblast	1	1	No	Neurons	GSE28366	HumanOmni1-Quad	GPL8882	PMID: 21863007
Neurodegenerative	Chung	Parkinson's Disease	SNCA/ Triplication	Homo sapiens	2013	Cre-excisable polycistronic lentivirus encoding SOX2, KLF4, OCT4 and c-MYC	Fibroblast	1	1	Yes	Neurons	-	-	-	PMID: 24158904
Neurodegenerative	Ryan	Parkinson's Disease	SNCA/ A53T	Homo sapiens	2013	Retroviruses of OCT4, KLF4, SOX2, and c-MYC	Fibroblast	2	1	Yes	Neurons	GSE46798	Illumina HumanHT-12 v4	GPL10558	PMID: 24290359
Neurodegenerative	Jiang	Parkinson's Disease	Parkin/ Deletions on exons 3 and 5	Homo sapiens	2012	FUW-tetO-LoxP lentiviruses: hOct4, hSox4, hKlf4 and hNanog	Fibroblast	2	2	No	Neurons	GSE35190	RPCI Human HB21K	GPL15098	PMID: 22314364

Neurodegenerative	Imaizumi	Parkinson's Disease	Parkin/ Deletions on exons 3 and 5	Homo sapiens	2012	Retroviruses carrying Oct4, Sox2, Klf4, and c-Myc	Fibroblast	2	2	No	Neurons	-	-	-	PMID: 23039195
Neurodegenerative	Rakovic	Parkinson's Disease	PINK1/ Q456X	Homo sapiens	2012	Retroviral pMIG vectors OCT4, SOX2, cMYC and KLF4	Fibroblast	2	1	No	Neurons	-	-	-	PMID: 23212910
Neurodegenerative	Seibler	Parkinson's Disease	PINK1/ Q456X, PINK1/ V170G	Homo sapiens	2011	Retroviral pMIG vectors OCT4, SOX2, cMYC and KLF4	Fibroblast	3	1	No	Neurons	-	-	-	PMID: 21508222
Neurodevelopmental	Xia	Dystrophia Myotonica type 1	DMPK/ CTG repeats	Homo sapiens	2013	Retroviral transduction of hOct4, hSox2, hKlf4, and hc-Myc	Fibroblast	2	1	No	Neurons, NSCs, Astrocytes , iPSCs	-	-	-	PMID: 23550732
Neurodegenerative	Corti	Spinal Muscular Atrophy	SMN1/ not specified	Homo sapiens	2012	oriP/EBNA1-based episomal vectors of OCT4, SOX2, NANOG, LIN28, c-Myc, and KLF4 by nucleofection	Fibroblast	2	2	No	Neurons	GSE27206	HG-U133_Plus_2	GPL570	PMID: 23253609
Neurodegenerative	Chang	Spinal Muscular Atrophy	SMN1/ not specified	Homo sapiens	2011	Retroviral vectors containing Oct4, Sox2, c-Myc, Klf4	Fibroblast	1	1	Yes	Neurons	-	-	-	PMID: 21956898
Neurodegenerative	Ebert	Spinal Muscular Atrophy	SMN1/ not specified	Homo sapiens	2009	Lentiviral infection of primers for OCT 4, SOX 2, NANOG, LIN 28, HoxB4, SMN, and GAPDH	Fibroblast	1	1	No	Neurons, Astrocytes	GSE13828	HG-U133_Plus_2	GPL570	PMID: 19098894
Neurodegenerative	Sareen	Spinal Muscular Atrophy	SMN1/ not specified	Homo sapiens	2012	Lentiviral infection of primers for OCT 4, SOX 2, NANOG, LIN 28, HoxB4, SMN, and GAPDH	Fibroblast	2	1	No	Neurons	-	-	-	PMID: 22723941

Viral Infection	Lafaille	Childhood HSE	UNC-93-B/ not specified, TLR3/ not specified	Homo sapiens	2012	Polycistronic lentiviral pHAGE–STEMCCA–LoxP vector, carrying the OCT4, SOX2, KLF4, and C-MYC reprogramming factor genes	Fibroblast	1, 1	1	No	Neurons, NSCs, Astrocytes, Oligodendrocytes	GSE40593	Illumina HumanWG-6 v3.0	GPL6884	PMID: 23103873
Neurodevelopmental	Marchetto	Rett Syndrome	MeCP2/ Q244X	Homo sapiens	2010	Retrovirus vectors containing the Oct4, c-Myc, Klf4 and Sox2	Fibroblast	4	5	No	Neurons	GSE21037	HuGene-1_0-st	GPL6244	PMID: 21074045
Neurodevelopmental	Kim	Rett Syndrome	MeCP2/ Q244X	Homo sapiens	2011	pMIG retrovirus expressing OCT4, SOX2, KLF4, and MYC	Fibroblast	5	5	No	Neurons, NSCs, iPSCs	-	-	-	PMID: 21807996
Neurodevelopmental	Ricciardi	Rett Syndrome	CDKL5/ R59X, CDKL5/ L220P	Homo sapiens	2012	Retrovirus infection of OCT4, SOX2 and KLF4	Fibroblast	2, 2	2	No	Neurons	-	-	-	PMID: 22922712
Neurodevelopmental	Cheung	Rett Syndrome	MeCP2/ deletions on exons 3 and 4, MeCP2/ T158M, MeCP2/ R306C,	Homo sapiens	2011	Lentiviral infection with EOS-vector and MoMLV-based retroviral vectors encoding OCT4, SOX2, KLF4 and c-MYC	Fibroblast	1,3,3	1,1,1	Yes	iPSCs	-	-	-	PMID: 21372149
Neurodegenerative	Koch	Spinocerebellar Ataxia Type 3	ATXN3/ CAG repeats	Homo sapiens	2011	Retroviral plasmids for OCT4, SOX2, KLF4 and c-MYC	Fibroblast	1	2	No	Neurons	-	-	-	PMID: 22113611
Neurodegenerative	Almeida	Frontotemporal dementia	PGRN/ S116X	Homo sapiens	2012	Retroviruses encoding human *OCT3/4*, *SOX2*, *KLF4*, and *c-MYC*	Fibroblast	1	1	No	Neurons	GSE40378	illumina HumanHT-12 v4	GPL10558	PMID: 23063362
Neurodegenerative	Almeida	Frontotemporal dementia/ Amyotrophic lateral sclerosis	C9ORF72/ GGGGCC repeats	Homo sapiens	2013	Retroviruses expressing human OCT3/4, SOX2, KLF4, and c-MYC	Fibroblast	2	1	No	Neurons	-	-	-	PMID: 23836290
Neurodegenerative	Sareen	Frontotemporal dementia/	C9ORF72/ GGGGC	Homo sapiens	2013	pCXLE-hUL, pCXLE-hSK, and pCXLE-	Fibroblast	4	4	No	Neurons, NSCs, Astrocytes	GSE52202	HiSeq 2000	GPL11154	PMID: 24154603

		Amyotrophic lateral sclerosis	C repeats			hOCT3/4-shp53-F vectors									
Neurodegenerative	Bilican	Amyotrophic lateral sclerosis	TDP-43/ M337V	Homo sapiens	2012	Retroviral pMIG vectors OCT4, SOX2, cMYC and KLF4	Fibroblast	1	2	No	Neurons	-	-	-	PMID: 22451909
Neurodegenerative	Egawa	Amyotrophic lateral sclerosis	TDP-43/ M337V, TDP-43/ Q343R, TDP-43/ G298S	Homo sapiens	2012	Retrovirus of Sox2, Klf4, Oct3/4, and/or c-Myc or episomal vectors of Sox2, Klf4, Oct3/4, L-Myc, Lin28, and short hairpin RNA for p53	Fibroblast	3, 3, 3	5	No	Neurons	-	-	-	PMID: 22855461
Neurodegenerative	Serio	Amyotrophic lateral sclerosis	TDP-43/ M337V	Homo sapiens	2013	Retroviruses encoding Oct4, Sox2, Klf4 and Myc	Fibroblast	1	2	No	Astrocytes	-	-	-	PMID: 23401527
Neurodegenerative	Chen	Amyotrophic lateral sclerosis	SOD1/ D90A, SOD1/ A4V	Homo sapiens	2014	Nonintegrating Sendai virus of OCT4, SOX2, KLF4 and c-MYC, Retroviral plasmids for OCT4, SOX2, KLF4 and c-MYC	Fibroblast	1, 1	2	Yes	Neurons	-	-	-	PMID: 24704493
Neurodegenerative	Kiskinis	Amyotrophic lateral sclerosis	SOD1/ A4V	Homo sapiens	2014	Retroviral transduction of *SOX2*, *OCT3/4*, *KLF4* and *C-MYC*	Fibroblast	2	2	Yes	Neurons	GSE54409	HiSeq 2000	GPL11154	PMID: 24704492
Neurodevelopmental	Pasca	Timothy Syndrome	CACNA1C/ Ca_V1.2	Homo sapiens	2012	Retroviruses expressing *SOX2*, *OCT3/4*, *KLF4* and *C-MYC*	Fibroblast	2	3	No	Neurons	GSE25542	Illumina HumanRef-8 v3.0	GPL6883	PMID: 22120178
Neurodegenerative	Zhang	Huntington's Disease	HTT/ CAG repeats	Homo sapiens	2010	VSVg retroviruses of Sox2, Klf4, Oct3/4, and/or c-Myc	Fibroblast	1	1	No	NSCs	-	-	-	PMID: 21037797
Neurodegenerative	An	Huntington's Disease	HTT/ CAG repeats	Homo sapiens	2012	VSVg retroviruses of Sox2, Klf4, Oct3/4, and/or c-Myc	Fibroblast	1	1	Yes	NSCs	GSE37547	Nimblegen Homo sapiens HG18 (12x135k)	GPL15338	PMID: 22748967
Neurodegenerative	Jeon	Huntington's Disease	HTT/ CAG	Homo sapiens	2012	VSVg retroviruses of	Fibroblast	1	3	No	iPSCs	-	-	-	PMID: 22628015

			repeats			Sox2, Klf4, Oct3/4, and/or c-Myc									
Neurodegenerative	Juopperi	Huntington's Disease	HTT/ CAG repeats	Homo sapiens	2012	Retroviral transduction of Oct3/4, Sox2, c-MYC and Klf4	Fibroblast	2	1	No	Astrocytes	-	-	-	PMID: 22613578
Neurodegenerative	Chae	Huntington's Disease	HTT/ CAG repeats	Homo sapiens	2012	VSVg retroviruses of Sox2, Klf4, Oct3/4, and/or c-Myc	Fibroblast	2	2	No	iPSCs	-	-	-	PMID: 22694310
Neurodegenerative	Guo	Huntington's Disease	HTT/ CAG repeats	Homo sapiens	2013	VSV-g pseudotyped hSTEMCCA lentivirus containing a polycistronic coding sequence for Sox2, Klf4, Oct3/4, and/or c-Myc	Fibroblast	2	2	No	Neurons	-	-	-	PMID: 24231356
Neurodegenerative	HD iPSC Consortium	Huntington's Disease	HTT/ CAG repeats	Homo sapiens	2012	Lentiviral transduction of *Oct4, Sox2, Klf4, cMyc, Nanog* and *Lin28*	Fibroblast	2	2	No	Neurons, NSCs	GSE37517	HuGene-1_0-st	GPL10739	PMID: 22748968
Neurodevelopmental	Jang	X-linked adrenoleukodystrophy	ABCD1 or ALDP/AMN patient, ABCD1 or ALDP/ CCALD patient	Homo sapiens	2011	Retroviral plasmids for OCT4, SOX2, KLF4 and c-MYC	Fibroblast	1, 1	1	No	Neurons, Oligodendrocytes	-	-	-	PMID: 21721033
Neurodevelopmental	Liu	Dravet Syndrome	SCN1A/ IVS14+3 A>T	Homo sapiens	2013	Retroviral infection of pMXs-Oct3/4, pMXs-Sox2, pMXs-Klf4, pMXs-c-Myc	Fibroblast	2	3	No	Neurons	-	-	-	PMID: 23821540
Neurodevelopmental	Higurashi	Dravet Syndrome	SCN1A/ mutation on 4933C>T	Homo sapiens	2013	Retroviral plasmids for OCT4, SOX2, KLF4 and c-MYC	Fibroblast	1	1	No	Neurons	-	-	-	PMID: 23639079
Neurodevelopmental	Liu	Hutchinson-Gilford progeria syndrome	LMNA/ point mutation	Homo sapiens	2011	Retroviruses encoding OCT4, SOX2, KLF4, c-MYC, and GFP	Fibroblast	1	2	No	iPSCs	GSE24487	HG-U133_Plus_2	GPL570	PMID: 21346760

Neurodegenerative	Hick	Friedreich's Ataxia	FXN/ expanded GAA repeats	Homo sapiens	2012	Lentivirus factors OCT4, SOX2, LIN28 and NANOG	Fibroblast	2	2	No	Neurons, iPSCs	-	-	-	PMID: 23136396
Neurodegenerative	Sherman	Friedreich's Ataxia	FXN/ Long GAA·TTC repeats	Homo sapiens	2010	VSV-G Retrovirus vectors for Oct3/4, Sox2, Klf4, and c-Myc	Fibroblast	2	1	No	iPSCs	GSE22651	Illumina HumanHT-12 V3.0	GPL6947	PMID: 21040903
Neurodevelopmental	Andrade	Cockayne syndrome	ERCC6/ not specified	Homo sapiens	2012	pMXs retroviral vectors containing the human cDNA for c-Myc, Klf4, Oct3/4, Sox2 VSGV and CMVgp	Fibroblast	1	2	No	Neurons, iPSCs	GSE36648	HuGene-1_0-st	GPL6244	PMID: 22661500
Neurodegenerative	Fong	Tauopathies	MAPT/ A152T	Homo sapiens	2013	Retroviral plasmids for OCT4, SOX2, KLF4 and c-MYC	Fibroblast	1	1	Yes	Neurons	-	-	-	PMID: 24319659
Neurodevelopmental	Numasawa-Kuroiwa	Pelizaeus-Merzbacher disease	PLP1/ missense	Homo sapiens	2014	Retroviral plasmids for OCT4, SOX2, KLF4 and c-MYC	Fibroblast	2	2	No	Oligodendrocytes	-	-	-	PMID: 24936452
Neurodevelopmental	Doers	Fragile X Syndrome	FMR1/ CGG repeats	Homo sapiens	2014	Retroviral transduction of Oct3/4, Sox2, c-MYC and Klf4	Fibroblast	3	1	Yes	Neurons	-	-	-	PMID: 24654675
Neurodevelopmental	Sheridan	Fragile X Syndrome	FMR1/ CGG repeats	Homo sapiens	2011	Retroviral pMIG vectors OCT4, SOX2, cMYC and KLF4	Fibroblast	3	2	No	Neurons	-	-	-	PMID: 22022567
Neurodevelopmental	Suh	Menkes Disease	ATP7A/ c4005 + G > A	Homo sapiens	2014	Retroviral transduction of Oct3/4, Sox2, c-MYC and Klf4	Fibroblast	1	1	No	iPSCs	-	-	-	PMID: 24468087
Neurodevelopmental	Higuchi	Pompe Disease	GAA/ not specified	Homo sapiens	2014	pMX retrovirus vectors OCT3/4, SOX2, KLF4, and c-MYC.	Fibroblast	2	1	No	iPSCs	-	-	-	PMID: 24642446
Neurodevelopmental	Maetzel	Niemann-Pick Type C	NPC1/ I1061T	Homo sapiens	2014	Lentiviruses encoding a polycistronic doxcycycline-inducible (DOX) for Oct4, Klf4, Sox2, c-Myc	Fibroblast	4	2	Yes	Neurons	-	-	-	PMID: 24936472
Neurodevelopmental	Mazzulli	Gaucher's Disease	GBA1/ not	Homo sapiens	2011	Retroviral pMIG vectors OCT4,	Fibroblast	2	3	No	iPSCs, Neurons	-	-	-	PMID: 21700325

			specified			SOX2, cMYC and KLF4									
Neurodevelopmental	Ananiev	Rett Syndrome	MeCP2/ R294X, MeCP2/ T158M, MeCP2/ V247X, MeCP2/ R306C	Homo sapiens	2011	Retroviral plasmids for OCT4, SOX2, KLF4 and c-MYC	Fibroblast	1, 1, 1, 1	1	Yes	Neurons, iPSCs	-	-	-	PMID: 21966470
Neurodevelopmental	Panicker	Gaucher's Disease	GBA1/ L444P, GBA1/ N370S	Homo sapiens	2012	Retroviral plasmids for OCT4, SOX2, KLF4 and c-MYC	Fibroblast	2, 1	2	No	Neurons	-	-	-	PMID: 23071332
Neurodevelopmental	Krey	Timothy Syndrome	CACNA1 C/ $Ca_V1.2$	Homo sapiens	2013	Retroviral transduction of Oct3/4, Sox2, c-MYC and Klf4	Fibroblast	2	2	No	Neurons	-	-	-	PMID: 23313911
Psychiatric	Yoon	Schizophrenia	CYFIP1/ 15q11.2	Homo sapiens	2014	Sendai Viral Infection	Fibroblast	3	3	No	NSCs	-	-	-	PMID: 24996170
Psychiatric	Wen	Schizophrenia/ Major Depressive Disorder	DISC1/ 4bp C-terminus deletion	Homo sapiens	2014	Non-integrating EBV-based vector transfection	Fibroblast	2	3	Yes	Neurons	GSE57821	Ion Torrent Proton	GPL17303	PMID: 25132547
Neurodegenerative	Liu	Alzheimer's Disease	APP/ Duplication, PSEN1/A 246E, PSEN1/ H163R, PSEN1/ M146L	Homo sapiens	2014	Maloney Murine Leukemia Virus infection of Oct3/4,Sox2, Klf4, and c-Myc	Fibroblast	1, 2, 1, 1	2	No	Neurons, NSCs, iPSCs	-	-	-	PMID: 25285942
Neurodegenerative	Ren	Parkinson's Disease	Parkin/ Deletions on exons 3 and 5	Homo sapiens	2015	Lentiviruses expressing human Oct4, Sox2, Klf4, c-Myc and Nanog	Fibroblast	2	2	No	Neurons	-	-	-	PMID: 25332110
Neurodegenerative	Devlin	Amyotrophic lateral sclerosis	TDP-43/M337 V, C9ORF72/ GGGGCC repeats	Homo sapiens	2015	Lentivirus or Sendai vectors for OCT4, SOX2, KLF4 and c-MYC	Fibroblast	1, 2	3	No	Neurons	-	-	-	PMID: 25580746
Neurodevelopmental	Efthymiou	Niemann-Pick Type C	NPC1 / I1061T	Homo sapiens	2015	STEMCCA lentivirus system	Fibroblast	1	1	No	Neurons	GSE55379	Illumina HumanHT-12 V4.0	GPL10558	PMID: 25637190
Neurodegenerative	Liu	Amyotrophi	FUS/P52	Homo	2015	Nucleofected	Fibroblast	1	1	No	Neurons	-	-	-	PMID: 25912081

		c lateral sclerosis	5L	sapiens		with 15–20 µg EVplasmid DNA using a human CD34+ Cell Nucleofector™ Kit									
Neurodegenerative	Lenzi	Amyotrophic lateral sclerosis	FUS/R514S, FUS/R521C, FUS/P525L	Homo sapiens	2015	Lentiviral vector hSTEMCCA for OCT4, SOX2, KLF4 and c-MYC	Fibroblast	1, 1, 1,	2	Yes	Neurons, iPSCs	-	-	-	PMID: 26035390
Neurodegenerative	Ehrlich	Frontotemporal dementia	MAPT/ N279K, MAPT/ V337M	Homo sapiens	2015	Lentiviral transduction with a polycistronic vector encoding *OCT4*, *KLF4*, *SOX2*, and *C-MYC*	Fibroblast	1, 1	3	No	Neurons	-	-	-	PMID: 26143746
Neurodegenerative	Liu	Spinal Muscular Atrophy	SMN1/ Deletion of exons 7 and 8	Homo sapiens	2015	Retrovirus for OCT4, SOX2, KLF-4 and c-MYC	Fibroblast	3	3	No	Neurons	-	-	-	PMID: 26190808
Neurodevelopmental	Shcheglovitov	Phelan-McDermid Syndrome	SHANK3 / Deletion	Homo sapiens	2013	Retroviruses carrying SOX2, OCT3/4, c-MYC and KLF4 transcription factors	Fibroblast	2	2	No	iPSCs, Neurons	-	-	-	PMID: 24132240
Neurodegenerative	HD iPSC Consortium	Huntington's Disease	HTT / 46 CAG repeats, HTT / 53 CAG repeats, HTT / 109 CAG repeats	Homo sapiens	217	Non-integrating episomal plasmids containing OCT4, SOX2, KLF4, L-MYC, LIN28, and p53	Fibroblast	2,2,2	4,4,4	No	Neurons	GSE95344	Illumina HiSeq 2000)	GPL11154	PMID: 28319609
Neurodegenerative	Grima	Huntington's Disease	HTT / 53 CAG repeats, HTT / 109 CAG repeats	Homo sapiens	2017	Non-integrating episomal plasmids containing OCT4, SOX2, KLF4, L-MYC, LIN28, and p53	Fibroblast	1,1	1,1	No	Neurons	-	-	-	PMID: 28384479
Neurodevelopmental	Bidinosti	Phelan-McDermid Syndrome	SHANK3 / Deletions	Homo sapiens	2016	Nonintegrating Sendai virus of OCT4, SOX2, KLF4 and c-MYC	Fibroblast	2	2	No	Neurons	-	-	-	PMID: 26847545

Neurodegenerative	Sivadasan	Amyotrophic lateral sclerosis	C9ORF72 / intronic GGGCC repeats	Homo sapiens	2016	Retroviruses containing human Oct3/4, Sox2, Klf4, and c-Myc	Fibroblast	2	2	No	Neurons	-	-	-	PMID: 27723745
Neurodevelopmental	Francis	Smith-Lemli-Opitz syndrome	DHCR7 / p.M1V; p.Q98X, DHCR7 / p.T154M; c.964-1G>C, DHCR7 / p.T93M; c.964-1G>C, DHCR7 / c.964-1G>C	Homo sapiens	2016	Lentiviral transduction with loxP-flanked STEMCCA polycistronic vector	Fibroblast	2,2,4,3	4,4,4,4	No	iPSCs	GSE61203	Affymetrix Human Exon 1.0 ST Array	GPL5175	PMID: 26998835
Neurodegenerative	Dickey	Huntington's Disease	HTT / 60 CAG repeats	Homo sapiens	2016	Lentiviral transduction of Oct4, Sox2, Klf4 , cMyc, Nanog, and Lin28	Fibroblast	1	1	No	Neurons	-	-	-	PMID: 26642438
Neurodegenerative	Martinez	Multisystem proteinopathy	hnRNPA2/B1 / D290V, VCP / R155H	Homo sapiens	2016	Nonintegrating Sendai virus of OCT4, SOX2, KLF4 and c-MYC	Fibroblast	3, 2	1, 1	No	Neurons	GSE86464	Affymetrix Human Transcriptome Array 2.0	GPL17585	PMID: 27773581
Neurodegenerative	Orellana	Pantothenate kinase-associated neurodegeneration	PANK2 / c.1259delG, PANK2 / c.569insA	Homo sapiens	2016	Nonintegrating Sendai virus of OCT4, SOX2, KLF4 and c-MYC	Fibroblast	2, 1	3, 3	No	NSCs, Neurons	-	-	-	PMID: 27516453
Neurodegenerative	Silva	Frontotemporal dementia	MAPT / A152T	Homo sapiens	2016	Retroviral vector infection containing human Oct4, Sox2, Klf4, and c-Myc	Fibroblast	2	2	No	Neurons	-	-	-	PMID: 27594585
Neurodevelopmental	Aflaki	Gaucher Disease	GBA1 / N370S, GBA1 / IVS2+1G>T; L444P	Homo sapiens	2016	Cre-excisable polycistronic lentivirus encoding SOX2, KLF4, OCT4 and c-MYC	Fibroblast	4, 1	1, 1	No	NSCs, Neurons	-	-	-	PMID: 27413154
Neurodegenerative	Stanslowsky	Chorea-Acanthocytosis	VPS13A / c.4282G>C, VPS13A	Homo sapiens	2016	Retroviral transduction of Oct4, c-MYC Sox2, Klf4	Fibroblast	1, 1	2, 2	No	Neurons	-	-	-	PMID: 27881786

			/ c.7806G >A												
Neurodevelopmental	Trilck	Niemann-Pick disease Type C	NPC1 / c.3182T>C, NPC1 / c.1836A >C/c.1628delC	Homo sapiens	2017	Retroviral transduction of Oct4, c-MYC Sox2, Klf4	Fibroblast	1, 1	1, 1	No	Neurons	-	-	-	PMID: 27923633
Neurodegenerative	Hallman	Frontotemporal dementia	MAPT / N279K	Homo sapiens	2017	Lentiviral transduction with a polycistronic vector encoding OCT4, KLF4, SOX2, and C-MYC	Fibroblast	1	1	Yes	Neurons, Astrocytes	-	-	-	PMID: 28256506
Neurodevelopmental	Vessoni	Cockayne Syndrome	ERCC6 / not specificied	Homo sapiens	2016	Non-integrating Sendai vectors containing OCT4,KLF4, SOX2, and C-MYC	Fibroblast	2	2	No	Neurons	-	-	-	PMID: 26755826

Note: In cases with multiple genes studied, the patient quantities reported under the “Number of Disease Patients Studied” are listed in the same order as the “Gene/ Mutation” column.

Appendix Table S2. **Categorical Cluster Descriptions**	
Impairment of expected cellular functions	This category contains any phenotype that can be described by the presence of a disrupted/changed state of a structure or process that is expected and found in a healthy version of the same cell and cannot be described in terms of increases or decreases. i.e. Impaired structure of adherens junctions (PMID: 24996170)
Absence of expected normal phenotype	This category contains any phenotype that can be described by the complete loss of a function that is found in a healthy version of the same cell. i.e. Absence of random X-inactivation (PMID: 21372419)
Decreased cellular processes and products	This category contains any phenotype that can be described by a decrease in the rate and/or the decreased output of any process and the products it creates within a cell. i.e. Decreased levels of co-localized LC3/LAMP-1 (PMID: 22407749)
Increased susceptibility to chemical exposure	This category contains any phenotype that can be described by an increase in a cell's susceptibility to death and other harmful events after being exposed to a specific chemical. i.e. Increased susceptibility of neurons to valinomycin (PMID: 22764206)
Rescue/recovery from disease phenotypes after chemical treatment	This category contains any phenotype that can be described by the returning of a disease phenotype to the normal healthy cellular phenotype after the cell is treated with a certain chemical. i.e. Rescue of aberrant cellular parameters after treatment with LRRK2-In-1 (PMID: 23075850)
Presence of abnormal cellular structures	This category contains any phenotype that can be described by the observation of a disrupted or altered cellular structure not observed in a healthy cell. i.e. Presence of constricted/ tapered neurites (PMID: 24319659)

Decreased susceptibility to chemical exposure	This category contains any phenotype that can be described by the decreased in a cell's susceptibility to death and other harmful events after being exposed to a specific chemical. i.e. Decreased susceptibility to PI3K inhibitor after ectopic progranulin expression (PMID: 23063362)
Increased cellular processes and products	This category contains any phenotype that can be described by an increase in the rate and/or the increased output of any process and the products it creates within a cell. i.e. Increased spontaneous dopamine release (PMID: 22314364)
Accumulation of molecules	This category contains any phenotype that can be described by the abnormal accumulation of molecules in a cell not seen in healthy cells. i.e. Accumulation of α-synuclein (PMID: 24905578)

Appendix Table S3. **Phenotypic Distribution of Categorical Clusters Based on Developmental Stage**						
Phenotype Categories	Cell Type					
	Neurons	NSCs	Astrocytes	Oligodendrocytes	iPSCs	Total
Rescue/Recovery from Disease Phenotypes After Chemical Treatment	21	2	0	2	0	25
Accumulation of Molecules	22	1	3	0	5	31
Absence of Expected Normal Phenotypes	4	0	1	0	4	9
Presence of Abnormal Cellular Structures	20	2	1	6	9	31
Impairment of Expected Cellular Functions	23	5	1	4	2	35
Decreased Cellular Processes and Products	197	18	6	4	22	247
Decreased Susceptibility to Chemical Exposure	11	0	0	1	0	12
Increased Cellular Processes and Products	181	10	17	5	19	232
Increased Susceptibility to Chemical Exposure	27	1	1	3	2	34
Total	506	39	22	25	63	663

Appendix Table S4. **MeV Plot Phenotypes with Identifying Numbers**	
Number	Phenotype
1	Impairment of neuronal induction of IFN-β and IFN-γ in response to HSV-1 infection
2	Impairment of neuronal induction of IFN-β and IFN-γ in response to polyinosinic:polycytidylic acid
3	Increased susceptibility of neurons to HSV-1 infection
4	Decreased susceptibility of neurons to HSV-1 infection after treatment with exogenous IFN-α/ β
5	Increased replication speed of HSV-1-GFP in neurons
6	Rescued replication speed of HSV-1-GFP in neurons after treatment with IFN-α2b
7	Rescued replication speed of HSV-1-GFP in neurons after treatment with IFN-β
8	Decreased amount of basal autophagy in neurons
9	Increased amount of autophagosomes in neurons
10	Presence of fragmented nuclei in neurons
11	Impaired nuclear architecture in neurons
12	Decreased survival of neurons
13	Decreased motor neuron survival
14	Impairment of neuronal maturation
15	Decreased dopamine reuptake by neurons
16	Increased spontaneous dopamine release by neurons
17	Increased secretion of dopamine in neurons
18	Increased secretion of norepinephrine in neurons
19	Decreased amount of neurons expressing lower layer markers (FOXP1 and ETV1)
20	Increase in sustained calcium ion rise following depolarization of neurons
21	Impaired calcium channel inactivation in neurons
22	Decreased amount of DAT-binding sites on neurons
23	Decreased electrical excitability of neurons
24	Decreased spike size upon depolarization of neurons
25	Increased sodium current in neurons
26	Decrease in frequency/ amplitude of spontaneous postsynaptic currents of neurons
27	Impaired neuronal network connectivity in glutamatergic neurons
28	Decreased amount of neuronal synapses
29	Decreased neuromuscular junction size of neurons
30	Decreased amount of the density of V-GLUT1 puncta in dendrites of neurons
31	Increased amount of large Rab5+ early endosomes in neurons
32	Presence of vacuolated soma in neurons
33	Presence of Lewy neurite/body-like α-synuclein deposition in neurons
34	Decreased soma size of glutamatergic neurons
35	Decreased spine density in glutamatergic neurons
36	Presence of fewer/smaller end plates in motor neurons
37	Decrease in growth cone size in neurons
38	Decreased neuron size
39	Decrease in mean length of axons in neurons
40	Decrease in length/number of processes in neurons

41	Decreased neurite outgrowth by forebrain neurons
42	Decrease in length/number of processes extended by forebrain neurons
43	Decreased motility/slow rate of neurite extension in forebrain neurons
44	Decreased neurite outgrowth in neurons
45	Decreased average neurite length of DA neurons after exposure to rapamycin
46	Decreased average neurite length of DA neurons after exposure to leupeptin
47	Accumulation of monomeric α-synuclein in neurons
48	Accumulation of oligomerized α-synuclein in neurons
49	Accumulation of monomeric α-synuclein in midbrain dopaminergic neurons
50	Increase in α-synuclein immunoreactivity in midbrain dopaminergic neurons
51	Accumulation of TDP-43 in neurons
52	Increased percentage of neurons containing cytoplasmic TDP-43
53	Increased percentage of neurons with cytoplasmic TDP-43 after exposure to tunicamycin
54	Increased percentage of neurons with cytoplasmic TDP-43 after exposure to staurosporine
55	Increased levels of soluble and detergent-resistant TDP-43 protein in neurons
56	Presence of SDS-insoluble ATXN3-containing fragments generation in neurons
57	Partial impairment of γ-secretase function in neurons
58	Accumulation of Aβ oligomers in neurons
59	Decreased amount of Aβ-oligomers in neurons with β-secretase inhibitor treatment
60	Increased Aβ42/40 ratio in neurons
61	Increased extracellular Aβ42 in neurons
62	Increased secretion of Aβ (1-40) by neurons
63	Increase in basal levels of NO (RNS) in neurons
64	Increased accumulation of NO in neurons after exposure to mitochondrial toxins (Paraquat or Maneb)
65	Impaired reduction of mtDNA upon mitochondrial depolarization in neurons
66	Increase in passive leak of protons from inner mitochondrial membrane of neurons
67	Decrease in mitochondrion length in the proximal axon of neurons
68	Increase in mobility of mitochondria in the proximal axon of neurons
69	Increase in bidirectional movement of mitochondria in the proximal axon of neurons
70	Decreased recruitment of Parkin to mitochondria in neurons
71	Increased electron density in matrix of the inner mitochondrial membrane of neurons
72	Increased perikaryal volume in mitochondria of neurons
73	Increased swelling of mitochondrial cristae in the inner mitochondrial membrane of neurons
74	Presence of energy substrate (ATP)- independent respiration in neurons
75	Impaired maximal rate of mitochondrial respiration in neurons
76	Increased ROS levels in basal conditions in neurons
77	Decreased mROS production in neurons exposed to low concentrations of valinomycin after treatment with rapamycin

78	Decreased Basal Oxygen Consumption Rate (OCR) in neurons
79	Increased Basal Oxygen Consumption Rate (OCR) in neurons
80	Absence of nuclear gems (SMN protein nuclear foci) in neurons
81	Decreased levels of extracellular Aβ42 in neurons
82	Decreased levels of extracellular Aβ40 in neurons
83	Decreased cell viability in neurons
84	Partial rescue of cell viability in neurons after treatment with DHA
85	Increased levels of binding protein (BiP) in neurons after treatment with high concentrations of DHA
86	Decreased levels of binding protein (BiP) in neurons after treatment with DHA
87	Decreased levels of ROS in neurons after treatment with DHA
88	Decreased levels of cleaved caspase-4 in neurons after treatment with DHA
89	Decreased levels of binding protein (BiP) in neurons after treatment with β-secretase inhibitor
90	Decreased levels of cleaved caspase-4 in neurons after treatment with β-secretase inhibitor
91	Accumulation of RNA foci in neurons
92	Presence of co-localization of RNA foci in neurons with hnRNPA1
93	Presence of co-localization of RNA foci in neurons with Pur-α
94	Decreased formation of RNA foci in neurons after treatment with antisense oligonucleotides
95	Accumulation of intranuclear RNA foci in neurons
96	Increased susceptibility of neurons to antagonism of the PI3K pathway
97	Increased activation of Nrf2 pathway in neurons
98	Impairment of induced ubiquitination of Mfn2 protein after treatment with valinomycin in neurons
99	Increased damage to neurons by inhibition of SOH-MEF2C (Sulfenated-myocyte enhancer factor 2C) after exposure to hydrogen peroxide
100	Increase in LC3-positive puncta (antibody) in neurons
101	Increased positive staining for cleaved caspase-3 in neurons
102	Increased levels of nucleoporin p62 protein in neurons
103	Increased abundance of autophagic vacuoles in neurons
104	Increased levels of pERK in neurons
105	Increased levels of TAU protein in neurons
106	Increased levels of phospho-Thr181 TAU in neurons
107	Increased amount of anti-glycogen synthase kinase 3 beta (aGSK-3β) in neurons
108	Increased p-tau/total tau ratios in neurons
109	Increased MAO A and B transcription in neurons
110	Increase in PGC-1α levels in neurons
111	Increase in very long chain fatty acid (C26:0/C22:0 ratio) levels in neurons
112	Increase in fraction of neurons expressing TH
113	Increased proportion of CTIP2 expressing neurons in lower layer marker (FOXP1 and ETV1) expressing neurons
114	Increased levels of cleaved caspase-4 in neurons
115	Increased levels of binding protein (BiP) in neurons
116	Increased caspase-3 activity in neurons after exposure to staurosporine
117	Increased caspase-3 activity in neurons after exposure to tunicamycin
118	Decreased amount of outer mitochondrial membrane (OMM) proteins of

	increased molecular mass in neurons
119	Decreased protein expression levels of NURR1 and TH in neurons
120	Decreased levels of co-localized LC3/LAMP-1 in neurons
121	Decreased levels of SNCG expression in neurons
122	Decreased expression of C9ORF72 in neurons
123	Increased levels of S6K2 in neurons after ectopic progranulin expression
124	Decreased S6K2 protein levels in neurons
125	Decreased proportion of SATB2 expressing neurons in lower layer marker (FOXP1 and ETV1) expressing neurons
126	Decreased ATXN3 aggregate formation in neurons after treatment with calpeptin (calpain inhibitor)
127	Decreased ATXN3 aggregate formation in neurons after treatment with ALLN (calpain inhibitor)
128	Decreased amount of extracellular Aβ 42 by neurons by treatment with a selective Aβ42-lowering agent
129	Decreased amount of extracellular Aβ 42 by neurons after treatment with γ-secretase inhibitor
130	Increased amount of Aβ42 in neurons
131	Decreased amount of Aβ42 in neurons after treatment with γ-secretase modulator
132	Decreased amount of Aβ40 in neurons after treatment with γ-secretase modulator
133	Decreased amount of Aβ38 in neurons after treatment with γ-secretase modulator
134	Decreased Aβ42:Aβ40 ratio in neurons after treatment with γ-secretase modulator
135	Decreased amount of extracellular Aβ 42 by neurons after treatment with γ-secretase modulator
136	Decreased amount of extracellular Aβ 40 by neurons after treatment with γ-secretase modulator
137	Decreased amount of Aβ 42 in neurons after treatment with γ-secretase inhibitor
138	Decreased amount of Aβ 40 in neurons after treatment with γ-secretase inhibitor
139	Decreased amount of Aβ 38 in neurons after treatment with γ-secretase inhibitor
140	Decreased amount of total Aβ in neurons after treatment with γ-secretase inhibitor
141	Increased production of Aβ39 in neurons after treatment with γ-secretase modulator
142	Increased production of Aβ37 in neurons after treatment with γ-secretase modulator
143	Increased generation of mROS in neurons after exposure to low concentrations of valinomycin
144	Decrease production of mROS in neurons in response to valinomycin after treatment with LRRK2 inhibitor GW5074
145	Increase in DA neuron survival in the presence of LRRK2-IN1
146	Decreased susceptibility of neurons to low concentrations of valinomycin induced cell death after treatment with coenzyme Q10
147	Decreased susceptibility of neurons to low concentrations of concanamycin A

	induced cell death after treatment with coenzyme Q10
148	Decreased susceptibility of neurons to valinomycin induced cell death after treatment with LRRK2 inhibitor GW5074
149	Decreased susceptibility of neurons to concanamycin A induced cell death after treatment with LRRK2 inhibitor GW5074
150	Decreased susceptibility of neurons to valinomycin induced cell death after treatment with rapamycin
151	Decreased pERK levels in neurons after treatment with LRRK2-IN1
152	Increased neurite growth rates in neurons after treatment with LRRK2-IN1
153	Decreased neuronal oxidative-stress induced cytotoxicity with MEK kinase inhibitor treatment
154	Increased neurite growth rates after treatment with MEK kinase inhibitor in neurons
155	Decreased degeneration of neurons after treatment with MEK kinase inhibitor
156	Increased levels of TH and cleaved CASPASE3 double positive neurons after treatment with rotenone
157	Increased levels of TH and cleaved CASPASE3 double positive neurons after treatment with 6-OHDA
158	Decreased percent of neurons expressing TH+ after treatment with roscovitine
159	Decrease in sustained calcium ion rise following depolarization of neurons after treatment with nimodipine
160	Increased amount of nuclear gems (SMN protein nuclear foci) in neurons after treatment with valproic acid
161	Increased amount of nuclear gems (SMN protein nuclear foci) in neurons after treatment of tobramycin
162	Increased SMN protein levels in neurons after treatment of valproic acid
163	Increased SMN protein levels in neurons after treatment of tobramycin
164	Decreased susceptibility of neurons to PI3K inhibitor after ectopic progranulin expression
165	Decreased susceptibility to MEK kinase inhibitor after ectopic progranulin experession in neurons
166	Decreased susceptibility to wortmannin after ectopic progranulin expression in neurons
167	Increase in glutamatergic synapse amount in neurons after treatment of IGF1
168	Increased glutamatergic synapse amount and MeCP2 protein levels in neurons after treatment with gentamicin
169	Decreased p-tau/total tau ratio in neurons after treatment with β-secretase inhibitors
170	Decreased anti-glycogen synthase kinase 3 beta (aGSK-3β) levels in neurons after treatment with β-secretase inhibitors
171	Decreased amount of oxidative stress in neurons after treatment with β-secretase inhibitor
172	Increased neuronal susceptibility to oxidative stress when cultured in N2 medium without the supplement B27
173	Increased susceptibility of neurons to valinomycin
174	Increased susceptibility of neurons to MPP+
175	Increased susceptibility of neurons to concanamycin A
176	Increased oxidative stress in neurons after exposure to hydrogen peroxide

177	Increased susceptibility of neurons to 6-OHDA exposure (DA neurons)
178	Increased susceptibility of neurons to MG132 exposure (DA neurons)
179	Increased oxidative stress in neurons
180	Increased susceptibility of neurons to hydrogen peroxide
181	Increased susceptibility to Manganese ethylnebisdithiocarbamate mediated-apoptosis in neurons
182	Increased susceptibility to Paraquat mediated-apoptosis in neurons
183	Increased susceptibility of neurons to rotenone mediated-apoptosis
184	Increased susceptibility of neurons to wortmannin
185	Increased susceptibility of neurons to PI3K inhibitor
186	Increased susceptibility of neurons to MEK kinase inhibitor
187	Increased susceptibility of neurons to ER stress by tunicamycin
188	Increased susceptibility of neurons to proteasome activity inhibition due to lactacystin
189	Increased dopamine-induced oxidative stress in neurons
190	Increased amount of TH-positive neurons after exposure to MG132
191	Decreased amount of dopaminergic neurons after exposure to valinomyacin
192	Decreased amount of dopaminergic neurons after exposure to concanamycin A
193	Accumulation of SNCA in dopaminergic neurons
194	Impairment of mitochondrial translocation of Parkin in neurons after exposure to valinomycin
195	Decreased levels of intracellular progranulin in neurons
196	Decreased levels of secreted progranulin in neurons
197	Increased susceptibility in neurons to staurosporine
198	Increased proportion of neurons expressing upper layer markers (CUX1 and REELIN)
199	Decreased ROS in neurons after treatment with β-secretase inhibitor
200	Impairment of Golgi function in neurons
201	Impairment of ER function in neurons
202	Increased ER stress in neurons
203	Increased susceptibility of neuronal intrinsic aberrant protein aggregation and stress
204	Increased 2',7'- dichoorodihydrofloursecin (DCF) fluorescence in neurons
205	Accumulation of abnormal tubulovesicular structures in neurons
206	Presence of repeat-associated non-ATG (RAN) translation products in neurons
207	Decreased binding of CFT in neurons
208	Decreased levels of SMN protein in neurons
209	Increased amount of protein carbonyls in neurons
210	Increased MAO-A and MAO-B enzymatic activities in neurons
211	Impaired reduction of inner mitochondrial membrane area in neurons after exposure to Carbonyl cyanide m-chlorophenyl hydrazone (CCCP)
212	Decreased secretion levels of Aβ 40 and 42 in neurons after treatment with Dual Antiplatelet Therapy (DAPT)
213	Decreased production of endogenous Aβ 40 in neurons
214	Rescue of PI3k/AKT signaling pathway in neurons after ectopic progranulin expression
215	Rescue of MEK/MAPK signaling pathway in neurons after ectopic progranulin

	expression
216	Decreased levels of glutathione in neurons after exposure to valinomycin
217	Decreased levels of glutathione in neurons after exposure to concanamycin A
218	Decreased levels of glutathione in neurons after exposure to MPP+
219	Decreased levels of glutathione in neurons after exposure to hydrogen peroxide
220	Increased susceptibility to caspase-3 activation in neurons
221	Decreased capacity to generate motor neurons
222	Increased Aβ 38 secretion in neurons
223	Increased Aβ 38/40 ratio in neurons
224	Decreased ratio of secreted APPs-α to APPs-β in neurons
225	Increased generation of APPs-α relative to APPs-β in neurons after treatment with γ-secretase inhibition
226	Increased β-secretase cleavage at APP in neurons
227	Increased colocalization coefficient of APP with endosomal marker EEA1 in neurons
228	Increased levels of phospho-Tau at amino acid S262 in neurons
229	Decreased tau protein levels in neurons after treatment with 3D6 antibody
230	Decreased Tau protein levels in neurons after treatment with AW7 antibody
231	Decrease in size of neurites in neurons
232	Presence of mild degeneration of neuronal processes
233	Presence of AT8-positive p-TAU in predominant and punctate locations in axons of neurons
234	Increased amount of caspase-cleaved TAU fragmentation in neurons
235	Increased susceptibility to MAPT-induced neurotoxicity in DA neurons
236	Increased neurite fragmentation/ degeneration in glutamate neurons
237	Increased neurite fragmentation/ degeneration in GABA neurons
238	Increased amount of aberrant dendritic spines in neurons
239	Decreased amount of synaptic contacts in neurons
240	Presence of constricted/ tapered neurites in neurons
241	Increase in detergent-insoluble TDP-43 in neurons
242	Accumulation of TDP-43 pre-inclusion like aggregates in cytoplasm in neurons
243	Increased amount of SNRPB2 bound to TDP-43 in neurons
244	Increased amount of TDP-43 insoluble fractions in neurons after exposure to arsenite
245	Increase in susceptibility of neurons to death after exposure to arsenite
246	Decreased susceptibility of neurons to arsenite-induced death after exposure to anacardic acid
247	Increased neurite length in neurons after exposure to anacardic acid
248	Decreased amount of TDP-43 insoluble fractions in neurons after exposure to anacardic acid
249	Decreased amount of motor neurons
250	Decreased amount of total cell body volume in neurons
251	Decreased amount of cellular processes from neurons
252	Decreased amount of SMI-32+ neurons at time of late differentiation in neurons
253	Increased percentage of apoptotic neurons
254	Increase activation of initiator procaspase-8 to its cleaved form in neurons

255	Increased amount of cleaved caspase-8 positive neurons
256	Increased amount of membrane bound Fas-ligand in neurons
257	Impairment of the Fas-mediated pathway in motor neurons after exposure to antagonist ZB4 clone of anti-FAS monoclonal antibody (FasNT Ab)
258	Impairment of the Fas-mediated pathway in motor neurons after exposure to caspase-3 specific inhibitor Z-DVED-FMK
259	Increased death of neurons with large CAG expansions
260	Absence of spontaneously firing neurons after two weeks of differentiation
261	Increased risk of neuronal death
262	Increased risk of neuronal death with BDNF withdrawal
263	Increase in caspase-3/7 activity in neurons upon BDNF withdrawal
264	Increased calcium dyshomeostatis in neurons after exposure to pathological glutamate levels
265	Increased susceptibility to 3-methyladenine (3-MA) in neurons
266	Impaired lysosomal system in neurons
267	Decreased amount of autophagic flux in neurons
268	Impaired intracellular calcium homeostasis in neurons
269	Impaired autophagosome-lysosome fusion in neurons
270	Decreased protein levels of Glucocerebrosidase (Gcase) in neurons
271	Decreased enzymatic activity of Glucocerebrosidase (Gcase) in neurons
272	Decreased activity of GBA2 in neurons
273	Decreased activity of β-galactosidase in neurons
274	Accumulation of lysosomes in neurons (as shown by LAMP1 positive particles)
275	Increased intracellular calcium levels in neurons
276	Increased amount of cytosolic calcium induced by caffeine in neurons
277	Increase in RyR-mediated calcium release in neurons
278	Increased susceptibility to ER stress in neurons by calcimycin
279	Increased susceptibility to ER stress in neurons by rotenone
280	Decreased excitability of neurons
281	Presence of severe axonal degeneration in neurons
282	Increased damage to neurons by inhibition of SOH-MEF2C (Sulfenated-myocyte enhancer factor 2C) after exposure to Paraquat
283	Increased damage to neurons by inhibition of SOH-MEF2C (Sulfenated-myocyte enhancer factor 2C) after exposure to Manganese ethylnebisdithiocarbamate
284	Decreased susceptibility to staurosporine after ectopic progranulin expression in neurons
285	Decreased caspase-3 activity in neurons after ectopic progranulin expression
286	Increased mean sodium current densities in neurons
287	Decreased threshold for action potential generation in neurons
288	Increased repetitive firing potential in neurons
289	Presence of spontaneous repetitive firing activity in neurons
290	Increased excitability in neurons
291	Increased amount of depolarized resting membrane potentials (bipolar shaped neurons)
292	Decrease in action potentials in marked amplitude attenuation in GABAergic neurons

293	Decreased amount of action potential firing in GABAergic neurons
294	Decreased output capacity of GABAergic neurons during intense stimulation
295	Increased neuronal death after exposure to 30 minute glutamate pulses
296	Increased apoptotic susceptibility in dopaminergic neurons
297	Increased amount of caspase-3 positive TH neurons after exposure to hydrogen peroxide
298	Decreased size of motor neurons
299	Accumulation of APP-CTFs upon exposure to DAPT in neurons
300	Accumulation of ER-associated degradation substances (Gcase, Nicastrin) in neurons
301	Decreased ratio of post ER-to-ER forms in neurons
302	Increase in levels of nitrative stress in neurons
303	Decreased accumulation of ER-associated degradation substrates (Gcase, Nicastrin) in neurons after treatment with NAB2
304	Decreased levels of ER stress in neurons after treatment with NAB2
305	Decreased levels of nitrative stress in neurons after treatment with NAB2
306	Increased ratio of post ER-to-ER forms in neurons after treatment with NAB2
307	Increased cholesterol accumulation in LE/L compartment in neurons
308	Impaired reduction of nSREBP-2 levels in neurons after exposure to HP-β-cyclodextrin serum
309	Decrease in autophagic clearance in neurons (increased p62 and LC3-II levels)
310	Rescue to normal levels of LC3-II in neurons after treatment with bafilomycin A1
311	Rescue of defective autophagic levels in neurons after treatment with carbamazepine with or without HP-β-cyclodextrin
312	Rescue of defective autophagic levels in neurons after treatment with rapamycin with or without HP-β-cyclodextrin
313	Rescue of defective autophagic levels in neurons after treatment with verapamil with or without HP-β-cyclodextrin
314	Rescue of defective autophagic levels in neurons after treatment with trehalose with or without HP-β-cyclodextrin
315	Increased cell viability in neurons after treatment with carbamazepine with or without HP-β-cyclodextrin
316	Increased cell viability in neurons after treatment with rapamycin with or without HP-β-cyclodextrin
317	Increased cell viability in neurons after treatment with trehalose with or without HP-β-cyclodextrin
318	Increased cell viability in neurons after treatment with verapamil with or without HP-β-cyclodextrin
319	Increased mitochondrial fragmentation along neurites of GABA neurons
320	Decreased mitochondrial membrane potential in medium spiny neurons
321	Increased mROS formation in medium spiny neurons
322	Decreased levels of ATP in medium spiny neurons
323	Decreased mitochondrial fragmentation along neurites in GABA neurons after treatment with dynamin-related protein 1 peptide inhibitor P110-TAT
324	Increased neurite length after treatment with dynamin-related protein 1 peptide inhibitor P110-TAT in neurons
325	Decreased mROS formation in medium spiny neurons after treatment with

	dynamin-related protein 1 peptide inhibitor P110-TAT
326	Increased mitochondrial membrane potential after treatment with dynamin-related protein 1 peptide inhibitor P110-TAT in medium spiny neurons
327	Increased levels of ATP in medium spiny neurons after treatment with dynamin-related protein 1 peptide inhibitor P110-TAT
328	Decreased levels of mitochondrial associated dynamin-related protein 1 after treatment with dynamin-related protein 1 peptide inhibitor P110-TAT in neurons
329	Decreased neuronal death upon BDNF withdrawal after treatment with dynamin-related protein 1 peptide inhibitor P110-TAT in neurons
330	Decreased levels of mitochondrial associated p53 after treatment with dynamin-related protein 1 peptide inhibitor P110-TAT in neurons
331	Decrease in proteolysis of long-lived proteins (α-synuclein, Htt, Tau) in neurons
332	Increased levels of secreted α-synuclein by neurons that can be reuptaken by neighboring neurons
333	Accumulation of neurofilament aggregation in motor neurons
334	Increase in SOD1 aggregates in cytoplasm, nuclei and neurites of motor neurons
335	Absence of neurofilament aggregation-induced mitochondrial swelling in motor neurons
336	Absence of neurofilament aggregation-induced vacuole formation in motor neurons
337	Increased neurite degeneration in motor neurons
338	Recovery of neurofilament subunit proportion (NF-H, NF-M and NF-L) in motor neurons by induction of exogenous NF-L after treatment with Dox (doxycycline)
339	Decreased neurofilament aggregation in motor neurons by induction of exogenous NF-L after treatment with Dox (doxycycline)
340	Decreased neurite degeneration in motor neurons by induction of exogenous NF-L after treatment with Dox (doxycycline)
341	Decreased nuclear size in neurons
342	Decreased glucocerebrosidase enzymatic activity in neurons
343	Accumulation of glucosylsphingolipids in neurons
344	Decrease in dendritic length upon depolarization in neurons
345	Decreased number of terminals in neurons
346	Decreased number of branch points in neurons
347	Decreased neurite complexity in neurons
348	Decreased microtubule stability in neurons
349	Increase in free tubulin in neurons
350	Decrease in polymerized tubulin in pellet fractions in neurons
351	Decreased neurite length in neurons after treatment with colchicine
352	Decreased number of terminals in neurons after treatment with colchicine
353	Decreased number of branch points in neurons after treatment with colchicine
354	Decreased neurite complexity in neurons after treatment with colchicine
355	Increased neurite length after treatment with taxol in neurons
356	Increased number of terminals after treatment with taxol in neurons
357	Increased number of branch points after treatment with taxol in neurons

358	Increased neurite complexity after treatment with taxol in neurons
359	Decreased action potential output in motor neurons
360	Decreased synaptic input in motor neurons
361	Decreased Na+ currents in motor neurons
362	Decreased K+ currents in motor neurons
363	Decreased synaptic activity in motor neurons
364	Increase in early cell death in neurons
365	Increased cell survival in neurons after treatment with curcumin
366	Increased cell survival in neurons after treatment with dantrolene
367	Presence of mis-localized FUS protein in the cytoplasm of motor neurons
368	Accumulation of FUS cytoplasmic aggregates in motor neurons
369	Accumulation of high FUS protein levels within stress granules after treatment with sodium arsenite in neurons
370	Accumulation of high FUS protein levels within stress granules after temperature stress in neurons
371	Increased fragmentation of TAU protein in neurons
372	Increased susceptibility to oxidative stress after treatment with rotenone in neurons
373	Recovery from oxidative stress after treatment with antioxidant coenzyme Q10 in neurons
374	Increased immunoreactivity for AT8 in neurons
375	Recovery from oxidative stress after treatment with GSK-3 inhibitor CHIR99021 in neurons
376	Increased membrane resistance in motor neurons
377	Decrease in Na+ channel inactivation recovery time in motor neurons
378	Increased Na+ current in motor neurons
379	Increased input resistance in neurons
380	Decreased amplitude of spontaneous excitatory post-synaptic currents in neurons
381	Decreased frequency of spontaneous excitatory post-synaptic currents in neurons
382	Impairment of AMPA-mediated synaptic transmission in neurons
383	Impairment of NMDA-mediated synaptic transmission in neurons
384	Decreased amplitude of spontaneous synaptic events in neurons
385	Decreased frequency of spontaneous synaptic events in neurons
386	Decreased current size generation in response to focal application of AMPA in neurons
387	Decreased current size generation in response to focal application of NMDA in neurons
388	Decreased amount of excitatory synapses in neurons
389	Increased amplitude of spontaneous excitatory post-synaptic currents after treatment with IGF1 in neurons
390	Increased frequency of spontaneous excitatory post-synaptic currents after treatment with IGF1 in neurons
391	Decreased input resistance after treatment with IGF1 in neurons
392	Increased current size generation in response to focal application of NMDA after treatment with IGF1 in neurons
393	Restored amplitude of evoked AMPA excitatory post-synaptic currents after treatment with IGF1 in neurons

394	Restored amplitude of evoked NMDA excitatory post-synaptic currents after treatment with IGF1 in neurons
395	Rescue of synaptic transmission after treatment with IGF1 in neurons
396	Increased rate of decay of NMDA-excitatory post-synaptic currents in neurons
397	Restoration of the number of SYN1 HOMER1 puncta after treatment with IGF1 in neurons
398	Increase in TUNEL-positive motor neurons
399	Decreased soma size of motor neurons
400	Decrease in length/number of processes in motor neurons
401	Increased levels of soluble SOD1 in motor neurons after treatmetment with MG132
402	Accumulation of detergent-insoluble SOD1 in motor neurons after treatment with MG132
403	Decreased levels of SDHA protein in motor neurons
404	Decreased levels of MT-COX1 protein in motor neurons
405	Decreased amount of motile mitochonria in motor neurons
406	Increase in mitochondrial density in processes in motor neurons
407	Increased motor neuron survival after treatment with salubrinal
408	Decreased survival of motor neurons when cultured with control glia
409	Presence of abnormal mitochondrial morphology in motor neurons
410	Increased soma size in neurons at one and two weeks post-differentiation
411	Increased total dendritic length in neurons at one and two weeks post-differentiation
412	Decreased frequency of excitatory spontaneous synaptic currents in neurons
413	Decreased density of SV2+ synaptic boutons in neurons
414	Decreased depolarization-induced vesicle release in neurons
415	Decreased velocity of actin movement in axonal growth cones of motor neurons
416	Increased LIMK-1/2 phosphorylation in motor neurons
417	Increased levels of Rac1 in motor neurons
418	Increased cofflin phosphorylation in motor neurons
419	Partial rescue of cell death in neurons after treatment with isoxazole-9
420	Increased neurite-like processes length in neurons
421	Decreased neurite-like processes length in neurons after treatment with isoxazole-9
422	Near complete rescue of cell death in neurons after treatment with isoxazole-9
423	Decreased nuclear:cytoplasmic ratio of endogenous Ran in neurons
424	Increased nuclear:cytoplasmic ratio of endogenous MAP2 in neurons
425	Decreased nuclear:cytoplasmic ratio of endogenous RanGAP1 in neurons
426	Decreased nuclear:cytoplasmic ratio of endogenous NUP62 in neurons
427	Decreased medium spiny neuronal death after treatment with KD3010 (PPAR-δ agonist)
428	Impairment of Akt activity in neurons
429	Rescue of spontaneous excitatory post-synaptic current frequency in neurons after treatment with SC79 (Akt activator)
430	Rescue of spontaneous excitatory post-synaptic current frequency in neurons after treatment with TG003 (Akt activator)

431	Increased levels of exon-10 containing *4R-TAU* isoforms in neurons
432	Increased levels of insoluble P-tau protein in neurons
433	Increased levels of insoluble tau in neurons
434	Increased levels of polyubiquitinated proteins in neurons
435	Increased levels of autophagy markers in neurons
436	Increased levels of CHOP in neurons
437	Decreased cell viability of neurons after exposure to piericidin A
438	Decreased cell viability of neurons after exposure to glutamate
439	Decreased cell viability of neurons after exposure to NMDA
440	Decreased cell viability of neurons after exposure to MG132
441	Decreased cell viability of neurons after exposure to epoxomicin
442	Decreased cell viability of neurons after exposure to Aβ(1–42)
443	Increased cell viability of neurons after treatment with rapamycin
444	Increased cell viability of neurons exposed to rotenone after treatment with rapamycin
445	Increased cell viability of neurons exposed to NMDA after treatment with rapamycin
446	Increased cell viability of neurons exposed to Aβ(1-42) after treatment with rapamycin
447	Accumulation of cholesterol in cell bodies of neurons
448	Accumulation of cholesterol in cellular extensions of neurons
449	Increased co-localization of cholesterol/ GM2 in neurons
450	Increased accumulation of osmiophilic material in neurons
451	Increased levels of GM2 in neurons
452	Decreased levels of GM3 in neurons
453	Increased levels of Hex A mRNA in neurons
454	Decreased Hex A enzymatic activity in neurons
455	Increased protein levels of Hex A in neurons
456	Increased levels of nuclear, insoluble of hnRNP A2/B1in motor neurons
457	Decreased SRSF7 levels in motor neurons after treatment with an ASO targeting *Hnrnpa2b1*
458	Accumulation of hnRNP A2/B1+ stress granules in motor neurons after exposure to puromycin
459	Increased neurite outgrowth in GABAergic medium spiny neurons
460	Increased neurite ramification in GABAergic medium spiny neurons
461	Increased Na+ current amplitudes in medium spiny neurons
462	Decreased voltage dependence of Na+ channel activation in medium spiny neurons
463	Increased amplitude of provoked action potentials in medium spiny neurons
464	Increased amplitude of spontaneous action potentials in medium spiny neurons
465	Increased amplitude of miniature postsynaptic currents in medium spiny neurons
466	Increased percentage of medium spiny neurons showing miniature postsynaptic currents
467	Decreased frequency of synaptic currents in medium spiny neurons after treatment with phallacadin
468	Decreased amplitude of synaptic currents in medium spiny neurons after treatment with phallacadin

469	Decreased amplitude of action potentials in medium spiny neurons after treatment with phallacadin
470	Decreased frequency of synaptic currents in medium spiny neurons after treatment with PP2 (Src kinase inhibitor)
471	Decreased amplitude of synaptic currents in medium spiny neurons after treatment with PP2 (Src kinase inhibitor)
472	Decreased amplitude of action potentials in medium spiny neurons after treatment with PP2 (Src kinase inhibitor)
473	Decreased number of medium spiny neurons at time of late differentiation
474	Increased G/F-actin ration in medium spiny neurons
475	Increased phosphorylation of cofflin in medium spiny neurons
476	Decreased intracellular levels of dopamine in dopaminergic neurons
477	Decreased dopamine reuptake in dopaminergic neurons
478	Decreased protein levels of Glucocerebrosidase (Gcase) in dopaminergic neurons
479	Decreased levels of DAT1 mRNA in dopaminergic neurons
480	Decreased levels of VMAT2 mRNA in dopaminergic neurons
481	Decreased activity levels of Glucocerebrosidase (Gcase) in dopaminergic neurons
482	Increased protein levels of Glucocerebrosidase (Gcase) in dopaminergic neurons after treatment with NCGC607
483	Increased activity levels of Glucocerebrosidase (Gcase) in dopaminergic neurons after treatment with NCGC607
484	Increased translocation of Glucocerebrosidase (Gcase) to the lysosome in dopaminergic neurons after treatment with NCGC607
485	Increased levels of lysosomal GlcSph in dopaminergic neurons
486	Decreased levels of lysosomal GlcSph in dopaminergic neurons after treatment with NCGC607
487	Presence of α-synuclein in soma of dopaminergic neurons
488	Decreased levels of α-synuclein in dopaminergic neurons after treatment with NCGC607
489	Presence of co-localized α-synuclein/Lamp2 in dopaminergic neurons
490	Decreased levels of colocalized α-synuclein/Lamp2 in dopaminergic neurons after treatment with NCGC607
491	Decreased average maximal firing rate in neurons
492	Presence of abnormal firing activity in neurons after injection of suprathreshold current steps
493	Decreased peak amplitudes of voltage-dependent Na+ currents in neurons
494	Presence of abnormal mitochondrial morphology (swollen, damaged cristae) in neurons
495	Increased proportion of altered mitochondria in neurons
496	Decreased mitochondrial membrane potential in neurons
497	Decreased glutathione levels in neurons
498	Decreased aconitase activity in neurons
499	Increased TfR1 levels in neurons
500	Decreased ferritin levels in neurons
501	Decreased loss of neurons after treatment with CoA
502	Increased mature firing rate of neurons after treatment with CoA
503	Decreased ROS levels in neurons after treatment with CoA

504	Decreased synaptic density in neurons
505	Decreased action potential spike number in neurons
506	Decreased number of synchronized events in neurons
507	Increased nuclear disruption in NSCs (enlarged nuclear area, decrease in circularity, and loss of lamin B1 and B2 on specific folds of nuclear envelope)
508	Accumulation of intranuclear RNA foci in NSCs
509	Decreased clonal expansion in NSCs
510	Increased proteasomal stress in NSCs
511	Presence of spontaneous NSC differentiation at late passages
512	Impaired induction of IFN-β and IFN-γ by NSCs after exposure to polyinosinic:polycytidylic acid
513	Decrease in average Oxygen Consumption Rate (OCR) in NSCs after exposure to Carbonyl cyanide-p-trifluoromethoxypheny (FCCP)
514	Increase in caspase-3/7 activity in NSCs upon growth factor deprivation
515	Increased susceptibility of NSCs to death after growth factor withdrawal
516	Increase in TUNEL-positive NSCs
517	Increased apoptosis in NSCs after exposure to MG132
518	Partial rescue of normal nuclear morphology in NSCs after treatment with PI3K inhibitor
519	Rescue of aberrant cellular parameters in NSCs after treatment with LRRK2-In-1
520	Decreased phosphorylation of LRRK2 downstream targets in NSCs after treatment with LRRK2-In-1
521	Decreased protein levels of N-cadherin in NSCs
522	Decreased binding of phalloidin-peptide to actin cytoskeleton in NSCs
523	Decreased cell-cell adhesion properties in NSCs
524	Decreased intracellular ATP (energy metabolism compromised) in NSCs
525	Decreased ATP/ADP ratios (energy metabolism compromisation) in NSCs
526	Impaired reductions in HSV-1-GFP replication levels in NSCs after treatment with polyinosinic:polycytidylic acid
527	Decreased TGF-β1 mRNA in NSCs
528	Increase in sustained calcium ion rise following depolarization of NSCs
529	Decrease in sustained calcium ion rise following depolarization of NSCs after treatment with nimodipine
530	Increased amount of centromeric signals accompanied by reorganization of centromeric heterochromatin in NSCs
531	Increased phosphorylation of lamin B1 and B2 in NSCs
532	Impaired formation of MAP2+ NSCs
533	Presence of aberrant non-neuronal cellular morphology in NSCs
534	Impaired ability to retain clonogenic and differentiation capacity in NSCs
535	Decreased amount of TUJ1+ NSCs
536	Increased levels of mitochondrial DNA damage in NSCs
537	Increased Aβ42/40 ratio in NSCs
538	Impaired structure of adherens junctions in NSCs
539	Decreased amount of Aβ42 in NSCs after treatment with γ-secretase modulator
540	Decreased amount of Aβ40 in NSCs after treatment with γ-secretase modulator
541	Decreased amount of Aβ38 in NSCs after treatment with γ-secretase

	modulator
542	Decreased Aβ42:Aβ40 ratio in NSCs after treatment with γ-secretase modulator
543	Decreased protein levels of Glucocerebrosidase (Gcase) in NSCs
544	Decreased activity levels of Glucocerebrosidase (Gcase) in NSCs
545	Decreased heme levels in NSCs
546	Increased electron clear vacuoles under basal conditions in cytoplasm of astrocytes
547	Accumulated Aβ oligomers in astrocytes
548	Decreased amount of Aβ oligomers in astrocytes after treatment with β-secretase inhibitor
549	Increased binding protein (BiP) levels in astrocytes
550	Increased ROS levels in basal conditions in astrocytes
551	Increased levels of cleaved caspase-4 in astrocytes
552	Increased ER stress in astrocytes
553	Increased oxidative stress in astrocytes
554	Decreased levels of binding protein (BiP) in astrocytes after treatment with β-secretase inhibitor
555	Decreased levels of cleaved caspase-4 in astrocytes after treatment with β-secretase inhibitor
556	Increased risk of death in astrocytes under basal conditions
557	Presence of mislocalization of TDP-43 protein in cytoplasm of astrocytes
558	Decreased levels of SMN protein in astrocytes
559	Absence of nuclear gems (SMN protein nuclear foci) in astrocytes
560	Accumulation of intranuclear RNA foci in astrocytes
561	Impaired induction of IFN-β and IFN-γ by astrocytes after exposure to polyinosinic:polycytidylic acid
562	Increased amounts of nuclear gems (SMN protein nuclear foci) in astrocytes after treatment with valproic acid
563	Increased amounts of nuclear gems (SMN protein nuclear foci) in astrocytes after treatment with tobramycin
564	Increased SMN protein levels in astrocytes after treatment with valproic acid
565	Increased levels of SMN protein levels in astrocytes after treatment with tobramycin
566	Decrease in ROS in astrocytes after treatment with β-secretase inhibitor
567	Accumulation of soluble TDP-43 in astrocytes
568	Increased levels of exon-10 containing *4R-TAU* isoforms in astrocytes
569	Increased astrocyte size
570	Decreased nucleus/cytoplasm ratio in astrocytes
571	Increased levels of ubiquinated proteins in astrocytes
572	Increased susceptibility to oxidative stress in astrocytes
573	Increased cell death in astrocytes after exposure to rotenone
574	Increased release of lactate dehydrogenase in astrocytes after exposure to rotenone
575	Increased protein levels of ANXA2 in astrocytes
576	Increased susceptibility of oligodendrocytes to HSV-1 infection
577	Decreased susceptibility of oligodendrocytes to HSV-1 infection after treatment with exogenous IFN-α/ β
578	Impaired induction of IFN-β and IFN-γ by oligodendrocytes after exposure to

	polyinosinic:polycytidylic acid
579	Impaired induction of IFN-β and IFN-γ by oligodendrocytes following HSV-1 infection
580	Increased replication speed of HSV-1-GFP in oligodendrocytes
581	Rescued replication speed of HSV-1-GFP in oligodendrocytes after treatment with IFN-α2b
582	Rescued replication speed of HSV-1-GFP in oligodendrocytes after treatment with IFN-β
583	Increased very long chain fatty acid (C26:0/C22:0 ratio) levels in oligodendrocytes
584	Increased very long chain fatty acid levels in oligodendrocytes compared to AMN ABCD1 (X-linked Adrenoleukodystrophy) mutations
585	Decreased C26:0/C22:0 ratio levels in oligodendrocytes by lovastatin (through induced upregulation of ABCD2 gene)
586	Decreased C26:0/C22:0 ratio levels in oligodendrocytes after treatment with 4-PBA (through induced upregulation of ABCD2 gene)
587	Presence of mislocalization of PLP1 protein to the ER in oligodendrocytes
588	Increased susceptibility to ER stress induced by exposure to tunicamycin in oligodendrocytes
589	Increased amount of apoptotic cells in oligodendrocytes
590	Decreased frequency of myelin formation in oligodendrocytes
591	Decreased thickness of myelin sheaths in oligodendrocytes
592	Presence of aberrant ER morphologies in oligodendrocytes
593	Impaired induction of MX1 (IFN-inducible molecule) by oligodendrocytes following HSV-1 infection
594	Impaired induction of NF-κB1 and MX1 (IFN-inducible molecule) by oligodendrocytes after exposure to polyinosinic:polycytidylic acid
595	Presence of mislocalization of PLP1 protein in the perinuclear cytoplasm in oligodendrocytes
596	Presence of colocalization of PLP1 protein with KDEL in oligodendrocytes
597	Increased susceptibility to ER stress in oligodendrocytes
598	Presence of scatter O4 staining in the processes of oligodendrocytes
599	Increased nuclear condensation in apoptotic oligodendrocytes
600	Presence of increased ER intermembrane space diation in oligodendrocytes
601	Decreased expression of cytoskeleton-associated proteins in iPSCs
602	Increased expression of MSH2 enzyme in iPSCs
603	Decreased neuronal differentiation in iPSCs
604	Presence of premature senescence phenotypes in iPSCs
605	Increased GAA-TTC repeat instability in iPSCs
606	Increased susceptibility to oxidative stress in iPSCs
607	Increased apoptotic cell death in iPSCs through activation of the p53-mediated apoptotic pathway
608	Increased amount of peroxiredoxin1, peroxiredoxin2 and peroxiredoxin6 in iPSCs
609	Decreased formation of TuJ+ iPSCs
610	Absence of progerin (truncated splicing mutant of Lamin A) in iPSCs
611	Accumulation of intranuclear RNA foci in iPSCs
612	Decreased rosette forming efficiency in iPSCs
613	Increased iPSC death in absence of CSB

614	Increased levels of ROS in iPSCs
615	Decreased growth rate in iPSCs
616	Increased instability of GAA repeats in iPSCs
617	Decreased frataxin levels in iPSCs
618	Presence of delayed development of electrophysiological functionality in iPSCs
619	Decreased mitochondrial membrane potential in iPSCs
620	Increased susceptibility of iPSCs to form huntingtin aggregates after exposure to MG132
621	Decreased levels of representative antioxidant molecules (Superoxide dismutase1, Glutathiomine transferase, and Glutathione peroxidase 1) in iPSCs
622	Increased amount of TUNEL-positive iPSCs
623	Increased expression of BTF3 and ATM in iPSCs
624	Presence of an aberrant switch of E-cadherin to N-cadherin in iPSCs
625	Presence of abnormal neural rosette during early differentiation in iPSCs
626	Impaired structural integrity in the cell to cell junction (membrane recruitment/microdetachment) in iPSCs
627	Decreased expression of ATP7A in iPSCs
628	Increased distribution of ATP7A molecules throughout cytoplasm in iPSCs
629	Presence of an abnormal reticular distribution of ATP7A in iPSCs
630	Increased opacity/density embryoid body structures in iPSCs
631	Presence of abnormal epithelial morphology of attached cells from embryoid bodies in iPSCs
632	Impaired gradual decrease of E-cadherin in iPSC derivatives
633	Decreased expression of NESTIN and aberrant rosette lumens during neurosphere development in iPSCs
634	Decreased number of N-cad+/ Sox2+ neural rosettes in iPSCs
635	Absence of differentiation into TuJ1- expressing neurites in iPSCs
636	Absence of differentiation into MAP2- expressing neurites in iPSCs
637	Increased positive staining for Periodic acid-Schiff in iPSCs
638	Accumulation of glycogen granules in cytoplasm in iPSCs
639	Decreased amount of glycogen granules after treatment with acid-α-glucosidase in iPSCs
640	Decreased amount of Aβ42 in iPSCs after treatment with γ-secretase modulator
641	Decreased amount of Aβ40 in iPSCs after treatment with γ-secretase modulator
642	Decreased amount of Aβ38 in iPSCs after treatment with γ-secretase modulator
643	Decreased Aβ42:Aβ40 ratio in iPSCs after treatment with γ-secretase modulator
644	Accumulation of high FUS protein levels within stress granules after treatment with sodium arsenite in iPSCs
645	Accumulation of high FUS protein levels within stress granules after temperature stress in iPSCs
646	Increased punctuate cytoplasmic delocalization of FUS protein in iPSCs
647	Accumulation of high FUS protein levels within stress granules in iPSCs after treatment with sorbitol

648	Absence of random X-inactivation in iPSCs
649	Increased levels of mitochondrial associated dynamin-related protein 1 in iPSCs
650	Increased levels of mitochondrial associated p53 in iPSCs
651	Decreased levels of mitochondrial associated dynamin-related protein 1 after treatment with dynamin-related protein 1 peptide inhibitor P110-TAT in iPSCs
652	Decreased levels of mitochondrial associated p53 after treatment with dynamin-related protein 1 peptide inhibitor P110-TAT in iPSCs
653	Decreased production of neurons in iPSCs
654	Increased 7-, 8- DHC levels in iPSCs after exposure to cholesterol-deficient mTesR1 medium
655	Decreased cholesterol levels in iPSCs after exposure to cholesterol-deficient mTesR1 medium
656	Decreased rosette formation in iPSCs
657	Increased rates of neuronal differentiation in iPSCs
658	Increased gap and tight junctions in iPSCs cultured in cholesterol-deficient mTesR1 medium after treatment with LDL
659	Decreased formation of secondary filaments in iPSCs cultured in cholesterol-deficient mTesR1 medium after treatment with LDL
660	Increased formation of rosette structures in iPSCs iPSCs cultured in cholesterol-deficient mTesR1 medium after treatment with LDL
661	Presence of poorly defined SOX2+/ PAX6+ neural rosette structures in iPSCs
662	Presence of long neuronal projections in iPSCs after extended differentiation
663	Presence of spindled neural progenitor-like morphology in iPSCs when cultured in cholesterol-deficient mTesR1 medium

Appendix Table S5. **Phenotypes by Category with their Network Color**		
Phenotype Identifier Range	Phenotypic Category	Color in Network
1-25	Rescue/Recovery from Disease Phenotypes After Chemical Treatment	Light Purple
26-34	Absence of Expected Normal Phenotypes	Red
35-65	Accumulation of Molecules	Orange
66-103	Presence of Abnormal Cellular Structures	Dark Blue-Violet
104-138	Impairment of Expected Cellular Functions	Pink
139-150	Decreased Susceptibility to Chemical Exposure	Light Green
151-184	Increased Susceptibility to Chemical Exposure	Cyan
185-416	Increased Cellular Processes and Products	Emerald-Green
417-663	Decreased Cellular Processes and Products	Burnt Yellow

Appendix Table S6. **Overlapping Neuronal Phenotypes**

Rescued replication speed of HSV-1-GFP in neurons after treatment with IFN-α2b	Childhood HSE, *UNC-93-B*, Not Specified<br>Childhood HSE, *TLR3*, Not Specified
Rescued replication speed of HSV-1-GFP in neurons after treatment with IFN-β	Childhood HSE, *UNC-93-B*, Not Specified<br>Childhood HSE, *TLR3*, Not Specified
Accumulation of monomeric α-synuclein in neurons	Gaucher's Disease, *GBA1*, Not Specified<br>Parkinson's Disease, *Parkin*, Not Specified<br>Parkinson's Disease, *SNCA*, Triplication<br>Parkinson's Disease, *LRRK2*, G2019S
Accumulation of Aβ oligomers in neurons	Alzheimer's Disease, *APP*, E693Δ<br>Alzheimer's Disease, Sporadic, Not Specified
Accumulation of intranuclear RNA foci in neurons	Frontotemporal Dementia/Amyotrophic Lateral Sclerosis, *C9ORF72*, GGGGCC Repeats<br>Dystrophia Myotonica type 1, *DMPK*, Not Specified
Accumulation of SNCA in dopaminergic neurons	Parkinson's Disease, *LRRK2*, G2019S<br>Parkinson's Disease, *SNCA*, A53T
Impaired neuronal network connectivity in glutamatergic neurons	Rett's Syndrome, *MeCP2*, Q244X<br>Pelizaeus-Merzbacher Disease, *MAPT*, Missense
Decreased susceptibility of neurons to HSV-1 infection after treatment with exogenous IFN-a/ β	Childhood HSE, *UNC-93*-B, Not Specified<br>Childhood HSE, *TLR3*, Not Specified
Decreased susceptibility of neurons to low concentrations of valinomycin induced cell death after treatment with coenzyme Q10	Parkinson's Disease, *PINK1*, Q456X<br>Parkinson's Disease, *LRRK2*, R1441C<br>Parkinson's Disease, *LRRK2*, G2019S
Decreased susceptibility of neurons to low concentrations of concanamycin A induced cell death after treatment with coenzyme Q10	Parkinson's Disease, *PINK1*, Q456X<br>Parkinson's Disease, *LRRK2*, R1441C<br>Parkinson's Disease, *LRRK2*, G2019S
Decreased susceptibility of neurons to valinomycin induced cell death after treatment with LRRK2 inhibitor GW5074	Parkinson's Disease, *PINK1*, Q456X<br>Parkinson's Disease, *LRRK2*, R1441C<br>Parkinson's Disease, *LRRK2*, G2019S
Increased susceptibility of	Childhood HSE, *UNC-93-B*, Not Specified

neurons to HSV-1 infection	Childhood HSE, *TLR3*, Not Specified
Increased susceptibility of neurons to valinomycin	Parkinson's Disease, *PINK1*, Q456X<br>Parkinson's Disease, *LRRK2*, R1441C<br>Parkinson's Disease, *LRRK2*, G2019S
Increased susceptibility of neurons to MPP+	Parkinson's Disease, *PINK1*, Q456X<br>Parkinson's Disease, *LRRK2*, R1441C<br>Parkinson's Disease, *LRRK2*, G2019S
Increased susceptibility of neurons to concanamycin A	Parkinson's Disease, *PINK1*, Q456X<br>Parkinson's Disease, *LRRK2*, R1441C<br>Parkinson's Disease, *LRRK2*, G2019S
Increased susceptibility of neurons to MG132 exposure (DA neurons)	Parkinson's Disease, *PINK1*, Q456X<br>Parkinson's Disease, *LRRK2*, R1441C<br>Parkinson's Disease, *LRRK2*, G2019S
Increased susceptibility of neurons to hydrogen peroxide	Huntington's Disease, *HTT*, CAG Repeats<br>Parkinson's Disease, *PINK1*, Q456X<br>Parkinson's Disease, *SNCA*, Triplication
Increased susceptibility of neurons to rotenone mediated-apoptosis	Parkinson's Disease, *SNCA*, A53T<br>Frontotemporal Dementia, *MAPT*, N279K<br>Frontotemporal Dementia, *MAPT*, V337M<br>Frontotemporal Dementia, *MAPT*, A152T
Increased amount of autophagosomes in neurons	Parkinson's Disease, *GBA1*, Not Specified<br>Parkinson's Disease, *LRRK2*, G2019S<br>Niemann- Pick Type C, *NPC1*, I1061T
Increased percentage of neurons containing cytoplasmic TDP-43	Frontotemporal Dementia, *PGRN*, S116X<br>Amyotrophic Lateral Sclerosis, *TDP-43*, Q343R<br>Amyotrophic Lateral Sclerosis, *TDP-43*, M337V
Increased Aβ42/40 ratio in neurons	Alzheimer's Disease, *APP*, V171L<br>Alzheimer's Disease, *APP*, V171I<br>Alzheimer's Disease, *PSEN1*, L166P<br>Alzheimer's Disease, *PSEN2*, N141I<br>Alzheimer's Disease, *PSEN1*, M146L<br>Alzheimer's Disease, *PSEN1*, A246E<br>Alzheimer's Disease, *PSEN1*, H163R
Increased extracellular Aβ42 in neurons	Alzheimer's Disease, *PSEN1*, L166P<br>Alzheimer's Disease, *PSEN2*, N141I<br>Alzheimer's Disease, *APP*, V171L

Increased positive staining for cleaved caspase-3 in neurons	Parkinson's Disease, *LRRK2*, G2019S<br>Spinal Muscular Atrophy, *SMN1*, Not Specified
Increased levels of TAU protein in neurons	Alzheimer's Disease, *APP*, V171I<br>Parkinson's Disease, *LRRK2*, G2019S<br>Frontotemporal dementia, *MAPT*, A152T
Increase in very long chain fatty acid (C26:0/C22:0 ratio) levels in neurons	X-Linked Adrenoleukodystrophy, *ABCD1*/ *ALDP*, AMN Patient<br>X-Linked Adrenoleukodystrophy, *ABCD1*/ *ALDP*, CCALD Patient
Increased oxidative stress in neurons	Alzheimer's Disease, *APP*, E693Δ<br>Alzheimer's Disease, Sporadic, Not Specified<br>Parkinson's Disease, *LRRK2*, G2019S<br>Parkinson's Disease, *SNCA*, Triplication<br>Parkinson's Disease, *Parkin*, Not Specified<br>Amyotrophic Lateral Sclerosis, *TDP-43*, M337V<br>Amyotrophic Lateral Sclerosis, *TDP-43*, Q343R<br>Parkinson's Disease, *PINK1*, Q456X<br>Frontotemporal Dementia, *MAPT*, A152T
Increased ER stress in neurons	Alzheimer's Disease, *APP*, E693Δ<br>Parkinson's Disease, *SNCA*, Triplication<br>Frontotemporal Dementia, *MAPT*, V337M<br>Frontotemporal Dementia, *MAPT*, N279K<br>Alzheimer's Disease, Sporadic, Not Specified
Increased risk of neuronal death	Niemann-Pick Type C, *NPC1*, I1061T<br>Huntington's Disease, *HTT*, CAG Repeats<br>Parkinson's Disease, *LRRK2*, G2019S<br>Pantothenate kinase-associated neurodegeneration, *PANK2*, c.1259delG
Increased levels of binding protein (BiP) in neurons	Alzheimer's Disease, *APP*, E693Δ<br>Alzheimer's Disease, Sporadic, Not Specified<br>Frontotemporal Dementia, *MAPT*, A152T
Increased oxidative stress in neurons after exposure to hydrogen peroxide	Alzheimer's Disease, *APP*, E693Δ<br>Parkinson's Disease, *LRRK2*, G2019S
Increased ROS levels in basal conditions in neurons	Parkinson's Disease, *SNCA*, A53T<br>Alzheimer's Disease, *APP*, E693Δ<br>Alzheimer's Disease, Sporadic, Not Specified<br>Pantothenate kinase-associated neurodegeneration, *PANK2*, c.1259delG<br>Pantothenate kinase-associated neurodegeneration, *PANK2*, c.569insA
Increased excitability in neurons	Dravet Syndrome, *SCN1A*, IVS14+3A>T

	Amyotrophic Lateral Sclerosis, *C9ORF72*, GGGGCC Repeats<br>Amyotrophic Lateral Sclerosis, *TDP-43*, M337V<br>Spinal Muscular Atrophy, *SMN1*, Deletions of exons 7 and 8
Increased replication speed of HSV-1-GFP in neurons	Childhood HSE, *UNC-93-B*, Not Specified<br>Childhood HSE, *TLR3*, Not Specified
Decreased amount of extracellular Aβ 42 by neurons by treatment with a selective Aβ42-lowering agent	Alzheimer's Disease, *PSEN1*, A246E<br>Alzheimer's Disease, *PSEN2*, N141I
Decreased amount of extracellular Aβ 42 by neurons after treatment with γ-secretase inhibitor	Alzheimer's Disease, *PSEN1*, A246E<br>Alzheimer's Disease, *PSEN2*, N141I
Decreased amount of dopaminergic neurons after exposure to valinomycin	Parkinson's Disease, *LRRK2*, R1441C<br>Parkinson's Disease, *PINK1* Q456X
Decreased amount of dopaminergic neurons after exposure to concanamycin A	Parkinson's Disease, *LRRK2*, R1441C<br>Parkinson's Disease, *PINK1*, Q456X
Decreased ROS in neurons after treatment with β-secretase inhibitor	Alzheimer's Disease, *APP*, E693Δ<br>Alzheimer's Disease, Sporadic, Not Specified
Decrease in size of neurites	Amyotrophic Lateral Sclerosis, *TDP-43*, Q343R<br>Amyotrophic Lateral Sclerosis, *TDP-43*, G298S<br>Amyotrophic Lateral Sclerosis, *TDP-43*, M337V<br>Parkinson's Disease, *Parkin*, Deletions on exons 7 and 8<br>Frontotemporal Dementia, *MAPT*, V337M<br>Frontotemporal Dementia, *MAPT*, N279K<br>Tauopathies, *MAPT*, A152T
Decreased amount of Aβ-oligomers in neurons with β-secretase inhibitor treatment	Alzheimer's Disease, *APP*, E693Δ<br>Alzheimer's Disease, Sporadic, Not Specified
Decreased motor neuron survival	Spinal Muscular Atrophy, *SMN1*, Deletions on exon 7 and 8<br>Amyotrophic Lateral Sclerosis, *SOD1*, A4V<br>Multisystem proteinopathy, *hRNPA2B1,* D290V
Decreased neurite outgrowth in neurons	Huntington's Disease, *HTT*, CAG Repeats<br>Spinal Muscular Atrophy, *SMN1*, Not Specified<br>Parkinson's Disease, *LRRK2*, G2019S
Decreased neurite complexity in	Frontotemporal Dementia, *MAPT*, N279K

neurons	Frontotemporal Dementia, *MAPT*, V337M<br>Parkinson's Disease, *Parkin*, Deletions on exon 7 and 8
Decreased action potential output in motor neurons	Amyotrophic Lateral Sclerosis, *C9ORF72*, GGGGCC Repeats<br>Amyotrophic Lateral Sclerosis, *TDP-43*, M337V
Decreased synaptic input in motor neurons	Amyotrophic Lateral Sclerosis, *C9ORF72*, GGGGCC Repeats<br>Amyotrophic Lateral Sclerosis, *TDP-43*, M337V
Decrease Na+ currents in motor neurons	Amyotrophic Lateral Sclerosis, *C9ORF72*, GGGGCC Repeats<br>Amyotrophic Lateral Sclerosis, *TDP-43*, M337V
Decreased K+ currents in motor neurons	Amyotrophic Lateral Sclerosis, *C9ORF72*, GGGGCC Repeats<br>Amyotrophic Lateral Sclerosis, TDP-43, M337V
Decreased synaptic activity in motor neurons	Amyotrophic Lateral Sclerosis, *C9ORF72*, GGGGCC Repeats<br>Amyotrophic Lateral Sclerosis, *TDP-43*, M337V
Decreased levels of binding protein (BiP) in neurons after treatment with DHA	Alzheimer's Disease, *APP*, E693Δ<br>Alzheimer's Disease, Sporadic, Not Specified
Decreased levels of binding protein (BiP) in neurons after treatment with β-secretase inhibitor	Alzheimer's Disease, *APP*, E693Δ<br>Alzheimer's Disease, Sporadic, Not Specified
Decreased amount of motor neurons	Amyotrophic Lateral Sclerosis, *SOD1*, A4V<br>Spinal Muscular Atrophy, *SMN1*, Not Specified
Increased levels of nuclear, insoluble of hnRNP A2/B1in motor neurons	Multisystem proteinopathy, *hRNPA2B1,* D290V<br>Multisystem proteinopathy, *VCP,* R155H
Decreased SRSF7 levels in motor neurons after treatment with an ASO targeting Hnrnpa2b1	Multisystem proteinopathy, *hRNPA2B1,* D290V<br>Multisystem proteinopathy, *VCP,* R155H
Accumulation of hnRNP A2/B1+ stress granules in motor neurons after exposure to puromycin	Multisystem proteinopathy, *hRNPA2B1,* D290V<br>Multisystem proteinopathy, *VCP,* R155H
Decreased Basal Oxygen Consumption Rate (OCR) in neurons	*PANK2*, c.1259delG<br>Pantothenate kinase-associated neurodegeneration, *PANK2*, c.569insA<br>Parkinson's Disease, *LRRK2*, G2019S<br>Parkinson's Disease, *LRRK2*, R1441C
Increased neurite outgrowth in GABAergic medium spiny neurons	Chorea-Acanthocytosis, *VPS13A,* c.7806G>A<br>Chorea-Acanthocytosis, *VPS13A,* c.4282G>C

Increased neurite ramification in GABAergic medium spiny neurons	Chorea-Acanthocytosis, *VPS13A,* c.7806G>A<br>Chorea-Acanthocytosis, *VPS13A,* c.4282G>C
Increased Na+ current amplitudes in medium spiny neurons	Chorea-Acanthocytosis, *VPS13A,* c.7806G>A<br>Chorea-Acanthocytosis, *VPS13A,* c.4282G>C
Decreased voltage dependence of Na+ channel activation in medium spiny neurons	Chorea-Acanthocytosis, *VPS13A,* c.7806G>A<br>Chorea-Acanthocytosis, *VPS13A,* c.4282G>C
Increased amplitude of provoked action potentials in medium spiny neurons	Chorea-Acanthocytosis, *VPS13A,* c.7806G>A<br>Chorea-Acanthocytosis, *VPS13A,* c.4282G>C
Increased amplitude of spontaneous action potentials in medium spiny neurons	Chorea-Acanthocytosis, *VPS13A,* c.7806G>A<br>Chorea-Acanthocytosis, *VPS13A,* c.4282G>C
Increased amplitude of miniature postsynaptic currents in medium spiny neurons	Chorea-Acanthocytosis, *VPS13A,* c.7806G>A<br>Chorea-Acanthocytosis, *VPS13A,* c.4282G>C
Increased percentage of medium spiny neurons showing miniature postsynaptic currents	Chorea-Acanthocytosis, *VPS13A,* c.7806G>A<br>Chorea-Acanthocytosis, *VPS13A,* c.4282G>C
Decreased frequency of synaptic currents in medium spiny neurons after treatment with phallacadin	Chorea-Acanthocytosis, *VPS13A,* c.7806G>A<br>Chorea-Acanthocytosis, *VPS13A,* c.4282G>C
Decreased amplitude of synaptic currents in medium spiny neurons after treatment with phallacadin	Chorea-Acanthocytosis, *VPS13A,* c.7806G>A<br>Chorea-Acanthocytosis, *VPS13A,* c.4282G>C
Decreased amplitude of action potentials in medium spiny neurons after treatment with phallacadin	Chorea-Acanthocytosis, *VPS13A,* c.7806G>A<br>Chorea-Acanthocytosis, *VPS13A,* c.4282G>C
Decreased frequency of synaptic currents in medium spiny neurons after treatment with PP2 (Src kinase inhibitor)	Chorea-Acanthocytosis, *VPS13A,* c.7806G>A<br>Chorea-Acanthocytosis, *VPS13A,* c.4282G>C
Decreased amplitude of synaptic currents in medium spiny neurons after treatment with PP2 (Src kinase inhibitor)	Chorea-Acanthocytosis, *VPS13A,* c.7806G>A<br>Chorea-Acanthocytosis, *VPS13A,* c.4282G>C
Decreased amplitude of action potentials in medium spiny neurons after treatment with PP2 (Src kinase inhibitor)	Chorea-Acanthocytosis, *VPS13A,* c.7806G>A<br>Chorea-Acanthocytosis, *VPS13A,* c.4282G>C
Decreased number of medium spiny neurons at time of late differentiation	Chorea-Acanthocytosis, *VPS13A,* c.7806G>A<br>Chorea-Acanthocytosis, *VPS13A,* c.4282G>C
Increased G/F-actin ration in medium spiny neurons	Chorea-Acanthocytosis, *VPS13A,* c.7806G>A<br>Chorea-Acanthocytosis, *VPS13A,* c.4282G>C

Increased phosphorylation of cofflin in medium spiny neurons	Chorea-Acanthocytosis, *VPS13A,* c.7806G>A<br>Chorea-Acanthocytosis, *VPS13A,* c.4282G>C
Decreased average maximal firing rate in neurons	Pantothenate kinase-associated neurodegeneration, *PANK2*, c.1259delG<br>Pantothenate kinase-associated neurodegeneration, *PANK2*, c.569insA
Presence of abnormal firing activity in neurons after injection of suprathreshold current steps	Pantothenate kinase-associated neurodegeneration, *PANK2*, c.1259delG<br>Pantothenate kinase-associated neurodegeneration, *PANK2*, c.569insA
Decreased peak amplitudes of voltage-dependent Na+ currents in neurons	Pantothenate kinase-associated neurodegeneration, *PANK2*, c.1259delG<br>Pantothenate kinase-associated neurodegeneration, *PANK2*, c.569insA
Presence of abnormal mitochondrial morphology (swollen, damaged cristae) in neurons	Pantothenate kinase-associated neurodegeneration, *PANK2*, c.1259delG<br>Pantothenate kinase-associated neurodegeneration, *PANK2*, c.569insA
Increased proportion of altered mitochondria in neurons	Pantothenate kinase-associated neurodegeneration, *PANK2*, c.1259delG<br>Pantothenate kinase-associated neurodegeneration, *PANK2*, c.569insA
Decreased mitochondrial membrane potential in neurons	Pantothenate kinase-associated neurodegeneration, *PANK2*, c.1259delG<br>Pantothenate kinase-associated neurodegeneration, *PANK2*, c.569insA
Decreased glutathione levels in neurons	Pantothenate kinase-associated neurodegeneration, *PANK2*, c.1259delG<br>Pantothenate kinase-associated neurodegeneration, *PANK2*, c.569insA
Decreased aconitase activity in neurons	Pantothenate kinase-associated neurodegeneration, *PANK2*, c.1259delG<br>Pantothenate kinase-associated neurodegeneration, *PANK2*, c.569insA
Increased TfR1 levels in neurons	Pantothenate kinase-associated neurodegeneration, *PANK2*, c.1259delG<br>Pantothenate kinase-associated neurodegeneration, *PANK2*, c.569insA
Decreased ferritin levels in neurons	Pantothenate kinase-associated neurodegeneration, *PANK2*, c.1259delG<br>Pantothenate kinase-associated neurodegeneration,

	PANK2, c.569insA
Decreased loss of neurons after treatment with CoA	Pantothenate kinase-associated neurodegeneration, *PANK2*, c.1259delG<br>Pantothenate kinase-associated neurodegeneration, *PANK2*, c.569insA
Increased mature firing rate of neurons after treatment with CoA	Pantothenate kinase-associated neurodegeneration, *PANK2*, c.1259delG<br>Pantothenate kinase-associated neurodegeneration, *PANK2*, c.569insA
Decreased ROS levels in neurons after treatment with CoA	Pantothenate kinase-associated neurodegeneration, *PANK2*, c.1259delG<br>Pantothenate kinase-associated neurodegeneration, *PANK2*, c.569insA
Accumulation of cholesterol in cell bodies of neurons	Niemann-Pick disease Type C, *NPC1*, c.3182T>C<br>Niemann-Pick disease Type C, *NPC1,* c.1836A>C/c.1628delC
Accumulation of cholesterol in cellular extensions of neurons	Niemann-Pick disease Type C, *NPC1*, c.3182T>C<br>Niemann-Pick disease Type C, *NPC1,* c.1836A>C/c.1628delC
Increased co-localization of cholesterol/ GM2 in neurons	Niemann-Pick disease Type C, *NPC1*, c.1180T>C<br>Niemann-Pick disease Type C, *NPC1*, c.3182T>C<br>Niemann-Pick disease Type C, *NPC1,* c.1836A>C/c.1628delC
Increased accumulation of osmiophilic material in neurons	Niemann-Pick disease Type C, *NPC1*, c.3182T>C<br>Niemann-Pick disease Type C, *NPC1,* c.1836A>C/c.1628delC
Increased levels of GM2 in neurons	Niemann-Pick disease Type C, *NPC1*, c.3182T>C<br>Niemann-Pick disease Type C, *NPC1,* c.1836A>C/c.1628delC
Decreased levels of GM3 in neurons	Niemann-Pick disease Type C, *NPC1*, c.3182T>C<br>Niemann-Pick disease Type C, *NPC1,* c.1836A>C/c.1628delC
Increased protein levels of Hex A in neurons	Niemann-Pick disease Type C, *NPC1*, c.3182T>C<br>Niemann-Pick disease Type C, *NPC1,* c.1836A>C/c.1628delC
Decreased Hex A enzymatic activity in neurons	Niemann-Pick disease Type C, *NPC1*, c.1180T>C<br>Niemann-Pick disease Type C, *NPC1*, c.3182T>C<br>Niemann-Pick disease Type C, *NPC1,* c.1836A>C/c.1628delC
Decreased intracellular levels of dopamine in dopaminergic neurons	Gaucher Disease, *GBA1*, VS2+1G>T/L444P<br>Gaucher Disease, *GBA1*, N370S
Decreased protein levels of Glucocerebrosidase (Gcase) in dopaminergic neurons	Gaucher Disease, *GBA1*, VS2+1G>T/L444P<br>Gaucher Disease, *GBA1*, N370S
Decreased activity levels of	Gaucher Disease, *GBA1*, VS2+1G>T/L444P

Glucocerebrosidase (Gcase) in dopaminergic neurons	Gaucher Disease, *GBA1*, N370S
Increased protein levels of Glucocerebrosidase (Gcase) in dopaminergic neurons after treatment with NCGC607	Gaucher Disease, *GBA1*, VS2+1G>T/L444P<br>Gaucher Disease, *GBA1*, N370S
Increased activity levels of Glucocerebrosidase (Gcase) in dopaminergic neurons after treatment with NCGC607	Gaucher Disease, *GBA1*, VS2+1G>T/L444P<br>Gaucher Disease, *GBA1*, N370S
Increased translocation of Glucocerebrosidase (Gcase) to the lysosome in dopaminergic neurons after treatment with NCGC607	Gaucher Disease, *GBA1*, VS2+1G>T/L444P<br>Gaucher Disease, *GBA1*, N370S
Increased levels of lysosomal GlcSph in dopaminergic neurons	Gaucher Disease, *GBA1*, VS2+1G>T/L444P<br>Gaucher Disease, *GBA1*, N370S
Decreased levels of lysosomal GlcSph in dopaminergic neurons after treatment with NCGC607	Gaucher Disease, *GBA1*, VS2+1G>T/L444P<br>Gaucher Disease, *GBA1*, N370S
Presence of α-synuclein in soma of dopaminergic neurons	Gaucher Disease, *GBA1*, VS2+1G>T/L444P<br>Gaucher Disease, *GBA1*, N370S
Decreased levels of α-synuclein in dopaminergic neurons after treatment with NCGC607	Gaucher Disease, *GBA1*, VS2+1G>T/L444P<br>Gaucher Disease, *GBA1*, N370S
Presence of co-localized α-synuclein/Lamp2 in dopaminergic neurons	Gaucher Disease, *GBA1*, VS2+1G>T/L444P<br>Gaucher Disease, *GBA1*, N370S
Decreased levels of colocalized α-synuclein/Lamp2 in dopaminergic neurons after treatment with NCGC607	Gaucher Disease, *GBA1*, VS2+1G>T/L444P<br>Gaucher Disease, *GBA1*, N370S

Appendix Table S7. **Overlapping NSC Phenotypes**	
Accumulation of intranuclear RNA foci in NSCs	Dystrophia Myotonica type 1, *DMPK*, Not Specified,<br>Frontotemporal Dementia/Amyotrophic Lateral Sclerosis, *C9ORF72*, GGGGCC Repeats
Decreased amount of TUJ1+ NSCs	Parkinson's Disease, *LRRK2*, G2019S<br>Rett Syndrome, *MeCP2*, Q244X
Decreased amount of Aβ42 in NSCs after treatment with γ-secretase modulator	Alzheimer's Disease, *PSEN1*, A246E<br>Alzheimer's Disease, *APP*, Duplication
Decreased amount of Aβ40 in NSCs after treatment with γ-secretase modulator	Alzheimer's Disease, *PSEN1*, A246E<br>Alzheimer's Disease, *APP*, Duplication
Decreased amount of Aβ38 in NSCs after treatment with γ-secretase modulator	Alzheimer's Disease, *PSEN1*, A246E<br>Alzheimer's Disease, *APP*, Duplication
Decreased heme levels in NSCs	Pantothenate kinase-associated neurodegeneration, *PANK2,* c.1259delG<br>Pantothenate kinase-associated neurodegeneration, *PANK2,* c.569insA
Decreased protein levels of Glucocerebrosidase (Gcase) in NSCs	Gaucher Disease, *GBA1*, N370S<br>Gaucher Disease, *GBA1*, IVS2+1G>T/L444P
Decreased activity levels of Glucocerebrosidase (Gcase) in NSCs	Gaucher Disease, *GBA1*, N370S<br>Gaucher Disease, *GBA1*, IVS2+1G>T/L444P

Appendix Table S8. **Overlapping Astrocyte Phenotypes**	
Accumulation of intranuclear RNA foci in astrocytes	Frontotemporal Dementia/ Amyotrophic Lateral Sclerosis, *C9ORF72*, GGGGCC Repeats<br>Dystrophia Myotonica type 1, *DMPK*, Not Specified
Accumulated Aβ oligomers in astrocytes	Alzheimer's Disease, *APP*, E693Δ<br>Alzheimer's Disease, Sporadic, Not Specified
Increased binding protein (BiP) levels in astrocytes	Alzheimer's Disease, *APP*, E693Δ<br>Alzheimer's Disease, Sporadic, Not Specified
Increased ROS levels in basal conditions in astrocytes	Alzheimer's Disease, *APP*, E693Δ<br>Alzheimer's Disease, Sporadic, Not Specified
Increased ER stress in astrocytes	Alzheimer's Disease, *APP*, E693Δ<br>Alzheimer's Disease, Sporadic, Not Specified
Increased oxidative stress in astrocytes	Alzheimer's Disease, *APP*, E693Δ<br>Alzheimer's Disease, Sporadic, Not Specified
Decreased amount of Aβ oligomers in astrocytes after treatment with β-secretase inhibitor	Alzheimer's Disease, *APP*, E693Δ<br>Alzheimer's Disease, Sporadic, Not Specified
Decrease in ROS in astrocytes after treatment with β-secretase inhibitor	Alzheimer's Disease, *APP*, E693Δ<br>Alzheimer's Disease, Sporadic, Not Specified
Decreased levels of binding protein (BiP) in astrocytes after treatment with β-secretase inhibitor	Alzheimer's Disease, *APP*, E693Δ<br>Alzheimer's Disease, Sporadic, Not Specified

Appendix Table S9. **Overlapping Oligodendrocyte Phenotypes**	
Increased very long chain fatty acid (C26:0/C22:0 ratio) levels in oligodendrocytes	X-Linked Adrenoleukodystrophy, *ABCD1/ ALDP*, AMN Patient<br>X-Linked Adrenoleukodystrophy, *ABCD1/ ALDP*, CCALD Patient
Increased very long chain fatty acid levels in oligodendrocytes compared to AMN ABCD1 (X-linked Adrenoleukodystrophy) mutations	X-Linked Adrenoleukodystrophy, *ABCD1/ ALDP*, AMN Patient<br>X-Linked Adrenoleukodystrophy, *ABCD1/ ALDP*, CCALD Patient
Decreased C26:0/C22:0 ratio levels in oligodendrocytes by lovastatin (through induced upregulation of ABCD2 gene)	X-Linked Adrenoleukodystrophy, *ABCD1/ ALDP*, AMN Patient<br>X-Linked Adrenoleukodystrophy, *ABCD1/ ALDP*, CCALD Patient
Decreased C26:0/C22:0 ratio levels in oligodendrocytes after treatment with 4-PBA (through induced upregulation of ABCD2 gene)	X-Linked Adrenoleukodystrophy, *ABCD1/ ALDP*, AMN Patient<br>X-Linked Adrenoleukodystrophy, *ABCD1/ ALDP*, CCALD Patient

Appendix Table S10. **Phenotypic Organization for Cytoscape Network**			
Gene	Node Number	Phenotype	Level of Evidence
SOD1	1	Recovery of neurofilament subunit proportion (NF-H, NF-M and NF-L) in motor neurons by induction of exogenous NF-L after treatment with Dox (doxycycline)	1
LRRK2	2	Partial rescue of normal nuclear morphology in NSCs after treatment with PI3K inhibitor	1
LRRK2	3	Rescue of aberrant cellular parameters in NSCs after treatment with LRRK2-In-1	1
PGRN	4	Rescue of PI3k/AKT signaling pathway in neurons after ectopic progranulin expression	1
PGRN	5	Rescue of MEK/MAPK signaling pathway in neurons after ectopic progranulin expression	1
NPC1	6	Rescue to normal levels of LC3-II in neurons after treatment with bafilomycin A1	1
NPC1	7	Rescue of defective autophagic levels in neurons after treatment with carbamazepine with or without HP-β-cyclodextrin	1
NPC1	8	Rescue of defective autophagic levels in neurons after treatment with rapamycin with or without HP-β-cyclodextrin	1
NPC1	9	Rescue of defective autophagic levels in neurons after treatment with verapamil with or without HP-β-cyclodextrin	1
NPC1	10	Rescue of defective autophagic levels in neurons after treatment with trehalose with or without HP-β-cyclodextrin	1
MAPT	11	Recovery from oxidative stress after treatment with antioxidant coenzyme Q10 in neurons	1
MAPT	12	Recovery from oxidative stress after treatment with GSK-3 inhibitor CHIR99021 in neurons	1
SHANK3	13	Rescue of synaptic transmission after treatment with IGF1 in neurons	1
APP	14	Partial rescue of cell viability in neurons after treatment with DHA	1
UNC-93-B	15	Rescued replication speed of HSV-1-GFP in neurons after treatment with IFN-α2b	1
TLR3	15	Rescued replication speed of HSV-1-GFP in neurons after treatment with IFN-α2b	1
UNC-93-B	16	Rescued replication speed of HSV-1-GFP in neurons after treatment with IFN-β	1
TLR3	16	Rescued replication speed of HSV-1-GFP in neurons after treatment with IFN-β	1
SHANK3	17	Restored amplitude of evoked AMPA excitatory post-synaptic currents after treatment with IGF1 in neurons	1
SHANK3	18	Restored amplitude of evoked NMDA excitatory post-synaptic currents after treatment with IGF1 in neurons	1
SHANK3	19	Restoration of the number of SYN1 HOMER1 puncta after treatment with IGF1 in neurons	1
UNC-93-B	20	Rescued replication speed of HSV-1-GFP in oligodendrocytes after treatment with IFN-α2b	1
UNC-93-B	21	Rescued replication speed of HSV-1-GFP in oligodendrocytes after treatment with IFN-β	1
SHANK3	22	Rescue of spontaneous excitatory post-synaptic current frequency in neurons after treatment with SC79 (Akt activator)	1
SHANK3	23	Rescue of spontaneous excitatory post-synaptic current frequency	1

		in neurons after treatment with TG003 (Akt activator)	
HTT	24	Near complete rescue of cell death in neurons after treatment with isoxazole-9	1
HTT	25	Partial rescue of cell death in neurons after treatment with isoxazole-9	2
HTT	26	Absence of spontaneously firing neurons after two weeks of differentiation	1
LMNA	27	Absence of progerin (truncated splicing mutant of Lamin A) in iPSCs	1
SMN1	28	Absence of nuclear gems (SMN protein nuclear foci) in neurons	1
SMN1	29	Absence of nuclear gems (SMN protein nuclear foci) in astrocytes	1
SOD1	30	Absence of neurofilament aggregation-induced mitochondrial swelling in motor neurons	1
SOD1	31	Absence of neurofilament aggregation-induced vacuole formation in motor neurons	1
MeCP2	32	Absence of random X-inactivation in iPSCs	1
ATP7A	33	Absence of differentiation into TuJ1- expressing neurites in iPSCs	1
ATP7A	34	Absence of differentiation into MAP2- expressing neurites in iPSCs	1
GAA	35	Accumulation of glycogen granules in cytoplasm in iPSCs	1
GBA1	36	Accumulation of monomeric α-synuclein in neurons	1
Parkin	36	Accumulation of monomeric α-synuclein in neurons	1
SNCA	36	Accumulation of monomeric α-synuclein in neurons	1
LRRK2	36	Accumulation of monomeric α-synuclein in neurons	1
APP	37	Accumulation of Aβ oligomers in neurons	1
Sporadic (AD)	37	Accumulation of Aβ oligomers in neurons	1
C9ORF72	38	Accumulation of intranuclear RNA foci in neurons	1
DMPK	38	Accumulation of intranuclear RNA foci in neurons	1
LRRK2	39	Accumulation of SNCA in dopaminergic neurons	1
SNCA	39	Accumulation of SNCA in dopaminergic neurons	1
DMPK	40	Accumulation of intranuclear RNA foci in NSCs	1
C9ORF72	40	Accumulation of intranuclear RNA foci in NSCs	1
C9ORF72	41	Accumulation of intranuclear RNA foci in astrocytes	1
DMPK	41	Accumulation of intranuclear RNA foci in astrocytes	1
GBA1	42	Accumulation of lysosomes in neurons (as shown by LAMP1 positive particles)	1
SNCA	43	Accumulation of oligomerized α-synuclein in neurons	1
SNCA	44	Accumulation of ER-associated degradation substances (Gcase, Nicastrin) in neurons	1
Parkin	45	Accumulation of abnormal tubulovesicular structures in neurons	1
DMPK	46	Accumulation of intranuclear RNA foci in iPSCs	1
TDP-43	47	Accumulation of TDP-43 pre-inclusion like aggregates in cytoplasm in neurons	1
TDP-43	48	Accumulation of soluble TDP-43 in astrocytes	1

C9ORF72	49	Accumulation of RNA foci in neurons	1
TDP-43	50	Accumulation of TDP43 in neurons	1
SOD1	51	Accumulation of neurofilament aggregation in motor neurons	1
APP	52	Accumulated Aβ oligomers in astrocytes	1
Sporadic (AD)	52	Accumulated Aβ oligomers in astrocytes	1
PSEN1	53	Accumulation of APP-CTFs upon exposure to DAPT in neurons	1
GBA1	54	Accumulation of glucosylsphingolipids in neurons	1
FUS	55	Accumulation of FUS cytoplasmic aggregates in motor neurons	1
FUS	56	Accumulation of high FUS protein levels within stress granules after treatment with sodium arsenite in neurons	1
FUS	57	Accumulation of high FUS protein levels within stress granules after treatment with sodium arsenite in iPSCs	1
FUS	58	Accumulation of high FUS protein levels within stress granules after temperature stress in neurons	1
FUS	59	Accumulation of high FUS protein levels within stress granules after temperature stress in iPSCs	1
FUS	60	Accumulation of high FUS protein levels within stress granules in iPSCs after treatment with sorbitol	1
GBA1	61	Accumulation of monomeric α-synuclein in midbrain dopaminergic neurons	1
SOD1	62	Accumulation of detergent-insoluble SOD1 in motor neurons after treatment with MG132	1
hnRNPA2B1	63	Accumulation of hnRNP A2/B1+ stress granules in motor neurons after exposure to puromycin	1
VCP	63	Accumulation of hnRNP A2/B1+ stress granules in motor neurons after exposure to puromycin	1
NPC1	64	Accumulation of cholesterol in cell bodies of neurons	2
NPC1	65	Accumulation of cholesterol in cellular extensions of neurons	2
TDP-43	66	Presence of mislocalization of TDP-43 protein in cytoplasm of astrocytes	1
SCN1A	67	Presence of spontaneous repetitive firing activity in neurons	1
LMNA	68	Presence of premature senescence phenotypes in iPSCs	1
FXN	69	Presence of delayed development of electrophysiological functionality in iPSCs	1
MAPT	70	Presence of mild degeneration of neuronal processes	1
MAPT	71	Presence of AT8-positive p-TAU in predominant and punctate locations in axons of neurons	1
MAPT	72	Presence of constricted/ tapered neurites in neurons	1
MAPT	73	Presence of severe axonal degeneration in neurons	1
PLP1	74	Presence of mislocalization of PLP1 protein to the ER in oligodendrocytes	1
PLP1	75	Presence of aberrant ER morphologies in oligodendrocytes	1
ATP7A	76	Presence of an aberrant switch of E-cadherin to N-cadherin in	1

		iPSCs	
ATP7A	77	Presence of abnormal neural rosette during early differentiation in iPSCs	1
ATP7A	78	Presence of an abnormal reticular distribution of ATP7A in iPSCs	1
LRRK2	79	Presence of fragmented nuclei in neurons	1
LRRK2	80	Presence of vacuolated soma in neurons	1
LRRK2	81	Presence of spontaneous NSC differentiation at late passages	1
LRRK2	82	Presence of aberrant non-neuronal cellular morphology in NSCs	1
SNCA	83	Presence of Lewy neurite/body-like α-synuclein deposition in neurons	1
PINK1	84	Presence of energy substrate (ATP)- independent respiration in neurons	1
SMN1	85	Presence of fewer/smaller end plates in motor neurons	1
ATXN3	86	Presence of SDS-insoluble ATXN3-containing fragments generation in neurons	1
C9ORF72	87	Presence of co-localization of RNA foci in neurons with hnRNPA1	1
C9ORF72	88	Presence of co-localization of RNA foci in neurons with Pur-α	1
C9ORF72	89	Presence of repeat-associated non-ATG (RAN) translation products in neurons	1
PLP1	90	Presence of mis-localization of PLP1 protein in the perinuclear cytoplasm in oligodendrocytes	1
PLP1	91	Presence of co-localization of PLP1 protein with KDEL in oligodendrocytes	1
PLP1	92	Presence of scatter O4 staining in the processes of oligodendrocytes	1
PLP1	93	Presence of increased ER intermembrane space dilation in oligodendrocytes	1
FUS	94	Presence of mis-localized FUS protein in the cytoplasm of motor neurons	1
ATP7A	95	Presence of abnormal epithelial morphology of attached cells from embryoid bodies in iPSCs	1
SOD1	96	Presence of abnormal mitochondrial morphology in motor neurons	1
DHCR7	97	Presence of poorly defined SOX2+/ PAX6+ neural rosette structures in iPSCs	1
DHCR7	98	Presence of long neuronal projections in iPSCs after extended differentiation	1
DHCR7	99	Presence of spindled neural progenitor-like morphology in iPSCs when cultured in cholesterol-deficient mTesR1 medium	1
PANK2	100	Presence of abnormal mitochondrial morphology (swollen, damaged cristae) in neurons	2
GBA1	101	Presence of α-synuclein in soma of dopaminergic neurons	2
GBA1	102	Presence of co-localized α-synuclein/Lamp2 in dopaminergic neurons	

PANK2	103	Presence of abnormal firing activity in neurons after injection of suprathreshold current steps	2
CACNA1C	104	Impaired calcium channel inactivation in neurons	1
ATP7A	105	Impaired structural integrity in the cell to cell junction (membrane recruitment/microdetachment) in iPSCs	1
ATP7A	106	Impaired gradual decrease of E-cadherin in iPSC derivatives	1
NPC1	107	Impaired reduction of nSREBP-2 levels in neurons after exposure to HP-β-cyclodextrin serum	1
MeCP2	108	Impaired neuronal network connectivity in glutamatergic neurons	1
MAPT	108	Impaired neuronal network connectivity in glutamatergic neurons	1
UNC-93-B	109	Impaired induction of IFN-β and IFN-γ by NSCs after exposure to polyinosinic:polycytidylic acid	1
UNC-93-B	110	Impaired induction of IFN-β and IFN-γ by astrocytes after exposure to polyinosinic:polycytidylic acid	1
UNC-93-B	111	Impaired induction of IFN-β and IFN-γ by oligodendrocytes after exposure to polyinosinic:polycytidylic acid	1
GBA1	112	Impaired lysosomal system in neurons	1
GBA1	113	Impaired intracellular calcium homeostasis in neurons	1
GBA1	114	Impaired autophagosome-lysosome fusion in neurons	1
LRRK2	115	Impaired nuclear architecture in neurons	1
LRRK2	116	Impaired formation of MAP2+ NSCs	1
LRRK2	117	Impaired ability to retain clonogenic and differentiation capacity in NSCs	1
SNCA	118	Impaired maximal rate of mitochondrial respiration in neurons	1
Parkin	119	Impaired reduction of inner mitochondrial membrane area in neurons after exposure to Carbonyl cyanide m-chlorophenyl hydrazone (CCCP)	1
PINK1	120	Impaired reduction of mtDNA upon mitochondrial depolarization in neurons	1
UNC-93-B	121	Impaired induction of IFN-β and IFN-γ by oligodendrocytes following HSV-1 infection	1
UNC-93-B	122	Impaired induction of MX1 (IFN-inducible molecule) by oligodendrocytes following HSV-1 infection	1
UNC-93-B	123	Impaired induction of NF-κB1 and MX1 (IFN-inducible molecule) by oligodendrocytes after exposure to polyinosinic:polycytidylic acid	1
MeCP2	124	Impairment of neuronal maturation	1
UNC-93-B	125	Impairment of neuronal induction of IFN-β and IFN-γ in response to polyinosinic:polycytidylic acid	1
APP	126	Impairment of Golgi function in neurons	1
APP	127	Impairment of ER function in neurons	1
PSEN1	128	Partial impairment of γ-secretase function in neurons	1
PINK1	129	Impairment of induced ubiquitination of Mfn2 protein after treatment with valinomycin in neurons	1

PINK1	130	Impairment of mitochondrial translocation of Parkin in neurons after exposure to valinomycin	1
SMN1	131	Impairment of the Fas-mediated pathway in motor neurons after exposure to antagonist ZB4 clone of anti-FAS monoclonal antibody (FasNT Ab)	1
SMN1	132	Impairment of the Fas-mediated pathway in motor neurons after exposure to caspase-3 specific inhibitor Z-DVED-FMK	1
UNC-93-B	133	Impairment of neuronal induction of IFN-β and IFN-γ in response to HSV-1 infection	1
CYFIP1	134	Impaired structure of adherens junctions in NSCs	1
UNC-93-B	135	Impaired reductions in HSV-1-GFP replication levels in NSCs after treatment with polyinosinic:polycytidylic acid	1
SHANK3	136	Impairment of AMPA-mediated synaptic transmission in neurons	1
SHANK3	137	Impairment of NMDA-mediated synaptic transmission in neurons	1
SHANK3	138	Impairment of Akt activity in neurons	1
UNC-93-B	139	Decreased susceptibility of neurons to HSV-1 infection after treatment with exogenous IFN-a/ β	1
TLR3	139	Decreased susceptibility of neurons to HSV-1 infection after treatment with exogenous IFN-a/ β	1
PINK1	140	Decreased susceptibility of neurons to low concentrations of valinomycin induced cell death after treatment with coenzyme Q10	1
LRRK2	140	Decreased susceptibility of neurons to low concentrations of valinomycin induced cell death after treatment with coenzyme Q10	2
LRRK2	141	Decreased susceptibility of neurons to low concentrations of concanamycin A induced cell death after treatment with coenzyme Q10	2
PINK1	141	Decreased susceptibility of neurons to low concentrations of concanamycin A induced cell death after treatment with coenzyme Q10	1
LRRK2	142	Decreased susceptibility of neurons to valinomycin induced cell death after treatment with LRRK2 inhibitor GW5074	2
PINK1	142	Decreased susceptibility of neurons to valinomycin induced cell death after treatment with LRRK2 inhibitor GW5074	1
LRRK2	143	Decreased susceptibility of neurons to concanamycin A induced cell death after treatment with LRRK2 inhibitor GW5074	1
LRRK2	144	Decreased susceptibility of neurons to valinomycin induced cell death after treatment with rapamycin	1
UNC-93-B	145	Decreased susceptibility of oligodendrocytes to HSV-1 infection after treatment with exogenous IFN-α/ β	1
PGRN	146	Decreased susceptibility of neurons to PI3K inhibitor after ectopic progranulin expression	1
PGRN	147	Decreased susceptibility to MEK kinase inhibitor after ectopic progranulin expression in neurons	1
PGRN	148	Decreased susceptibility to wortmannin after ectopic progranulin	1

		expression in neurons	
PGRN	149	Decreased susceptibility to staurosporine after ectopic progranulin expression in neurons	1
TDP-43	150	Decreased susceptibility of neurons to arsenite-induced death after exposure to anacardic acid	1
TDP-43	151	Increase in susceptibility of neurons to death after exposure to arsenite	1
LRRK2	152	Increased susceptibility of neurons to 6-OHDA exposure (DA neurons)	1
SNCA	153	Increased apoptotic susceptibility in dopaminergic neurons	1
UNC-93-B	154	Increased susceptibility of neurons to HSV-1 infection	1
TLR3	154	Increased susceptibility of neurons to HSV-1 infection	1
LRRK2	155	Increased susceptibility of neurons to valinomycin	2
PINK1	155	Increased susceptibility of neurons to valinomycin	1
LRRK2	156	Increased susceptibility of neurons to MPP+	2
PINK1	156	Increased susceptibility of neurons to MPP+	1
LRRK2	157	Increased susceptibility of neurons to concanamycin A	2
PINK1	157	Increased susceptibility of neurons to concanamycin A	1
LRRK2	158	Increased susceptibility of neurons to MG132 exposure (DA neurons)	2
PINK1	158	Increased susceptibility of neurons to MG132 exposure (DA neurons)	1
HTT	159	Increased susceptibility of neurons to hydrogen peroxide	1
PINK1	159	Increased susceptibility of neurons to hydrogen peroxide	1
SNCA	159	Increased susceptibility of neurons to hydrogen peroxide	1
GBA1	160	Increased susceptibility to ER stress in neurons by calcimycin	1
GBA1	161	Increased susceptibility to ER stress in neurons by rotenone	1
LRRK2	162	Increased susceptibility to caspase-3 activation in neurons	1
SNCA	163	Increased susceptibility to Manganese ethylnebisdithiocarbamate mediated-apoptosis in neurons	1
SNCA	164	Increased susceptibility to Paraquat mediated-apoptosis in neurons	1
SNCA	165	Increased susceptibility of neurons to rotenone mediated-apoptosis	1
MAPT	165	Increased susceptibility of neurons to rotenone mediated-apoptosis	3
SNCA	166	Increased susceptibility of neuronal intrinsic aberrant protein aggregation and stress	1
UNC-93-B	167	Increased susceptibility of oligodendrocytes to HSV-1 infection	1
PGRN	168	Increased susceptibility of neurons to wortmannin	1
PGRN	169	Increased susceptibility of neurons to MEK kinase inhibitor	1
PGRN	170	Increased susceptibility of neurons to ER stress by tunicamycin	1
PGRN	171	Increased susceptibility of neurons to proteasome activity inhibition due to lactacystin	1
PGRN	172	Increased susceptibility in neurons to staurosporine	1
TDP-43	173	Increased susceptibility of neurons to antagonism of the PI3K	1

		pathway	
HTT	174	Increased susceptibility to 3-methyladenine (3-MA) in neurons	1
HTT	175	Increased susceptibility of NSCs to death after growth factor withdrawal	1
LRRK2	176	Increased neuronal susceptibility to oxidative stress when cultured in N2 medium without the supplement B27	1
HTT	177	Increased susceptibility to oxidative stress in iPSCs	1
PGRN	178	Increased susceptibility of neurons to PI3K inhibitor	1
HTT	179	Increased susceptibility of iPSCs to form huntingtin aggregates after exposure to MG132	1
MAPT	180	Increased susceptibility to MAPT-induced neurotoxicity in DA neurons	1
PLP1	181	Increased susceptibility to ER stress induced by exposure to tunicamycin in oligodendrocytes	1
PLP1	182	Increased susceptibility to ER stress in oligodendrocytes	1
MAPT	183	Increased susceptibility to oxidative stress in neurons after treatment with rotenone	1
MAPT	184	Increased susceptibility to oxidative stress in astrocytes	1
GBA1	185	Increased amount of autophagosomes in neurons	1
LRRK2	185	Increased amount of autophagosomes in neurons	1
NPC1	185	Increased amount of autophagosomes in neurons	1
PGRN	186	Increased percentage of neurons containing cytoplasmic TDP-43	1
TDP-43	186	Increased percentage of neurons containing cytoplasmic TDP-43	2
APP	187	Increased Aβ42/40 ratio in neurons	2
PSEN2	187	Increased Aβ42/40 ratio in neurons	1
PSEN1	187	Increased Aβ42/40 ratio in neurons	4
PSEN1	188	Increased extracellular Aβ42 in neurons	1
PSEN2	188	Increased extracellular Aβ42 in neurons	1
APP	188	Increased extracellular Aβ42 in neurons	1
LRRK2	189	Increased positive staining for cleaved caspase-3 in neurons	1
SMN1	189	Increased positive staining for cleaved caspase-3 in neurons	1
APP	190	Increased levels of TAU protein in neurons	1
MAPT	190	Increased levels of TAU protein in neurons	1
LRRK2	190	Increased levels of TAU protein in neurons	1
ABCD1	191	Increase in very long chain fatty acid (C26:0/C22:0 ratio) levels in neurons	1
ALDP	191	Increase in very long chain fatty acid (C26:0/C22:0 ratio) levels in neurons	1
APP	192	Increased oxidative stress in neurons	1
Sporadic (AD)	192	Increased oxidative stress in neurons	1
LRRK2	192	Increased oxidative stress in neurons	1
SNCA	192	Increased oxidative stress in neurons	1
Parkin	192	Increased oxidative stress in neurons	1

TDP-43	192	Increased oxidative stress in neurons	2
PINK1	192	Increased oxidative stress in neurons	1
MAPT	192	Increased oxidative stress in neurons	1
APP	193	Increased ER stress in neurons	1
SNCA	193	Increased ER stress in neurons	1
MAPT	193	Increased ER stress in neurons	2
Sporadic (AD)	193	Increased ER stress in neurons	1
NPC1	194	Increased risk of neuronal death	1
HTT	194	Increased risk of neuronal death	3
LRRK2	194	Increased risk of neuronal death	1
PANK2	194	Increased risk of neuronal death	1
ABCD1	195	Increased very long chain fatty acid (C26:0/C22:0 ratio) levels in oligodendrocytes	1
ALDP	195	Increased very long chain fatty acid (C26:0/C22:0 ratio) levels in oligodendrocytes	1
APP	196	Increased levels of cleaved caspase-4 in neurons	1
APP	197	Increased levels of binding protein (BiP) in neurons	1
Sporadic (AD)	197	Increased levels of binding protein (BiP) in neurons	1
MAPT	197	Increased levels of binding protein (BiP) in neurons	1
APP	198	Increased amount of large Rab5+ early endosomes in neurons	1
APP	199	Increased Aβ 38 secretion in neurons	1
APP	200	Increased Aβ 38/40 ratio in neurons	1
APP	201	Increased generation of APPs-α relative to APPs-β in neurons after treatment with γ-secretase inhibition	1
APP	202	Increased β-secretase cleavage at APP in neurons	1
APP	203	Increased colocalization coefficient of APP with endosomal marker EEA1 in neurons	1
APP	204	Increased levels of phospho-Tau at amino acid S262 in neurons	1
APP	205	Increased secretion of Aβ (1-40) by neurons	1
APP	206	Increased amount of anti-glycogen synthase kinase 3 beta (aGSK-3β) in neurons	1
APP	207	Increased p-tau/total tau ratios in neurons	1
GBA1	208	Increased intracellular calcium levels in neurons	1
GBA1	209	Increased amount of cytosolic calcium induced by caffeine in neurons	1
GBA1	210	Increase in RyR-mediated calcium release in neurons	1
LRRK2	211	Increase in bidirectional movement of mitochondria in the proximal axon of neurons	1
LRRK2	212	Increase in LC3-positive puncta (antibody) in neurons	1
LRRK2	213	Increased levels of nucleoporin p62 protein in neurons	1
LRRK2	214	Increased abundance of autophagic vacuoles in neurons	1
LRRK2	215	Increased amount of TH-positive neurons after exposure to MG132	1
LRRK2	216	Increased levels of pERK in neurons	1

LRRK2	217	Increased levels of phospho-Thr181 TAU in neurons	1
LRRK2	218	Increase in DA neuron survival in the presence of LRRK2-IN1	1
LRRK2	219	Increased neurite growth rates in neurons after treatment with LRRK2-IN1	1
LRRK2	220	Increased neurite growth rates after treatment with MEK kinase inhibitor in neurons	1
LRRK2	221	Increased levels of TH and cleaved CASPASE3 double positive neurons after treatment with rotenone	1
LRRK2	222	Increased levels of TH and cleaved CASPASE3 double positive neurons after treatment with 6-OHDA	1
LRRK2	223	Increased nuclear disruption in NSCs (enlarged nuclear area, decrease in circularity, and loss of lamin B1 and B2 on specific folds of nuclear envelope)	1
LRRK2	224	Increased proteasomal stress in NSCs	1
APP	225	Increased oxidative stress in neurons after exposure to hydrogen peroxide	1
LRRK2	225	Increased oxidative stress in neurons after exposure to hydrogen peroxide	1
LRRK2	226	Increased apoptosis in NSCs after exposure to MG132	1
LRRK2	227	Increased amount of centromeric signals accompanied by reorganization of centromeric heterochromatin in NSCs	1
SNCA	228	Increase in basal levels of NO (RNS) in neurons	1
LRRK2	229	Increased phosphorylation of lamin B1 and B2 in NSCs	1
SNCA	230	Increased accumulation of NO in neurons after exposure to mitochondrial toxins (Paraquat or Maneb)	1
SNCA	231	Increased ROS levels in basal conditions in neurons	1
APP	231	Increased ROS levels in basal conditions in neurons	1
Sporadic (AD)	231	Increased ROS levels in basal conditions in neurons	1
PANK2	231	Increased ROS levels in basal conditions in neurons	2
SNCA	232	Increased damage to neurons by inhibition of SOH-MEF2C (Sulfenated-myocyte enhancer factor 2C) after exposure to hydrogen peroxide	1
SNCA	233	Increased damage to neurons by inhibition of SOH-MEF2C (Sulfenated-myocyte enhancer factor 2C) after exposure to Paraquat	1
SNCA	234	Increased damage to neurons by inhibition of SOH-MEF2C (Sulfenated-myocyte enhancer factor 2C) after exposure to Manganese ethylnebisdithiocarbamate	1
SNCA	235	Increased amount of caspase-3 positive TH neurons after exposure to hydrogen peroxide	1
SNCA	236	Increase in levels of nitrative stress in neurons	1
SNCA	237	Increased ratio of post ER-to-ER forms in neurons after treatment with NAB2	1

SNCA	238	Increased levels of secreted α-synuclein by neurons that can be reuptaken by neighboring neurons	1
Parkin	239	Increased spontaneous dopamine release by neurons	1
Parkin	240	Increased MAO A and B transcription in neurons	1
Parkin	241	Increased dopamine-induced oxidative stress in neurons	1
Parkin	242	Increased amount of protein carbonyls in neurons	1
Parkin	243	Increased MAO-A and MAO-B enzymatic activities in neurons	1
Parkin	244	Increased electron density in matrix of the inner mitochondrial membrane of neurons	1
Parkin	245	Increased perikaryal volume in mitochondria of neurons	1
Parkin	246	Increased swelling of mitochondrial cristae in the inner mitochondrial membrane of neurons	1
Parkin	247	Increased activation of Nrf2 pathway in neurons	1
Parkin	248	Increased 2',7'- dichoorodihydrofloursecin (DCF) fluorescence in neurons	1
PINK1	249	Increase in passive leak of protons from inner mitochondrial membrane of neurons	1
PINK1	250	Increased Basal Oxygen Consumption Rate (OCR) in neurons	1
PINK1	251	Increase in PGC-1α levels in neurons	1
PINK1	252	Increased generation of mROS after exposure to low concentrations of valinomycin in neurons	1
SMN1	253	Increased amount of nuclear gems (SMN protein nuclear foci) in neurons after treatment with valproic acid	1
SMN1	254	Increased amount of nuclear gems (SMN protein nuclear foci) in neurons after treatment of tobramycin	1
SMN1	255	Increased SMN protein levels in neurons after treatment of valproic acid	1
SMN1	256	Increased SMN protein levels in neurons after treatment of tobramycin	1
SMN1	257	Increased percentage of apoptotic neurons	1
SMN1	258	Increase activation of initiator procaspase-8 to its cleaved form in neurons	1
SMN1	259	Increased amount of cleaved caspase-8 positive neurons	1
SMN1	260	Increased amount of membrane bound Fas-ligand in neurons	1
SMN1	261	Increased amounts of nuclear gems (SMN protein nuclear foci) in astrocytes after treatment with valproic acid	1
SMN1	262	Increased amounts of nuclear gems (SMN protein nuclear foci) in astrocytes after treatment with tobramycin	1
SMN1	263	Increased SMN protein levels in astrocytes after treatment with valproic acid	1
SMN1	264	Increased SMN protein levels in astrocytes after treatment with tobramycin	1
MeCP2	265	Increase in glutamatergic synapse amount in neurons after	1

		treatment of IGF1	
MeCP2	266	Increased glutamatergic synapse amount and MeCP2 protein levels in neurons after treatment with gentamicin	1
CDKL5	267	Increased amount of aberrant dendritic spines in neurons	1
PGRN	268	Increased percentage of neurons with cytoplasmic TDP-43 after exposure to tunicamycin	1
PGRN	269	Increased percentage of neurons with cytoplasmic TDP-43 after exposure to staurosporine	1
PGRN	270	Increased caspase-3 activity in neurons after exposure to staurosporine	1
PGRN	271	Increased caspase-3 activity in neurons after exposure to tunicamycin	1
PGRN	272	Increased levels of S6K2 in neurons after ectopic progranulin expression	1
TDP-43	273	Increased levels of soluble and detergent-resistant TDP-43 protein in neurons	1
TDP-43	274	Increase in detergent-insoluble TDP-43 in neurons	1
TDP-43	275	Increased amount of SNRPB2 bound to TDP-43 in neurons	1
TDP-43	276	Increased neurite length in neurons after exposure to anacardic acid	1
TDP-43	277	Increased risk of death in astrocytes under basal conditions	1
SOD1	278	Increase in SOD1 aggregates in cytoplasm, nuclei and neurites of motor neurons	1
SOD1	279	Increased neurite degeneration in motor neurons	1
CACNA1C	280	Increased secretion of dopamine in neurons	1
CACNA1C	281	Increased secretion of norepinephrine in neurons	1
CACNA1C	282	Increase in sustained calcium ion rise following depolarization of neurons	1
CACNA1C	283	Increase in fraction of neurons expressing TH	1
CACNA1C	284	Increased proportion of neurons expressing upper layer markers (CUX1 and REELIN)	1
HTT	285	Increased death of neurons with large CAG expansions	1
HTT	286	Increased risk of neuronal death with BDNF withdrawal	2
HTT	287	Increased calcium dyshomeostatis in neurons after exposure to pathological glutamate levels	1
HTT	288	Increased neuronal death after exposure to 30 minute glutamate pulses	1
HTT	289	Increased mitochondrial fragmentation along neurites of GABA neurons	1
HTT	290	Increased mROS formation in medium spiny neurons	1
HTT	291	Increased neurite length after treatment with dynamin-related protein 1 peptide inhibitor P110-TAT in neurons	1
HTT	292	Increased levels of ATP in medium spiny neurons after treatment	1

		with dynamin-related protein 1 peptide inhibitor P110-TAT	
HTT	293	Increased mitochondrial membrane potential after treatment with dynamin-related protein 1 peptide inhibitor P110-TAT in medium spiny neurons	1
HTT	294	Increase in caspase-3/7 activity in NSCs upon growth factor deprivation	1
HTT	295	Increase in TUNEL-positive NSCs	1
HTT	296	Increased electron clear vacuoles under basal conditions in cytoplasm of astrocytes	1
HTT	297	Increased apoptotic cell death in iPSCs through activation of the p53-mediated apoptotic pathway	1
HTT	298	Increased amount of peroxiredoxin1, peroxiredoxin2 and peroxiredoxin6 in iPSCs	1
HTT	299	Increased amount of TUNEL-positive iPSCs	1
HTT	300	Increased expression of BTF3 and ATM in iPSCs	1
ALDP	301	Increased very long chain fatty acid levels in oligodendrocytes compared to AMN ABCD1 (X-linked Adrenoleukodystrophy) mutations	1
ABCD1	301	Increased very long chain fatty acid levels in oligodendrocytes compared to AMN ABCD1 (X-linked Adrenoleukodystrophy) mutations	1
SCN1A	302	Increased sodium current in neurons	1
SCN1A	303	Increased mean sodium current densities in neurons	1
SCN1A	304	Increased repetitive firing potential in neurons	1
SCN1A	305	Increased excitability in neurons	1
C9ORF72	305	Increased excitability in neurons	1
TDP-43	305	Increased excitability in neurons	1
SMN1	305	Increased excitability in neurons	1
SCN1A	306	Increased amount of depolarized resting membrane potentials (bipolar shaped neurons)	1
FXN	307	Increased expression of MSH2 enzyme in iPSCs	1
FXN	308	Increased GAA-TTC repeat instability in iPSCs	1
FXN	309	Increased instability of GAA repeats in iPSCs	1
MAPT	310	Increased amount of caspase-cleaved TAU fragmentation in neurons	1
MAPT	311	Increased neurite fragmentation/ degeneration in glutamatergci neurons	1
MAPT	312	Increased neurite fragmentation/ degeneration in GABAergic neurons	1
PLP1	313	Increased amount of apoptotic cells in oligodendrocytes	1
ATP7A	314	Increased distribution of ATP7A molecules throughout cytoplasm in iPSCs	1
ATP7A	315	Increased opacity/density embryoid body structures in iPSCs	1

CACNA1C	316	Increased proportion of CTIP2 expressing neurons in lower layer marker (FOXP1 and ETV1) expressing neurons	1
GAA	317	Increased positive staining for Periodic acid-Schiff in iPSCs	1
ERCC6	318	Increased iPSC death in absence of CSB	1
ERCC6	319	Increased levels of ROS in iPSCs	1
LRRK2	320	Increased levels of mitochondrial DNA damage in NSCs	1
LRRK2	321	Increase in mobility of mitochondria in the proximal axon of neurons	1
APP	322	Increased levels of cleaved caspase-4 in astrocytes	1
APP	323	Increased binding protein (BiP) levels in astrocytes	1
Sporadic (AD)	323	Increased binding protein (BiP) levels in astrocytes	1
APP	324	Increased ROS levels in basal conditions in astrocytes	1
Sporadic (AD)	324	Increased ROS levels in basal conditions in astrocytes	1
APP	325	Increased ER stress in astrocytes	1
Sporadic (AD)	325	Increased ER stress in astrocytes	1
APP	326	Increased oxidative stress in astrocytes	1
Sporadic (AD)	326	Increased oxidative stress in astrocytes	1
HTT	327	Increase in caspase-3/7 activity in neurons upon BDNF withdrawal	1
NPC1	328	Increased cholesterol accumulation in LE/L compartment in neurons	1
PLP1	329	Increased nuclear condensation in apoptotic oligodendrocytes	1
PSEN1	330	Increased amount of Aβ42 in neurons	1
PSEN1	331	Increased production of Aβ39 in neurons after treatment with γ-secretase modulator	1
PSEN1	332	Increased production of Aβ37 in neurons after treatment with γ-secretase modulator	1
Parkin	333	Increase in free tubulin in neurons	1
Parkin	334	Increased neurite length after treatment with taxol in neurons	1
Parkin	335	Increased number of terminals after treatment with taxol in neurons	1
Parkin	336	Increased number of branch points after treatment with taxol in neurons	1
Parkin	337	Increased neurite complexity after treatment with taxol in neurons	1
NPC1	338	Increase in early cell death in neurons	1
NPC1	339	Increased cell survival in neurons after treatment with curcumin	1
NPC1	340	Increased cell survival in neurons after treatment with dantrolene	1
MAPT	341	Increased fragmentation of TAU protein in neurons	1
SMN1	342	Increased membrane resistance in motor neurons	1
SMN1	343	Increased Na+ current in motor neurons	1
PSEN1	344	Increased Aβ42/40 ratio in NSCs	1
SHANK3	345	Increased amplitude of spontaneous excitatory post-synaptic currents after treatment with IGF1 in neurons	1
SHANK3	346	Increased frequency of spontaneous excitatory post-synaptic currents after treatment with IGF1 in neurons	1
SHANK3	347	Increased current size generation in response to focal application of NMDA after treatment with IGF1 in neurons	1

SHANK3	348	Increased rate of decay of NMDA-excitatory post-synaptic currents in neurons	1
SHANK3	349	Increased input resistance in neurons	1
NPC1	350	Increased cell viability in neurons after treatment with carbamazepine with or without HP-β-cyclodextrin	1
NPC1	351	Increased cell viability in neurons after treatment with rapamycin with or without HP-β-cyclodextrin	1
NPC1	352	Increased cell viability in neurons after treatment with trehalose with or without HP-β-cyclodextrin	1
MAPT	353	Increased immunoreactivity for AT8 in neurons	1
NPC1	354	Increased cell viability in neurons after treatment with verapamil with or without HP-β-cyclodextrin	1
CACNA1C	355	Increase in sustained calcium ion rise following depolarization of NSCs	1
UNC-93-B	356	Increased replication speed of HSV-1-GFP in oligodendrocytes	1
UNC-93-B	357	Increased replication speed of HSV-1-GFP in neurons	1
TLR3	357	Increased replication speed of HSV-1-GFP in neurons	1
APP	358	Increased levels of binding protein (BiP) in neurons after treatment with high concentrations of DHA	1
HTT	359	Increased levels of mitochondrial associated dynamin-related protein 1 in iPSCs	1
HTT	360	Increased levels of mitochondrial associated p53 in iPSCs	1
FUS	361	Increased punctuate cytoplasmic delocalization of FUS protein in iPSCs	1
TDP-43	362	Increased amount of TDP-43 insoluble fractions in neurons after exposure to arsenite	1
SOD1	363	Increase in TUNEL-positive motor neurons	1
SOD1	364	Increased levels of soluble SOD1 in motor neurons after treatment with MG132	1
SOD1	365	Increase in mitochondrial density in processes in motor neurons	1
SOD1	366	Increased motor neuron survival after treatment with salubrinal	1
GBA1	367	Increase in α-synuclein immunoreactivity in midbrain dopaminergic neurons	1
DISC1	368	Increased soma size in neurons at one and two weeks post-differentiation	1
HTT	369	Increased neurite-like processes length in neurons	3
HTT	370	Increased nuclear:cytoplasmic ratio of endogenous MAP2 in neurons	2
C9ORF72	371	Increased LIMK-1/2 phosphorylation in motor neurons	1
C9ORF72	372	Increased levels of Rac1 in motor neurons	1
C9ORF72	373	Increased cofflin phosphorylation in motor neurons	1
DHCR7	374	Increased 7-, 8- DHC levels in iPSCs after exposure to cholesterol-deficient mTesR1 medium	4

DHCR7	375	Increased rates of neuronal differentiation in iPSCs	2
DHCR7	376	Increased gap and tight junctions in iPSCs cultured in cholesterol-deficient mTesR1 medium after treatment with LDL	2
DHCR7	377	Increased formation of rosette structures in iPSCs iPSCs cultured in cholesterol-deficient mTesR1 medium after treatment with LDL	2
hnRNPA2B1	378	Increased levels of nuclear, insoluble of hnRNP A2/B1in motor neurons	1
VCP	378	Increased levels of nuclear, insoluble of hnRNP A2/B1in motor neurons	1
PANK2	379	Increased proportion of altered mitochondria in neurons	1
PANK2	380	Increased TfR1 levels in neurons	1
PANK2	381	Increased mature firing rate of neurons after treatment with CoA	1
MAPT	382	Increased levels of insoluble P-tau protein in neurons	1
MAPT	383	Increased levels of insoluble tau in neurons	1
MAPT	384	Increased levels of polyubiquitinated proteins in neurons	1
MAPT	385	Increased levels of autophagy markers in neurons	1
MAPT	386	Increased levels of CHOP protein in neurons	1
MAPT	387	Increased cell viability of neurons after treatment with rapamycin	1
MAPT	388	Increased cell viability of neurons exposed to rotenone after treatment with rapamycin	1
MAPT	389	Increased cell viability of neurons exposed to NMDA after treatment with rapamycin	1
MAPT	390	Increased cell viability of neurons exposed to Aβ(1-42) after treatment with rapamycin	1
GBA1	391	Increased protein levels of Glucocerebrosidase (Gcase) in dopaminergic neurons after treatment with NCGC607	2
GBA1	392	Increased activity levels of Glucocerebrosidase (Gcase) in dopaminergic neurons after treatment with NCGC607	2
GBA1	393	Increased translocation of Glucocerebrosidase (Gcase) to the lysosome in dopaminergic neurons after treatment with NCGC607	2
GBA1	394	Increased levels of lysosomal GlcSph in dopaminergic neurons	2
VPS13A	395	Increased neurite outgrowth in GABAergic medium spiny neurons	2
VPS13A	396	Increased neurite ramification in GABAergic medium spiny neurons	2
VPS13A	397	Increased Na+ current amplitudes in medium spiny neurons	2
VPS13A	398	Increased amplitude of provoked action potentials in medium spiny neurons	2
VPS13A	399	Increased amplitude of spontaneous action potentials in medium spiny neurons	2
VPS13A	400	Increased amplitude of miniature postsynaptic currents in medium spiny neurons	2
VPS13A	401	Increased percentage of medium spiny neurons showing miniature postsynaptic currents	2
VPS13A	402	Increased G/F-actin ration in medium spiny neurons	2

VPS13A	403	Increased phosphorylation of cofflin in medium spiny neurons	2
NPC1	404	Increased co-localization of cholesterol/ GM2 in neurons	2
NPC1	405	Increased accumulation of osmiophilic material in neurons	2
NPC1	406	Increased levels of GM2 in neurons	2
NPC1	407	Increased levels of Hex A mRNA in neurons	1
NPC1	408	Increased protein levels of Hex A in neurons	2
MAPT	409	Increased levels of exon-10 containing *4R-TAU* isoforms in astrocytes	1
MAPT	410	Increased levels of exon-10 containing 4R-TAU isoforms in neurons	1
MAPT	411	Increased astrocyte size	1
MAPT	412	Increased levels of ubiquinated proteins in astrocytes	1
MAPT	413	Increased cell death in astrocytes after exposure to rotenone	1
MAPT	414	Increased release of lactate dehydrogenase in astrocytes after exposure to rotenone	1
MAPT	415	Increased protein levels of ANXA2 in astrocytes	1
DISC1	416	Increased total dendritic length in neurons at one and two weeks post-differentiation	1
PSEN1	417	Decreased amount of extracellular Aβ 42 by neurons by treatment with a selective Aβ42-lowering agent	1
PSEN2	417	Decreased amount of extracellular Aβ 42 by neurons by treatment with a selective Aβ42-lowering agent	1
PSEN1	418	Decreased amount of extracellular Aβ 42 by neurons after treatment with γ-secretase inhibitor	1
PSEN2	418	Decreased amount of extracellular Aβ 42 by neurons after treatment with γ-secretase inhibitor	1
LRRK2	419	Decreased amount of dopaminergic neurons after exposure to valinomycin	1
PINK1	419	Decreased amount of dopaminergic neurons after exposure to valinomycin	1
LRRK2	420	Decreased amount of dopaminergic neurons after exposure to concanamycin A	1
PINK1	420	Decreased amount of dopaminergic neurons after exposure to concanamycin A	1
APP	421	Decreased ROS in neurons after treatment with β-secretase inhibitor	1
Sporadic (AD)	421	Decreased ROS in neurons after treatment with β-secretase inhibitor	1
Parkin	422	Decreased binding of CFT in neurons	1
TDP-43	423	Decrease in size of neurites	3
Parkin	423	Decrease in size of neurites	1
MAPT	423	Decrease in size of neurites	3
LRRK2	424	Decreased amount of TUJ1+ NSCs	1
MeCP2	424	Decreased amount of TUJ1+ NSCs	1

ABCD1	425	Decreased C26:0/C22:0 ratio levels in oligodendrocytes by lovastatin (through induced upregulation of ABCD2 gene)	1
ALDP	425	Decreased C26:0/C22:0 ratio levels in oligodendrocytes by lovastatin (through induced upregulation of ABCD2 gene)	1
ABCD1	426	Decreased C26:0/C22:0 ratio levels in oligodendrocytes after treatment with 4-PBA (through induced upregulation of ABCD2 gene)	1
ALDP	426	Decreased C26:0/C22:0 ratio levels in oligodendrocytes after treatment with 4-PBA (through induced upregulation of ABCD2 gene)	1
APP	427	Decreased amount of Aβ-oligomers in neurons with β-secretase inhibitor treatment	1
Sporadic (AD)	427	Decreased amount of Aβ-oligomers in neurons with β-secretase inhibitor treatment	1
APP	428	Decreased amount of oxidative stress in neurons after treatment with β-secretase inhibitor	1
APP	429	Decreased ratio of secreted APPs-α to APPs-β	1
APP	430	Decreased tau protein levels in neurons after treatment with 3D6 antibody	1
APP	431	Decreased Tau protein levels in neurons after treatment with AW7 antibody	1
APP	432	Decreased p-tau/total tau ratio in neurons after treatment with β-secretase inhibitors	1
APP	433	Decreased anti-glycogen synthase kinase 3 beta (aGSK-3β) levels in neurons after treatment with β-secretase inhibitors	1
PSEN1	434	Decreased secretion levels of Aβ 40 and 42 in neurons after treatment with Dual Antiplatelet Therapy (DAPT)	1
PSEN1	435	Decreased production of endogenous Aβ 40 in neurons	1
LRRK2	436	Decreased amount of basal autophagy in neurons	1
GBA1	437	Decreased amount of autophagic flux in neurons	1
GBA1	438	Decreased activity of GBA2 in neurons	1
GBA1	439	Decreased activity of β-galactosidase in neurons	1
LRRK2	440	Decreased average neurite length of DA neurons after exposure to rapamycin	1
LRRK2	441	Decreased average neurite length of DA neurons after exposure to leupeptin	1
LRRK2	442	Decrease in mitochondrion length in the proximal axon of neurons	1
LRRK2	443	Decreased levels of co-localized LC3/LAMP-1 in neurons	1
LRRK2	444	Decreased pERK levels in neurons after treatment with LRRK2-IN1	1
LRRK2	445	Decreased neuronal oxidative-stress induced cytotoxicity with MEK kinase inhibitor treatment	1
LRRK2	446	Decreased degeneration of neurons after treatment with MEK kinase inhibitor	1

LRRK2	447	Decreased clonal expansion in NSCs	1
LRRK2	448	Decreased phosphorylation of LRRK2 downstream targets in NSCs after treatment with LRRK2-In-1	1
SNCA	449	Decreased protein expression levels of NURR1 and TH in neurons	1
SNCA	450	Decreased accumulation of ER-associated degradation substrates (Gcase, Nicastrin) in neurons after treatment with NAB2	1
SNCA	451	Decreased levels of ER stress in neurons after treatment with NAB2	1
SNCA	452	Decreased levels of nitrative stress in neurons after treatment with NAB2	1
Parkin	453	Decreased dopamine reuptake by neurons	1
Parkin	454	Decreased amount of DAT-binding sites on neurons	1
PINK1	455	Decreased recruitment of Parkin to mitochondria in neurons	1
PINK1	456	Decreased mROS production in neurons exposed to low concentrations of valinomycin after treatment with rapamycin	1
PINK1	457	Decreased amount of outer mitochondrial membrane (OMM) proteins of increased molecular mass in neurons	1
PINK1	458	Decrease production of mROS in neurons in response to valinomycin after treatment with LRRK2 inhibitor GW5074	1
PINK1	459	Decreased levels of glutathione in neurons after exposure to valinomycin	1
PINK1	460	Decreased levels of glutathione in neurons after exposure to concanamycin A	1
PINK1	461	Decreased levels of glutathione in neurons after exposure to MPP+	1
PINK1	462	Decreased levels of glutathione in neurons after exposure to hydrogen peroxide	1
SMN1	463	Decreased neuromuscular junction size of neurons	1
SMN1	464	Decrease in growth cone size in neurons	1
SMN1	465	Decrease in mean length of axons in neurons	1
SMN1	466	Decrease in SMN protein in neurons	1
SMN1	467	Decreased capacity to generate motor neurons	1
SMN1	468	Decreased amount of total cell body volume in neurons	1
SMN1	469	Decreased amount of cellular processes from neurons	1
SMN1	470	Decreased amount of SMI-32+ neurons at time of late differentiation in neurons	1
SMN1	471	Decreased size of motor neurons	1
SMN1	472	Decreased levels of SMN protein in astrocytes	1
MeCP2	473	Decrease in frequency/ amplitude of spontaneous postsynaptic currents of neurons	1
MeCP2	474	Decreased amount of neuronal synapses	1
MeCP2	475	Decreased amount of the density of V-GLUT1 puncta in dendrites of neurons	1
MeCP2	476	Decreased soma size of glutamatergic neurons	1
MeCP2	477	Decreased spine density in glutamatergic neurons	1

MeCP2	478	Decreased formation of TuJ+ iPSCs	1
CDKL5	479	Decreased amount of synaptic contacts in neurons	1
ATXN3	480	Decreased ATXN3 aggregate formation in neurons after treatment with calpeptin (calpain inhibitor)	1
ATXN3	481	Decreased ATXN3 aggregate formation in neurons after treatment with ALLN (calpain inhibitor)	1
PGRN	482	Decreased S6K2 protein levels in neurons	1
PGRN	483	Decreased levels of intracellular progranulin in neurons	1
SNCA	484	Decreased levels of SNCG expression in neurons	1
PGRN	485	Decreased caspase-3 activity in neurons after ectopic progranulin expression	1
C9ORF72	486	Decreased electrical excitability of neurons	1
C9ORF72	487	Decreased spike size upon depolarization of neurons	1
C9ORF72	488	Decreased formation of RNA foci in neurons after treatment with antisense oligonucleotides	1
C9ORF72	489	Decreased expression of C9ORF72 in neurons	1
TDP-43	490	Decreased survival of neurons	1
TDP-43	491	Decreased amount of TDP-43 insoluble fractions in neurons after exposure to anacardic acid	1
SOD1	492	Decreased neurofilament aggregation in motor neurons by induction of exogenous NF-L after treatment with Dox (doxycycline)	1
SOD1	493	Decreased neurite degeneration in motor neurons by induction of exogenous NF-L after treatment with Dox (doxycycline)	1
CACNA1C	494	Decreased amount of neurons expressing lower layer markers (FOXP1 and ETV1)	1
CACNA1C	495	Decreased proportion of SATB2 expressing neurons in lower layer marker (FOXP1 and ETV1) expressing neurons	1
CACNA1C	496	Decreased percent of neurons expressing TH+ after treatment with roscovitine	1
CACNA1C	497	Decrease in sustained calcium ion rise following depolarization of neurons after treatment with nimodipine	1
HTT	498	Decreased mitochondrial membrane potential in medium spiny neurons	1
HTT	499	Decreased levels of ATP in medium spiny neurons	1
HTT	500	Decreased mitochondrial fragmentation along neurites in GABA neurons after treatment with dynamin-related protein 1 peptide inhibitor P110-TAT	1
HTT	501	Decreased mROS formation in medium spiny neurons after treatment with dynamin-related protein 1 peptide inhibitor P110-TAT	1
HTT	502	Decreased levels of mitochondrial associated dynamin-related protein 1 after treatment with dynamin-related protein 1 peptide inhibitor P110-TAT in neurons	1
HTT	503	Decreased neuronal death upon BDNF withdrawal after treatment	1

		with dynamin-related protein 1 peptide inhibitor P110-TAT in neurons	
HTT	504	Decreased levels of mitochondrial associated p53 after treatment with dynamin-related protein 1 peptide inhibitor P110-TAT in neurons	1
HTT	505	Decreased binding of phalloidin-peptide to actin cytoskeleton in NSCs	1
HTT	506	Decreased cell-cell adhesion properties in NSCs	1
HTT	507	Decreased intracellular ATP (energy metabolism compromised) in NSCs	1
HTT	508	Decrease in average Oxygen Consumption Rate (OCR) in NSCs after exposure to Carbonyl cyanide-p-trifluoromethoxypheny (FCCP)	1
HTT	509	Decreased ATP/ADP ratios (energy metabolism compromisation) in NSCs	1
HTT	510	Decreased protein levels of N-cadherin in NSCs	1
HTT	511	Decreased TGF-β1 mRNA in NSCs	1
HTT	512	Decreased expression of cytoskeleton-associated proteins in iPSCs	1
HTT	513	Decreased neuronal differentiation in iPSCs	1
HTT	514	Decreased rosette forming efficiency in iPSCs	1
HTT	515	Decreased levels of representative antioxidant molecules (Superoxide dismutase1, Glutathione transferase, and Glutathione peroxidase 1) in iPSCs	1
SCN1A	516	Decreased threshold for action potential generation in neurons	1
SCN1A	517	Decrease in action potentials in marked amplitude attenuation in GABAergic neurons	1
SCN1A	518	Decreased amount of action potential firing in GABAergic neurons	1
SCN1A	519	Decreased output capacity of GABAergic neurons during intense stimulation	1
FXN	520	Decreased excitability of neurons	1
FXN	521	Decreased frataxin levels in iPSCs	1
FXN	522	Decreased mitochondrial membrane potential in iPSCs	1
ERCC6	523	Decreased growth rate in iPSCs	1
FMR1	524	Decreased neurite outgrowth by forebrain neurons	1
FMR1	525	Decrease in length/number of processes extended by forebrain neurons	1
FMR1	526	Decreased motility/slow rate of neurite extension in forebrain neurons	1
ATP7A	527	Decreased expression of ATP7A in iPSCs	1
ATP7A	528	Decreased expression of NESTIN and aberrant rosette lumens during neurosphere development in iPSCs	1
ATP7A	529	Decreased number of N-cad+/ Sox2+ neural rosettes in iPSCs	1
SOD1	530	Decreased amount of motor neurons	1

SMN1	530	Decreased amount of motor neurons	1
APP	531	Decreased amount of Aβ oligomers in astrocytes after treatment with β-secretase inhibitor	1
Sporadic (AD)	531	Decreased amount of Aβ oligomers in astrocytes after treatment with β-secretase inhibitor	1
GAA	532	Decreased amount of glycogen granules after treatment with acid-α-glucosidase in iPSCs	1
NPC1	533	Decrease in autophagic clearance in neurons (increased p62 and LC3-II levels)	1
GBA1	534	Decrease in proteolysis of long-lived proteins (α-synuclein, Htt, Tau) in neurons	1
SMN1	535	Decreased motor neuron survival	1
SOD1	535	Decreased motor neuron survival	1
hnRNPA2B1	535	Decreased motor neuron survival	1
SMN1	536	Decreased neuron size	1
HTT	537	Decreased neurite outgrowth in neurons	1
SMN1	537	Decreased neurite outgrowth in neurons	1
LRRK2	537	Decreased neurite outgrowth in neurons	1
APP	538	Decrease in ROS in astrocytes after treatment with β-secretase inhibitor	1
Sporadic (AD)	538	Decrease in ROS in astrocytes after treatment with β-secretase inhibitor	1
SNCA	539	Decreased ratio of post ER-to-ER forms in neurons	1
PLP1	540	Decreased frequency of myelin formation in oligodendrocytes	1
PLP1	541	Decreased thickness of myelin sheaths in oligodendrocytes	1
LRRK2	542	Decreased Basal Oxygen Consumption Rate (OCR) in neurons	2
PANK2	542	Decreased Basal Oxygen Consumption Rate (OCR) in neurons	2
PGRN	543	Decreased levels of secreted progranulin in neurons	1
GBA1	544	Decreased glucocerebrosidase enzymatic activity in neurons	1
CACNA1C	545	Decrease in dendritic length upon depolarization in neurons	1
PSEN1	546	Decreased amount of Aβ42 in neurons after treatment with γ-secretase modulator	1
PSEN1	547	Decreased amount of Aβ40 in neurons after treatment with γ-secretase modulator	1
PSEN1	548	Decreased amount of Aβ38 in neurons after treatment with γ-secretase modulator	1
PSEN1	549	Decreased Aβ42:Aβ40 ratio in neurons after treatment with γ-secretase modulator	1
PSEN1	550	Decreased amount of extracellular Aβ 42 by neurons after treatment with γ-secretase modulator	1
PSEN1	551	Decreased amount of extracellular Aβ 40 by neurons after treatment with γ-secretase modulator	1
PSEN1	552	Decreased amount of Aβ 42 in neurons after treatment with γ-	1

		secretase inhibitor	
PSEN1	553	Decreased amount of Aβ 40 in neurons after treatment with γ-secretase inhibitor	1
PSEN1	554	Decreased amount of Aβ 38 in neurons after treatment with γ-secretase inhibitor	1
PSEN1	555	Decreased amount of total Aβ in neurons after treatment with γ-secretase inhibitor	1
PSEN1	556	Decreased amount of Aβ42 in iPSCs after treatment with γ-secretase modulator	1
PSEN1	557	Decreased amount of Aβ40 in iPSCs after treatment with γ-secretase modulator	1
PSEN1	558	Decreased amount of Aβ38 in iPSCs after treatment with γ-secretase modulator	1
PSEN1	559	Decreased Aβ42:Aβ40 ratio in iPSCs after treatment with γ-secretase modulator	1
PSEN1	560	Decreased Aβ42:Aβ40 ratio in NSCs after treatment with γ-secretase modulator	1
PSEN1	561	Decreased amount of Aβ42 in NSCs after treatment with γ-secretase modulator	1
APP	561	Decreased amount of Aβ42 in NSCs after treatment with γ-secretase modulator	1
PSEN1	562	Decreased amount of Aβ40 in NSCs after treatment with γ-secretase modulator	1
APP	562	Decreased amount of Aβ40 in NSCs after treatment with γ-secretase modulator	1
PSEN1	563	Decreased amount of Aβ38 in NSCs after treatment with γ-secretase modulator	1
APP	563	Decreased amount of Aβ38 in NSCs after treatment with γ-secretase modulator	1
Parkin	564	Decreased number of terminals in neurons	1
Parkin	565	Decreased number of branch points in neurons	1
MAPT	566	Decreased neurite complexity in neurons	1
Parkin	566	Decreased neurite complexity in neurons	1
Parkin	567	Decreased microtubule stability in neurons	1
Parkin	568	Decrease in polymerized tubulin in pellet fractions in neurons	1
Parkin	569	Decreased neurite length in neurons after treatment with colchicine	1
Parkin	570	Decreased number of terminals in neurons after treatment with colchicine	1
Parkin	571	Decreased number of branch points in neurons after treatment with colchicine	1
Parkin	572	Decreased neurite complexity in neurons after treatment with colchicine	1
C9ORF72	573	Decreased action potential output in motor neurons	1

TDP-43	573	Decreased action potential output in motor neurons	1
C9ORF72	574	Decreased synaptic input in motor neurons	1
TDP-43	574	Decreased synaptic input in motor neurons	1
C9ORF72	575	Decrease Na+ currents in motor neurons	1
TDP-43	575	Decrease Na+ currents in motor neurons	1
C9ORF72	576	Decreased K+ currents in motor neurons	1
TDP-43	576	Decreased K+ currents in motor neurons	1
C9ORF72	577	Decreased synaptic activity in motor neurons	1
TDP-43	577	Decreased synaptic activity in motor neurons	1
SMN1	578	Decrease in Na+ channel inactivation recovery time in motor neurons	1
MeCP2	579	Decreased nuclear size in neurons	1
SHANK3	580	Decreased production of neurons in iPSCs	1
SHANK3	581	Decreased amplitude of spontaneous excitatory post-synaptic currents in neurons	1
SHANK3	582	Decreased frequency of spontaneous excitatory post-synaptic currents in neurons	2
SHANK3	583	Decreased amplitude of spontaneous synaptic events in neurons	1
SHANK3	584	Decreased frequency of spontaneous synaptic events in neurons	1
SHANK3	585	Decreased current size generation in response to focal application of AMPA in neurons	1
SHANK3	586	Decreased current size generation in response to focal application of NMDA in neurons	1
SHANK3	587	Decreased amount of excitatory synapses in neurons	1
SHANK3	588	Decreased input resistance after treatment with IGF1 in neurons	1
APP	589	Decreased cell viability in neurons	1
APP	590	Decreased levels of binding protein (BiP) in neurons after treatment with DHA	1
Sporadic (AD)	590	Decreased levels of binding protein (BiP) in neurons after treatment with DHA	1
APP	591	Decreased levels of ROS in neurons after treatment with DHA	1
APP	592	Decreased levels of cleaved caspase-4 in neurons after treatment with DHA	1
APP	593	Decreased levels of binding protein (BiP) in neurons after treatment with β-secretase inhibitor	1
Sporadic (AD)	593	Decreased levels of binding protein (BiP) in neurons after treatment with β-secretase inhibitor	1
APP	594	Decreased levels of cleaved caspase-4 in neurons after treatment with β-secretase inhibitor	1
GBA1	595	Decreased protein levels of Glucocerebrosidase (Gcase) in neurons	1
GBA1	596	Decreased enzymatic activity of Glucocerebrosidase (Gcase) in neurons	1
CACNA1C	597	Decrease in sustained calcium ion rise following depolarization of	1

		NSCs after treatment with nimodipine	
APP	598	Decreased levels of binding protein (BiP) in astrocytes after treatment with β-secretase inhibitor	1
Sporadic (AD)	598	Decreased levels of binding protein (BiP) in astrocytes after treatment with β-secretase inhibitor	1
APP	599	Decreased levels of cleaved caspase-4 in astrocytes after treatment with β-secretase inhibitor	1
HTT	600	Decreased levels of mitochondrial associated dynamin-related protein 1 after treatment with dynamin-related protein 1 peptide inhibitor P110-TAT in iPSCs	1
HTT	601	Decreased levels of mitochondrial associated p53 after treatment with dynamin-related protein 1 peptide inhibitor P110-TAT in iPSCs	1
APP	602	Decreased levels of extracellular Aβ42 in neurons	1
APP	603	Decreased levels of extracellular Aβ40 in neurons	1
SOD1	604	Decreased soma size of motor neurons	1
SOD1	605	Decrease in length/number of processes in motor neurons	1
SOD1	606	Decreased levels of SDHA protein in motor neurons	1
SOD1	607	Decreased levels of MT-COX1 protein in motor neurons	1
SOD1	608	Decreased amount of motile mitochondria in motor neurons	1
SOD1	609	Decreased survival of motor neurons when cultured with control glia	1
FMR1	610	Decrease in length/number of processes in neurons	1
DISC1	611	Decreased frequency of excitatory spontaneous synaptic currents in neurons	1
DISC1	612	Decreased density of SV2+ synaptic boutons in neurons	1
DISC1	613	Decreased depolarization-induced vesicle release in neurons	1
HTT	614	Decreased neurite-like processes length in neurons after treatment with isoxazole-9	3
HTT	615	Decreased nuclear:cytoplasmic ratio of endogenous Ran in neurons	2
HTT	616	Decreased nuclear:cytoplasmic ratio of endogenous RanGAP1 in neurons	2
HTT	617	Decreased nuclear:cytoplasmic ratio of endogenous NUP62 in neurons	2
C9ORF72	618	Decreased velocity of actin movement in axonal growth cones of motor neurons	1
DHCR7	619	Decreased cholesterol levels in iPSCs after exposure to cholesterol-deficient mTesR1 medium	3
DHCR7	620	Decreased rosette formation in iPSCs	2
DHCR7	621	Decreased formation of secondary filaments in iPSCs cultured in cholesterol-deficient mTesR1 medium after treatment with LDL	1
HTT	622	Decreased medium spiny neuronal death after treatment with KD3010 (PPAR-δ agonist)	2
hnRNPA2B1	623	Decreased SRSF7 levels in motor neurons after treatment with an ASO targeting Hnrnpa2b1	1

VCP	623	Decreased SRSF7 levels in motor neurons after treatment with an ASO targeting Hnrnpa2b1	1
PANK2	624	Decreased average maximal firing rate in neurons	2
PANK2	625	Decreased peak amplitudes of voltage-dependent Na+ currents in neurons	2
PANK2	626	Decreased mitochondrial membrane potential in neurons	2
PANK2	627	Decreased glutathione levels in neurons	2
PANK2	628	Decreased aconitase activity in neurons	2
PANK2	629	Decreased heme levels in NSCs	2
PANK2	630	Decreased ferritin levels in neurons	2
PANK2	631	Decreased loss of neurons after treatment with CoA	2
PANK2	632	Decreased ROS levels in neurons after treatment with CoA	2
MAPT	633	Decreased cell viability of neurons after exposure to piericidin A	1
MAPT	634	Decreased cell viability of neurons after exposure to glutamate	1
MAPT	635	Decreased cell viability of neurons after exposure to NMDA	1
MAPT	636	Decreased cell viability of neurons after exposure to MG132	1
MAPT	637	Decreased cell viability of neurons after exposure to epoxomicin	1
MAPT	638	Decreased cell viability of neurons after exposure to Aβ(1–42)	1
GBA1	639	Decreased protein levels of Glucocerebrosidase (Gcase) in NSCs	2
GBA1	640	Decreased activity levels of Glucocerebrosidase (Gcase) in NSCs	2
GBA1	641	Decreased protein levels of Glucocerebrosidase (Gcase) in dopaminergic neurons	2
GBA1	642	Decreased activity levels of Glucocerebrosidase (Gcase) in dopaminergic neurons	2
GBA1	643	Decreased intracellular levels of dopamine in dopaminergic neurons	2
GBA1	644	Decreased levels of DAT1 mRNA in dopaminergic neurons	1
GBA1	645	Decreased levels of VMAT2 mRNA in dopaminergic neurons	1
GBA1	646	Decreased dopamine reuptake in dopaminergic neurons	1
GBA1	647	Decreased levels of lysosomal GlcSph in dopaminergic neurons after treatment with NCGC607	1
GBA1	648	Decreased levels of α-synuclein in dopaminergic neurons after treatment with NCGC607	1
GBA1	649	Decreased levels of colocalized α-synuclein/Lamp2 in dopaminergic neurons after treatment with NCGC607	1
VPS13A	650	Decreased voltage dependence of Na+ channel activation in medium spiny neurons	2
VPS13A	651	Decreased frequency of synaptic currents in medium spiny neurons after treatment with phallacadin	2
VPS13A	652	Decreased amplitude of synaptic currents in medium spiny neurons after treatment with phallacadin	2
VPS13A	653	Decreased amplitude of action potentials in medium spiny neurons after treatment with phallacadin	2
VPS13A	654	Decreased frequency of synaptic currents in medium spiny neurons	2

		after treatment with PP2 (Src kinase inhibitor)	
VPS13A	655	Decreased amplitude of synaptic currents in medium spiny neurons after treatment with PP2 (Src kinase inhibitor)	2
VPS13A	656	Decreased amplitude of action potentials in medium spiny neurons after treatment with PP2 (Src kinase inhibitor)	2
VPS13A	657	Decreased number of medium spiny neurons at time of late differentiation	2
NPC1	658	Decreased levels of GM3 in neurons	2
NPC1	659	Decreased Hex A enzymatic activity in neurons	2
MAPT	660	Decreased nucleus/cytoplasm ratio in astrocytes	1
ERCC6	661	Decreased synaptic density in neurons	1
ERCC6	662	Decreased action potential spike number in neurons	1
ERCC6	663	Decreased number of synchronized events in neurons	1

Appendix Table S11. **Localization of Gene Expression in Human Brain of Dysregulated Genes from GEO Datasets**

Gene	Disease	Cell Type	Defect		Localization of Gene Expression in Adult Human Brain		Localization of Gene Expression in Prenatal Human Brain	
			log(FC)	*P*-Value	Up	Down	Up	Down
AASS	Friedreich's Ataxia	iPSCs	2.04	1.55E-05	CbN, CC, GP, MY, VT	CbCx, Cx, DG	SVZ	CP
COL1A1	Friedreich's Ataxia	iPSCs	-2.09	5.17E-04	CGS, DT, LV, Str	CbCx, Cx, HTM	SVZ	CP
DLK1	Friedreich's Ataxia	iPSCs	-2.95	6.76E-04	HTM, MTg, MY, Pons	Cx, DT, LV		
FABP5	Friedreich's Ataxia	iPSCs	2.35	7.55E-06	CGS, ET, LV	Cl, Cx, DT	SVZ	CP
FLRT3	Friedreich's Ataxia	iPSCs	-2.29	2.08E-02	CbCx, Cx, DG	AMG, BG, MTg, MY, Pons		IZ
FOXA2	Friedreich's Ataxia	iPSCs	-2.15	4.06E-02	CbN, CC, GP, MY, Pons, VT	CbCx, ET, HPC	CP	SVZ
GDF15	Friedreich's Ataxia	iPSCs	-2.73	1.16E-06	LV	Cx	IZ	SVZ
IER3	Friedreich's Ataxia	iPSCs	-2.17	7.55E-05	LV, Str	CbCx, DG, DT	CP	SVZ
IGFBP6	Friedreich's Ataxia	iPSCs	-2.13	1.93E-03	CbN, Pons	AMG, CbCx, CC, HPC		SVZ
IGFBP7	Friedreich's Ataxia	iPSCs	-2.02	1.53E-04	CbN, BG, MY, LV	AMG, CbCx, HPC	IZ	CP
KIAA1551	Friedreich's Ataxia	iPSCs	2.09	4.16E-06	CbCx, CC, GP	HPC, HTM, MTg, Pons		
PHLDA1	Friedreich's Ataxia	iPSCs	-2.40	8.43E-06	MTg, Pons, Str, VT	CbCx, CGS, HPC, LV	SVZ	CP
PROS1	Friedreich's Ataxia	iPSCs	-2.78	5.59E-04	CGS, HPC, LV	CbCx	SVZ	CP
TDGF1	Friedreich's Ataxia	iPSCs	2.25	7.01E-06	CGS, DT, Pons, VT	AMG, HTM, LV	IZ	CP
TM4SF18	Friedreich's Ataxia	iPSCs	-2.07	2.33E-03	CbN, DT, MY	CbCx, CGS, DG	IZ	CP
TTR	Friedreich's Ataxia	iPSCs	-2.80	1.44E-02	CC, Str	CbN, Cx	SVZ	CP
TXNL1	Friedreich's Ataxia	iPSCs	2.01	3.83E-06	HPC, MY, Pons	CbCx, CxN	CP	
WDR11	Friedreich's Ataxia	iPSCs	2.20	1.78E-05	CbCx, CGS	AMG, CbN, HPC, MTg		IZ
ZFP42	Friedreich's Ataxia	iPSCs	3.22	6.96E-03	MY	HTM	SVZ	
BNIP3	Cockayne Syndrome	iPSCs	2.05	1.29E-08	DT, HPC, MY	BG, CbCx, HTM, LV	IZ	SVZ
CST1	Cockayne Syndrome	iPSCs	3.41	1.67E-04	AMG, MTg, Pons	CGS, DG, HTM, LV	IZ	SVZ
CXCR4	Cockayne Syndrome	iPSCs	2.12	1.52E-08	CbN, CC, GP, MTg, MY	CbCx, Cx, HPC	SVZ	CP
CYP26A1	Cockayne Syndrome	iPSCs	2.11	2.29E-07	Cx	CbCx, DG, HTM, MTg, TH	CP, IZ	SVZ
GCNT4	Cockayne Syndrome	iPSCs	-2.83	4.05E-08	Cl, Cx	Cb, HPC, HTM, MTg, TH	SVZ	
NLRP2	Cockayne Syndrome	iPSCs	3.38	1.25E-09	CbN, HTM, PL	CbCx, CC, GP	CP	SVZ
PRTG	Cockayne Syndrome	iPSCs	2.57	1.11E-07	CbN, HTM, MTg, MY, Pons	CbCx, Cx, DG, Str	IZ	SVZ
TXNIP	Cockayne Syndrome	iPSCs	2.42	1.09E-04	CC, GP, MTg, MY, VT	HPC, HTM	SVZ	
WLS	Cockayne Syndrome	iPSCs	2.01	6.80E-06	CGS, DT, HTM, LV	CbCx, DG	SVZ	CP
ABL1	Parkinson's	iPSCs	-2.05	4.98E-02	BG, CC, TH	Cb	IZ	
BNC1	Parkinson's	iPSCs	4.23	3.14E-02		CGS, DT, HTM, LV		
CCNL2	Parkinson's	iPSCs	-3.66	1.86E-02	CbCx, CC, LV	HTM, MTg, Pons	CP	SVZ
CHRNA7	Parkinson's	iPSCs	2.40	1.46E-02	HTM, Pons, VT	CbCx, CC		SVZ
CPNE4	Parkinson's	iPSCs	-2.09	1.37E-02	Cl, DT, HPC	CbCx, CC, LV	CP	IZ, SVZ
CRMP1	Parkinson's	iPSCs	-2.26	4.13E-02	AMG, HPC	CbN, CC, CGS, GP, LV	CP	SVZ
DLGAP2	Parkinson's	iPSCs	-3.29	2.72E-02	BG, Cx, HPC	CbCx, CC, DT, LV	CP	SVZ
DLL1	Parkinson's	iPSCs	2.32	1.30E-02	BG, DT, MY	Cb, DG	SVZ	CP
FOXC1	Parkinson's	iPSCs	3.10	1.06E-02	Pons, MY, LV, TH	Cb, DG		CP
G0S2	Parkinson's	iPSCs	-2.33	3.86E-02	BG, CbN, MTg	CC, HPC	IZ	SVZ
GLI2	Parkinson's	iPSCs	2.99	1.60E-02	AMG, BG, CGS	Cb, HPC	SVZ	CP
GREM1	Parkinson's	iPSCs	2.48	1.68E-04	CC, GP, VT	Cb, DG, HTM		SVZ
GRIK2	Parkinson's	iPSCs	-5.07	2.02E-02	AMG, CbCx, DG	CbN, CC, DT, GP, MTg		SVZ
GRIN2B	Parkinson's	iPSCs	-2.56	3.46E-02	Cx, HPC, TH	Cb, CC, HTM, MTg, Pons	CP, IZ	SVZ

GRIN3B	Parkinson's	iPSCs	-2.15	3.87E-02	CC, GP	AMG, MTg	CP	SVZ
HES1	Parkinson's	iPSCs	2.41	1.30E-02	BG, CC, DT	Pons	SVZ	CP
HES5	Parkinson's	iPSCs	2.90	1.20E-02	AMG, HTM, Str	CGS, CbN, DG, LV, Pons	SVZ	CP, IZ
HOXA2	Parkinson's	iPSCs	2.11	1.38E-02	MY, Pons	CC, LV		
HOXA9	Parkinson's	iPSCs	-2.70	2.76E-05	CGS, MTg, MY	CC, DG, DTH, FL	IZ	
HOXB5	Parkinson's	iPSCs	-2.26	1.40E-02	HTM, MY, Pons	Cb, DTH, FL		SVZ
HOXC8	Parkinson's	iPSCs	-5.80	1.37E-02	AMG, CbN, HTM, MY	CGS, BG, DG	IZ	SVZ
HSPA1A	Parkinson's	iPSCs	-2.85	1.30E-02	CbN, CC, GP, MTg, VT	AMG, CbCx, Cx, HPC	SVZ	CP
KCNC1	Parkinson's	iPSCs	-2.30	3.34E-02	CbCx, DT, GP, OL, PL	AMG, CC, HPC, HTM, MY		SVZ
LFNG	Parkinson's	iPSCs	2.61	1.64E-02	GP, MTg, MY	HPC, OL, PL, TL	SVZ	CP
LMO3	Parkinson's	iPSCs	-3.88	2.67E-02	AMG, BG, CI, Cx, DG	CbCx, HTM, LV, TH	CP	SVZ
MT3	Parkinson's	iPSCs	-2.07	2.17E-02	AMG, Cx, GP, MTg	CbCx, CGS, HPC, HTM, LV		CP
NEB	Parkinson's	iPSCs	-2.12	2.70E-02	CGS, BG, HTM	AMG, HPC, MTg, Pons		
NHLH2	Parkinson's	iPSCs	-3.64	1.30E-02	CbCx, DT, HPC, Pons	CbN, CC, Cx, LV		SVZ
NOTCH2	Parkinson's	iPSCs	5.30	3.41E-02	BG, CGS, CxN	Cb	SVZ	CP
ONECUT1	Parkinson's	iPSCs	-2.20	3.44E-02	GP, MTg, MY, Pons	CC, Cx, DT, LV, Str		SVZ
PARM1	Parkinson's	iPSCs	2.54	1.38E-02	Cx, DG, Pons	Cb, CC	IZ	SVZ
PDGFD	Parkinson's	iPSCs	2.43	1.72E-02	HTM, MTg, Pons	CC, Cb, TH	SVZ	CP
PER2	Parkinson's	iPSCs	2.08	1.82E-02	CbCx, Cx	DT, HTM, MTg, MY, Pons	IZ	CP
POSTN	Parkinson's	iPSCs	2.33	1.74E-02	AMG, DG	TH	IZ	SVZ
PPP1R8	Parkinson's	iPSCs	-3.33	2.85E-02	GP, MTg, MY, VT	CbCx, Cx, HPC	CP, SVZ	IZ
ROR1	Parkinson's	iPSCs	2.49	1.62E-02	Cb	HPC	SVZ	CP
RUNX3	Parkinson's	iPSCs	2.13	1.58E-02	AMG, MY, VT	Cb, HTM	IZ	CP, SVZ
SNAI2	Parkinson's	iPSCs	2.23	4.85E-02	Cb	HTM	CP	SVZ
SOSTDC1	Parkinson's	iPSCs	2.31	1.96E-02	Cx, LV	Cb, DT, HTM, MY, Str	CP	SVZ
TGFB3	Parkinson's	iPSCs	2.81	1.40E-02	CC, GP, VT	HPC, HTM		CP
TWIST2	Parkinson's	iPSCs	2.81	1.42E-02	AMG, FL, MY	BG, Cb, DG		SVZ
VSNL1	Parkinson's	iPSCs	-4.38	1.30E-02		CC, CxN		SVZ
WWOX	Parkinson's	iPSCs	-2.48	4.28E-02	Cb, CGS	HPC	SVZ	CP, IZ
ZEB1	Parkinson's	iPSCs	2.15	1.18E-02	CGS, DT, MY, Pons	Cb, CI, HPC	IZ, SVZ	CP
ZEB2	Parkinson's	iPSCs	2.55	1.72E-02	CC, GP, HPC, MY, VT	Cb, HTM, LV	CP, IZ	
ZIC2	Parkinson's	iPSCs	2.46	1.37E-02	CbCx, DT, LV	Cx, DG, HTM, MTg, Pons		CP
ZNF254	Parkinson's	iPSCs	4.04	1.93E-02	AMG, CbCx, DG	CbN, Cx, HTM, Pons		SVZ
AGPS	Parkinson's	Neurons	2.06	1.95E-02	MTg, VT	BG, HPC		SVZ
SUV39H1	Parkinson's	Neurons	-2.29	1.95E-02	Cb, FL	MY, LV	SVZ	CP
ACTC1	Parkinson's	NSCs	-2.323	3.28E-05	HPC	Cb	SVZ	CP
ADM	Parkinson's	NSCs	-2.129	3.36E-06	MY, Pons	Cb	IZ	CP, SVZ
ANKRD1	Parkinson's	NSCs	-2.98	1.89E-06			CP	SVZ
ANXA3	Parkinson's	NSCs	-3.407	1.58E-06	HTM, Pons	AMG, Cb, HPC	SVZ	CP, IZ
BDNF	Parkinson's	NSCs	-2.194	4.04E-05	CbCx, DG	CC, GP	IZ	SVZ
CAV2	Parkinson's	NSCs	-2.569	5.58E-06	CbN, DT	CbCx, DG	CP	IZ, SVZ
CDH6	Parkinson's	NSCs	-2.843	1.58E-06	CbCx, DG	DT, GP	CP	IZ
COL1A2	Parkinson's	NSCs	2.999	1.49E-06	CbN, CGS, HTM	CbCx, Cx		CP
COLEC12	Parkinson's	NSCs	-2.216	4.70E-06	CbN, CGS, LV, MTg	Cx, CbCx, DG		CP
CRHBP	Parkinson's	NSCs	-3.094	4.86E-05	HTM	CbCx	IZ	CP
DIAPH2	Parkinson's	NSCs	-2.382	2.86E-05	AMG, DG, GP, LV	CbCx	SVZ	CP
EDNRB	Parkinson's	NSCs	2.31	6.13E-06	AMG, HTM, MY, TH	CbCx, Cx, HPC	SVZ	CP
EFEMP1	Parkinson's	NSCs	-2.669	2.84E-06	CGS, ET, GP	CbCx, HPC	SVZ	CP, IZ

EMP1	Parkinson's	NSCs	-3.5	1.58E-06	CbN, ET	CbCx, DG	IZ	CP
EPHA3	Parkinson's	NSCs	3.588	2.44E-06	Cx, TH	AMG, Cb, CC, HPC, LV	CP, IZ	
FABP3	Parkinson's	NSCs	-2.063	4.70E-06	HPC	Cb, CC, CGS, ET, LV	CP	SVZ
FOXA2	Parkinson's	NSCs	-3.314	1.05E-06	CbN, CC, GP, Pons, VT	CbCx, CGS	CP	SVZ
FRZB	Parkinson's	NSCs	3.605	3.62E-06	CbN, GP, LV, MY, Pons	CbCx, DG	CP	IZ
FZD10	Parkinson's	NSCs	2.84	1.15E-06	VT	Cx, DG, HTM	CP, SVZ	
GABBR1	Parkinson's	NSCs	-4.145	1.05E-06	AMG, DT	CbN, CC, Pons, LV, MY		SVZ
GABRA5	Parkinson's	NSCs	-3.424	1.44E-05	Cl, Cx, HPC, DT	Cb, MY	SVZ	CP, IZ
GAS1	Parkinson's	NSCs	2.413	3.21E-06	CC	HPC, HTM, Pons	SVZ	
GLIPR1	Parkinson's	NSCs	-2.483	4.95E-06			SVZ	CP
GPR183	Parkinson's	NSCs	-2.579	4.91E-05	CbN, CC, CGS, ET, GP	CbCx, DG		
HAS2	Parkinson's	NSCs	-2.258	2.86E-05	ET		CP, SVZ	IZ
HNMT	Parkinson's	NSCs	-2.466	1.33E-05	CGS, ET, GP, MTg	CbCx, DT	CP, SVZ	SVZ
IGFBP3	Parkinson's	NSCs	-4.274	3.48E-07	HPC, HTM	CbCx, GP		SVZ
IGFBP5	Parkinson's	NSCs	-2.406	2.91E-05	AMG, CC, CGS, VT	DT, HTM, Pons	IZ	CP, SVZ
KAL1	Parkinson's	NSCs	4.769	3.48E-07	AMG, HTM, Pons	Cb		CP
KCND3	Parkinson's	NSCs	-2.207	1.43E-05	CbCx, DG, MTg	CC, CGS, ET, HTM, LV, Pons	CP, IZ	
LAMB1	Parkinson's	NSCs	-2.027	7.90E-06	AMG, CbCx, DT, LV	CbN, CC, GP, MTg, Pons	IZ	SVZ
LAMP3	Parkinson's	NSCs	-2.004	5.23E-06				SVZ
LGR5	Parkinson's	NSCs	2.776	4.86E-05	AMG, CbN, CC, MTg, Pons, VT	CbCx, Cx, HPC, LV	IZ	SVZ
MATN2	Parkinson's	NSCs	-2.186	9.65E-06	AMG, CGS, GP, MTg, VT	CbCx, Cx, HPC	CP, SVZ	
ME1	Parkinson's	NSCs	-2.141	4.70E-06	Pons	CbCx	CP, IZ	
MEOX1	Parkinson's	NSCs	-2.564	8.03E-06			IZ	SVZ
NID2	Parkinson's	NSCs	-2.698	1.05E-06	CC, CGS, LV, VT	CbCx, DG, HTM	IZ	CP
NKX2-2	Parkinson's	NSCs	-3.738	8.74E-07	CbN, CC, GP, MY, Pons, VT	CbCx, Cx, HTM, LV	IZ	CP, SVZ
NMU	Parkinson's	NSCs	2.074	6.46E-06	Cx, DT	AMG, CbCx, CC, HPC	IZ	CP
NPPB	Parkinson's	NSCs	-2.054	2.35E-05	AMG, GP	Cx	SVZ	CP, IZ
NR2E1	Parkinson's	NSCs	2.023	9.37E-06	AMG	Cb, Pons, MY	IZ	CP, SVZ
NRP1	Parkinson's	NSCs	-2.434	4.79E-05	HPC	CbCx	IZ	CP
OLFML3	Parkinson's	NSCs	-2.983	9.55E-06	CbN, CC, GP	CbCx, DG, HTM	IZ	CP
PAPSS2	Parkinson's	NSCs	-2.38	3.60E-06	CbN, Cx, GP	CbCx, HPC, HTM	IZ	CP
PAX6	Parkinson's	NSCs	4.041	8.94E-07	CbCx	DT	SVZ	CP, IZ
PHLDA2	Parkinson's	NSCs	-3.111	1.89E-06		CbCx, GP, HPC	SVZ	IZ
PLAU	Parkinson's	NSCs	-2.14	2.69E-06	MY	CbCx		SVZ
PLOD2	Parkinson's	NSCs	-2.234	5.48E-05	ET, GP	CbCx, DT, HPC	SVZ	CP
PLP1	Parkinson's	NSCs	2.245	1.65E-05	CbN, CC, ET, GP, MY, Pons	CbCx, Cx, DG, DT		CP
PRDM13	Parkinson's	NSCs	3	2.96E-06	CGS, HTM		IZ, SVZ	CP
PTGER4	Parkinson's	NSCs	-2.252	2.55E-05	AMG, MY	CbCx, DT	SVZ	CP
RGS1	Parkinson's	NSCs	-3.293	2.09E-06	CbN, CC, CGS, ET, GP	CbCx, Cx, DG	IZ, SVZ	CP
SEPP1	Parkinson's	NSCs	-3.071	2.69E-06	CbN, CC, GP, MTg, Pons, VT	CbCx, HPC, HTM	SVZ	CP
SERPINF1	Parkinson's	NSCs	-3.03	5.58E-06	Cx, DT, LV	CbCx, GP, Pons	CP	IZ
SLIT3	Parkinson's	NSCs	-2.123	1.09E-05	AMG, CbCx, HPC	CC, HTM, MTg		CP, SVZ
SPP1	Parkinson's	NSCs	-3.577	2.69E-06	CbN, CC, GP, Pons, MY, TH	CbCx, Cx	IZ, SVZ	CP
STC2	Parkinson's	NSCs	-2.903	5.32E-06	HPC, DT	HTM	IZ	
TFAP2B	Parkinson's	NSCs	3.067	1.70E-06	CbCx		IZ	CP
TFF3	Parkinson's	NSCs	-2.351	5.35E-06	DT		IZ	CP

TFPI	Parkinson's	NSCs	-2.203	5.06E-05	CbN, MY	CbCx, DG	SVZ	CP
TFPI2	Parkinson's	NSCs	-2.113	1.82E-05	DT, MY	CbCx, Cx	IZ, SVZ	CP
TMEM100	Parkinson's	NSCs	-2.249	2.69E-06	AMG, HTM	CbCx		CP
UBD	Parkinson's	NSCs	-4.145	1.05E-06	LV		CP, SVZ	
ADAMTS12	Schizophrenia	Neurons	-4.15	5.67E-19	DT, MY	DG	SVZ	CP
ARRDC4	Schizophrenia	Neurons	3.90	3.41E-18	CC, ET, HPC, MY	CbCx, Cx	IZ	CP
ATP1A2	Schizophrenia	Neurons	-2.87	1.01E-08	GP, MTg, MY, Pons	CbCx, DG, LV	SVZ	CP
BCAM	Schizophrenia	Neurons	-2.21	1.62E-10	CbN, MTg, MY, Pons	CbCx, FL	IZ	SVZ
COL1A1	Schizophrenia	Neurons	-2.44	4.75E-04	CGS, DT, LV, Str		SVZ	CP
COL3A1	Schizophrenia	Neurons	-4.75	1.94E-16	LV, MY	CbCx, Cx, DG, DT	IZ	CP
COL6A3	Schizophrenia	Neurons	-2.11	2.51E-06	HPC, LV		IZ	SVZ
CYP26B1	Schizophrenia	Neurons	2.44	5.11E-04	AMG, CbCx	DG, HTM, MTg, MY, TH		SVZ
DCT	Schizophrenia	Neurons	-4.49	8.04E-13	Cx, Str	Cb, CC	SVZ	
EBF1	Schizophrenia	Neurons	3.52	6.09E-14	CbCx, MY	Cx, DG		CP
EFEMP2	Schizophrenia	Neurons	2.19	5.15E-05	GP, LV	HPC, MTg, Pons	SVZ	CP
ELN	Schizophrenia	Neurons	-2.59	3.83E-12	Cb, CGS, ET, LV	HPC, PL, TH		CP
FBLN2	Schizophrenia	Neurons	-3.23	1.82E-14	AMG, CbN, LV	Cb		CP
FBN1	Schizophrenia	Neurons	-2.84	1.38E-23	MY	Cb, HPC	SVZ	CP
FBN2	Schizophrenia	Neurons	-3.87	1.82E-16	DT, MTg		SVZ	CP
FEZF1	Schizophrenia	Neurons	-2.40	9.71E-11	AMG, HTM, MTg		SVZ	
IL17RB	Schizophrenia	Neurons	2.05	1.23E-04	CbCx, GP	Cx, HPC, MY, Pons		CP
KCNAB3	Schizophrenia	Neurons	2.24	1.14E-04	Cb, OL	HB, HPC, HTM	IZ, SVZ	CP
KDM5D	Schizophrenia	Neurons	5.74	1.53E-07	CbCx, CC, LV	AMG, DG, HTM, Pons		
LAMA2	Schizophrenia	Neurons	-4.25	2.32E-18	CGS, GP, MTg	AMG, HPC		SVZ
LAMB2	Schizophrenia	Neurons	-2.29	3.13E-09	GP, MY, LV	Cx, HPC, Pons	SVZ	CP
LAMC1	Schizophrenia	Neurons	-2.18	1.92E-09	Cb	CC, DT	IZ, SVZ	CP
LHX1	Schizophrenia	Neurons	-4.44	2.77E-16	CbCx	Cx, CC, HPC, Str		
LRRN1	Schizophrenia	Neurons	-2.11	8.41E-14	MY, Pons, Str	Cb, Cx, LV		CP
MATN2	Schizophrenia	Neurons	-3.21	1.19E-17	AMG, CC, CGS, GP, MTg	CbCx, Cx, HPC,		CP
NEDD4L	Schizophrenia	Neurons	2.21	1.24E-10	HPC, Str	CC, CGS, MTg	SVZ	
NID2	Schizophrenia	Neurons	-2.82	2.72E-10	CC, CGS, GP, VT	CbCx, DG, MTg	IZ	CP
OTX2	Schizophrenia	Neurons	-2.42	1.89E-16	Cb, DT, LV, MTg		SVZ	
PRSS35	Schizophrenia	Neurons	-4.63	3.74E-23	AMG, BG, Cl, FL	Cb, LV, MTg, MY, Pons	SVZ	CP, IZ
RNASEL	Schizophrenia	Neurons	2.18	2.19E-04	Pons, MY	CbCx, DG		CP
RNF128	Schizophrenia	Neurons	3.17	1.27E-05	HPC, HTM, MTg, Pons	CbCx, CC, DT, LV		SVZ
RPL30	Schizophrenia	Neurons	2.05	1.14E-03	CbCx, CC, GP	Cx, DG, DT	IZ	
RPL35A	Schizophrenia	Neurons	2.14	7.92E-04	CbCx, CC, GP	DT, HPC, MTg, Pons	IZ	
RPS11	Schizophrenia	Neurons	2.06	8.62E-04	CbCx, CC, GP	DT, HPC, MTg, Pons	IZ	
RPS19	Schizophrenia	Neurons	2.66	3.60E-04	CbCx, CC, GP	DT, HPC, MTg, Pons	IZ	
RPS4Y1	Schizophrenia	Neurons	7.28	2.07E-07	CbCx, CC, GP	AMG, DT, HPC, MTg, Pons	IZ	
SCGN	Schizophrenia	Neurons	5.08	2.49E-07	CbCx, HPC, HTM	CbN, CC, Pons, Str, TH	SVZ	
SERPING1	Schizophrenia	Neurons	-2.27	9.21E-08	ET, LV, MY	CbCx, CC, HPC, MTg	SVZ	
SERPINH1	Schizophrenia	Neurons	-2.09	4.13E-10	CbN, LV, MY, Pons	HPC	SVZ	CP
SLA	Schizophrenia	Neurons	4.46	7.77E-10	AMG, CbN, DG, MY, Pons	CbCx, HTM, MTg, PL	CP, IZ	SVZ
SOCS2	Schizophrenia	Neurons	2.13	1.56E-04	Cl, HPC, LV	AMG, Cb, CC, HTM, TH	CP	
SULF1	Schizophrenia	Neurons	-4.62	1.52E-26	CGS, DT, HTM, LV, Pons	CbCx, HPC		
SULF2	Schizophrenia	Neurons	-3.14	1.22E-27	HPC, MY, Pons	CbCx, DT, OL	CP	SVZ
TCEAL2	Schizophrenia	Neurons	2.52	4.94E-05	CGS, MY, Pons	BG, CbCx, CC	CP	SVZ

TRIM58	Schizophrenia	Neurons	2.48	9.75E-04	HTM, MTg, MY, Pons	CbCx, CC, HPC, LV		SVZ
MGP	SMA	Neurons	-2.63	1.53E-02	CGS, LV, TL	Cb, HPC		CP

**Adult Brain Expression Abbreviations**		FL	Frontal Lobe	**Adult Brain Legend**	**Prenatal Brain Legend**	
AMG	Amygdala	GP	Globus Pallidus			
BG	Basal Ganglia	HPC	Hippocampus	Telencephalon (Cortex)		
Cb	Cerebellum	HTM	Hypothalamus			
CbCx	Cerebellar Cortex	LV	Lateral Ventricles	Telencephalon (Nuclei)	CP	Cortical Plate
CbN	Cerebellar Nuclei	MTg	Midbrain Tegmentum			
CC	Corpus Callosum	MY	Myelencephalon	Diencephalon		
CGS	Central Glia Substance	OL	Occipital Lobe			
Cx	Cerebral Cortex	PL	Parietal Lobe	Metencephalon	IZ	Intermediate Zone
CxN	Cerebral Nuclei	Pons	Pons	Mesencephalon		
Cl	Claustrum	Str	Striatum			
DG	Dentate Gyrus	TH	Thalamus	Myelencephalon		
DT	Dorsal Thalamus	TL	Temporal Lobe			
ET	Epithalamus	VT	Ventral Thalamus	Other Structures	SVZ	Subventricular Zone

Appendix Table S12. **Proposed Minimal Information Checklist for iPSC Research Studies**

Suggested Minimal Information Categories	Checklist			Minimal Information Subcategories	Description
	✓	✖	N/A	Study title	
	✓	✖	N/A	IRB approval date	
	✓	✖	N/A	IRB affiliation	
	✓	✖	N/A	Reference publication (PMID)	
	✓	✖	N/A	GEO ID	
	✓	✖	N/A	Platform ID	
Study	✓	✖	N/A	Platform name	
	✓	✖	N/A	Age	
	✓	✖	N/A	Gender	
	✓	✖	N/A	Diagnosis	
	✓	✖	N/A	Source cell type (i.e fibroblast, lymphocyte, etc.)	
Clinical information of patients and source cell isolation	✓	✖	N/A	Standardized cell line name (Luong *et al*. 2011, Cell Stem Cell)	
	✓	✖	N/A	Reprogramming methodology	
	✓	✖	N/A	Purity of cell population	
	✓	✖	N/A	QC methodology for pluripotency	
Generation of iPSCs protocol	✓	✖	N/A	Karyotyping	
	✓	✖	N/A	Vector type	
Gene delivery methods for cells	✓	✖	N/A	Genes delivered	
	✓	✖	N/A	Cell type(s)	
	✓	✖	N/A	Differentiation protocol(s)	
	✓	✖	N/A	Purity of cell population(s)	
Differentiation of iPSCs to any cell type	✓	✖	N/A	QC method(s) for validation of differentiated cell type(s) (i.e. Gene marker(s), surface antigen(s), etc.)	
	✓	✖	N/A	Basal media used (specify by cell type(s))	
	✓	✖	N/A	Passage methodology	
	✓	✖	N/A	No. of passage(s)	
	✓	✖	N/A	Culture additive(s) and concentration(s)	
	✓	✖	N/A	% O2	
	✓	✖	N/A	% CO2	
Cell culture/maintenance	✓	✖	N/A	Culture temperature	

Validation of the mutation being studied	✓	✖	N/A	Validation methodology	
	✓	✖	N/A	Cell type(s) validated	
Procedures of assays/<br>specific phenotypic search methods	✓	✖	N/A	Protocol(s) of phenotypic assay(s)	
	✓	✖	N/A	Gene expression profiling method(s)	
	✓	✖	N/A	Treatment(s) and concentration(s) used	
