# Supplementary material for: iPhemap: an atlas of phenotype to genotype relationships of human iPSC models of neurological diseases
Source: EMBO Mol Med. 2017 Oct 19;9(12):1742–62. doi: 10.15252/emmm.201708191 (PMC5731211; doi:10.15252/emmm.201708191)
Supplement: Supplementary file 2 — Expanded View Figures PDF [file EMMM-9-1742-s002.pdf]

Expanded View Figures

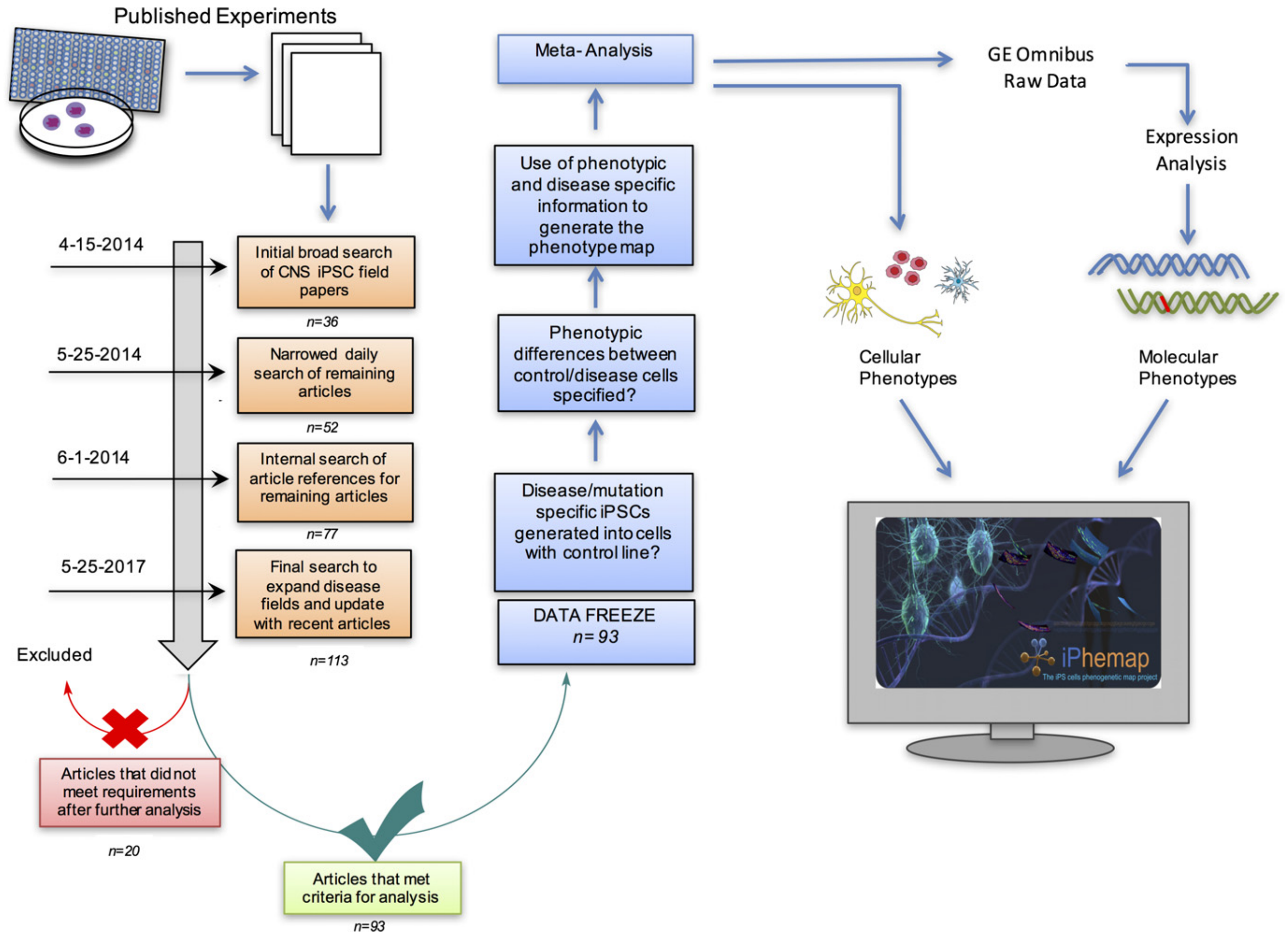

Figure EV1. Diagram of meta-analysis flowchart showing our search for potential CNS iPSC papers spanned over three years.

Pipeline includes our inclusion and exclusion criteria for selection of candidate papers; 93 of which met these requirements and were examined in our study.

 © 2017 The Authors

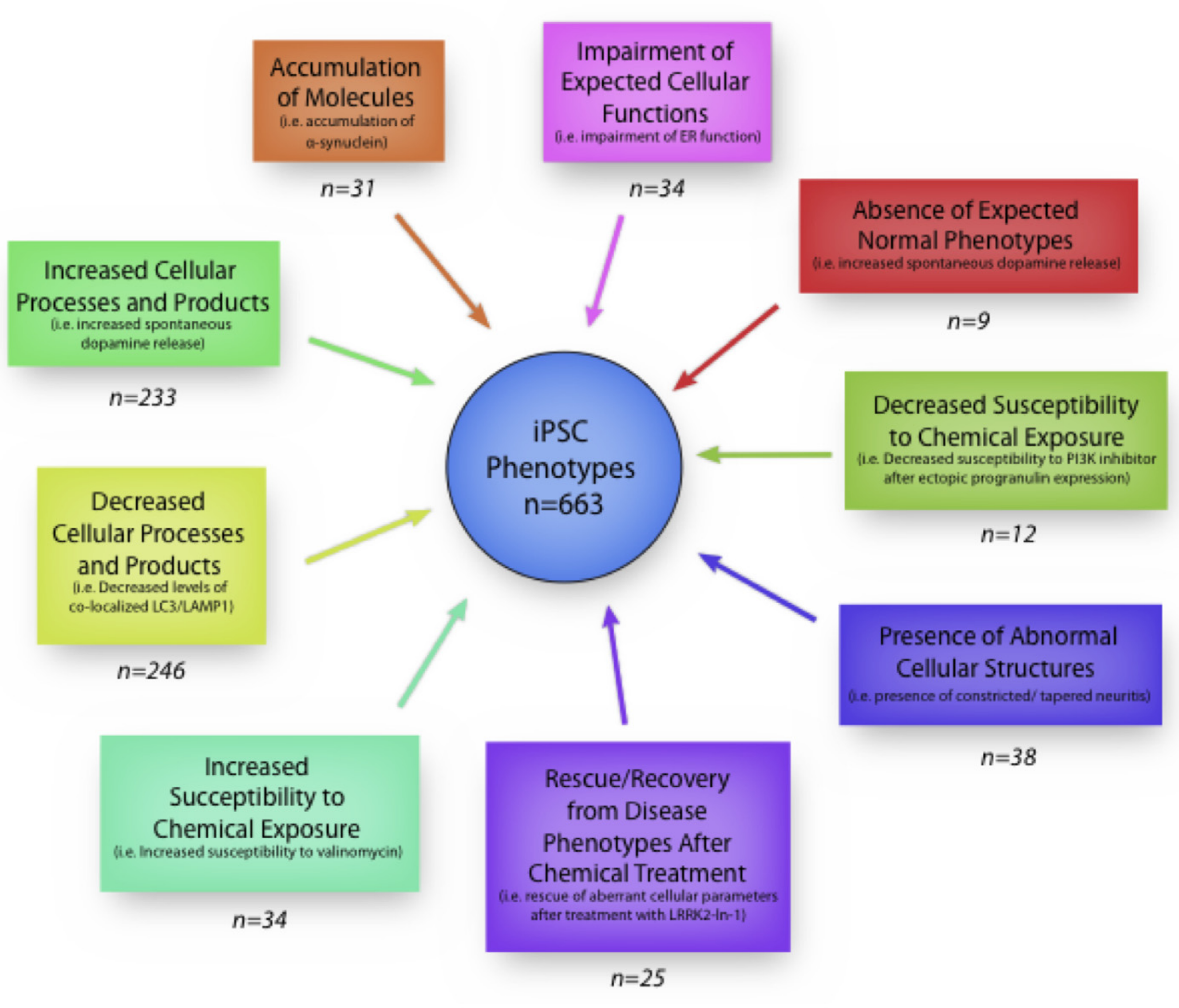

Figure EV2. Taxonomy of phenotype classes established to classify our 663 phenotypes.

Phenotype classes include definitions and examples from our analysis. Colors of each phenotype class are preserved through all of the Figures.

© 2017 The Authors

A

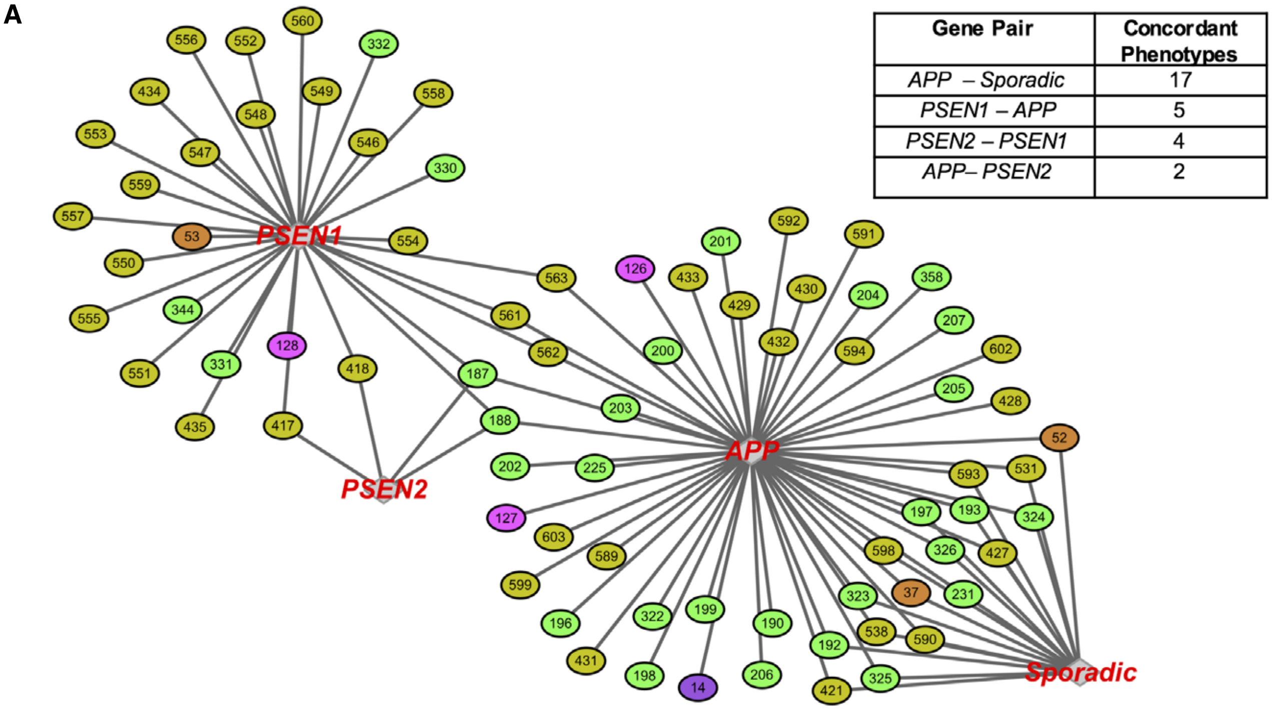

Gene Pair	Concordant Phenotypes
APP – Sporadic	17
PSEN1 – APP	5
PSEN2 – PSEN1	4
APP– PSEN2	2

B

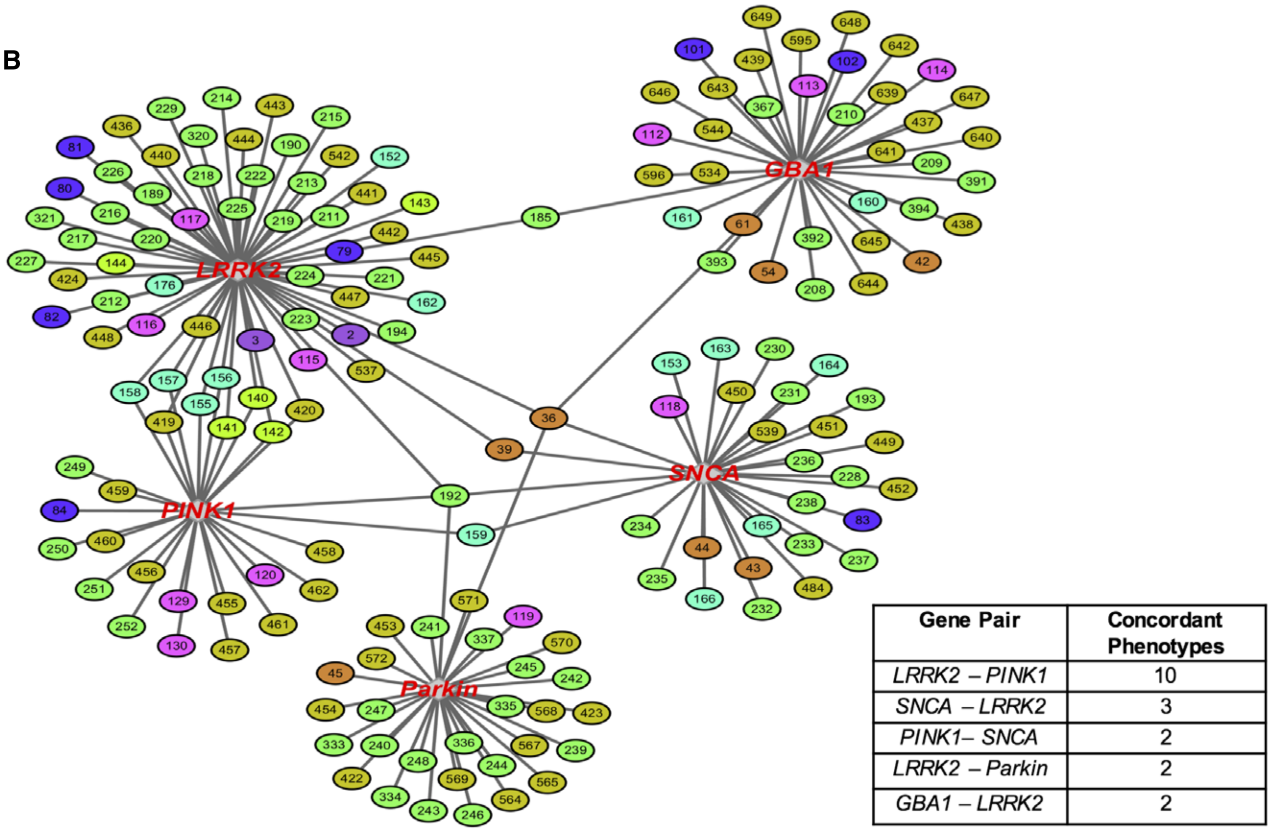

Gene Pair	Concordant Phenotypes
LRRK2 – PINK1	10
SNCA – LRRK2	3
PINK1– SNCA	2
LRRK2 – Parkin	2
GBA1 – LRRK2	2

Figure EV3. Phenogenetic networks of genes linked to Alzheimer's and Parkinson's disease reveal concordant phenotypes.

A, B A nuanced phenogenetic network view of genes associated with (A) Alzheimer's disease and (B) Parkinson's disease. The number of concordant phenotypes shared by gene pairs of AD and PD is outlined in tables, with *APP-Sporadic* and *APP-PSEN1* having the most in AD and *LRRK2-PINK1* in PD.

 © 2017 The Authors

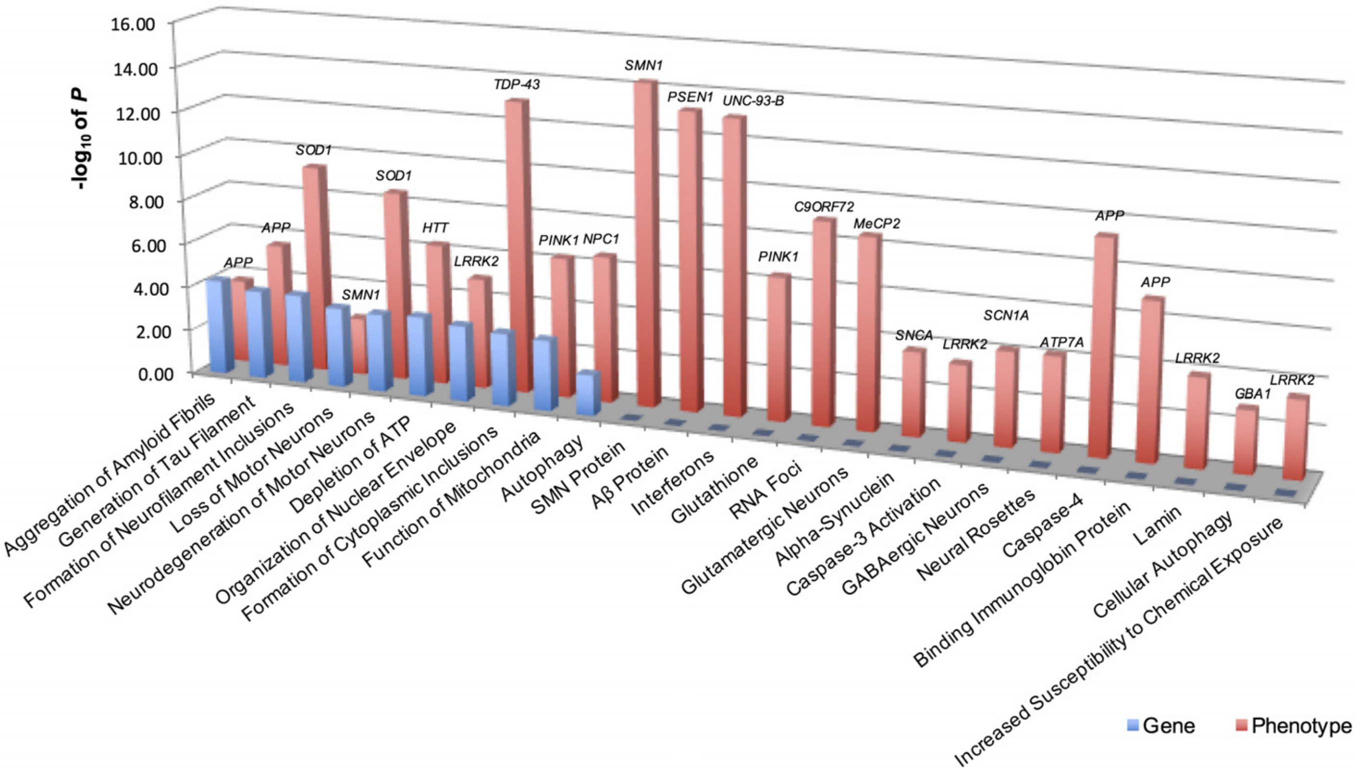

Figure EV4. Comparison between phenotype and gene ontology.

Pairwise statistical comparison of functional annotations derived from well-established gene ontologies and phenotype ontology, which notably includes several novel phenotype ontology terms, $n = 15$, that have yet to be reported in gene ontologies.

© 2017 The Authors
