# Supplementary material for: iPhemap: an atlas of phenotype to genotype relationships of human iPSC models of neurological diseases
Source: EMBO Mol Med. 2017 Oct 19;9(12):1742–62. doi: 10.15252/emmm.201708191 (PMC5731211; doi:10.15252/emmm.201708191)
Supplement: Supplementary file 3 — Web Resources EV1 [file EMMM-9-1742-s003.pdf]

Web Resources

iPhemap: An atlas of phenotype to genotype relationships of human iPSC models of neurological diseases

Ethan W. Hollingsworth[1,2], Jacob E. Vaughn[1,2], Josh C. Orack[1,2], Chelsea Skinner[1,2], Jamil Khouri[1,2], Sofia B. Lizarraga[3], Mark E. Hester[4], Fumihiro Watanabe[1], Kenneth S. Kosik[5] and Jaime Imitola[1,2,6]

Table of Contents

1. **iPhemap webtool development**

2. **Web resources user guide**

3. **Landing page legend**

iPhemap web tool development

Following the review and analysis of all observed phenotypes, we sought for a way to share this information in an easily accessible and searchable manner. In order to achieve this task, we developed an online web tool. We designed this website through the web framework, Django, using the programming language Python. In addition, we wrote several scripts to transform and sanitize the raw data into SQL tables. To make the data publicly available and searchable, we first transformed the data derived from the meta-analysis, which was organized into excel spreadsheets, into an SQL database. This was achieved by initially exporting it to the more common .html format, which we then extracted into the desired data format.

We also constructed data models to reflect information about individual genes, associated phenotypes and studies, which all corresponded to a separate SQL table with data about the respective object. We formatted each SNP to carry the following data fields: the name of the gene, the name of the associated gene, the related disease, a reference to the PMID of the paper, and a list of the corresponding phenotypes. Additionally, we attached another data field, a description of the phenotype itself and the cellular type that it was observed in, to each respective phenotype. We also documented the first author listed, the PMID, the biological model that the study was employed, the year of publication, the type of experiment conducted, the starting cell type and the target cell type used for each article from our meta-analysis. Moreover, for the studies that performed microarray analysis, we recorded the corresponding GEOID of each article, a link to its external page with its related description and the name of its platform along with its GEO used.

We generated several templates to display this data effectively on the website. To maximize user friendliness for the website, we designed an "about" page to provide a description, methods and usage information. Additionally, we created a search page to provide an interface to

search and filter desired data. We also matched queries given to the search with all available information, unless specified by the filters to be constrained to a specific disease or topic. We then split the results of each search into two views: the first provided a list of cellular phenotypes for each gene that matched the query while the second view contained figures of molecular phenotypes associated with each gene. For queries regarding studies that conducted microarray analysis, we integrated the treemap and pathway figures into the results. Furthermore, we gave each investigated gene a hyperlink to its specific study. To provide a list of all of the studies referenced, we also formulated a third page, titled "studies" and we similarly gave each study a hyperlink to the resources listed above. Finally, we devised a password protected content management system to afford the website with the ability to add new data to the system dynamically through an administration interface. Consequently, we enable the website with the ability for data to be added or changed in the format specified above by an administration user, which we established to be able to use this content management system.

Upon completion, we deployed the website onto a PaaS (Platform as a Service) provider (Openshift) in order to make it publicly accessible. In order to apply changes to the site from a remote location, we wrote several scripts. During each of our deployments to a newer version, the scripts were automatically run to check and satisfy any dependencies specified for the Python programming language, duplicate and copy any changes to the data tables above, and restart the server instance to reflect these changes in the live site.

Our website, iPhemap, allows users to search through a catalogue of phenotypes and their related diseases for any information reported by this paper. Our database is searchable by disease name, disease gene, gene mutation, phenotype, and the PubMed ID of the examined articles.

However, since this area of research is still growing, we created an administration user in order to provide the catalogue with the ability to add newly published information to this database.

URLs. A supporting online website summarizing phenogenetic associations from this study and providing a searchable version of the phenogenetic network is accessible at http://www.iPhemap.org. A fully annotated version of the network in Cytoscape format is available for download at http://

Gene expression data analysis is available at http://www.iPhemap.org

Web resources user guide

To facilitate searching through our data we developed a web-based resource for data mining into a publicly available, continuously updated database that catalogs all the *in vitro* neuronal disease-phenotypes iPhemap.org. Our website creates an easy to use interface for searching CNS cellular phenotypes of neurological disease.

1. *Home Search Interface*- Two drop-down menus containing disease names and specific cell types studied are placed below a main search bar on the main search page. Disease names, genes, mutations, and phenotypes can be typed into the search bar with additional criteria added into the drop-down menus to search all the information contained in the database.

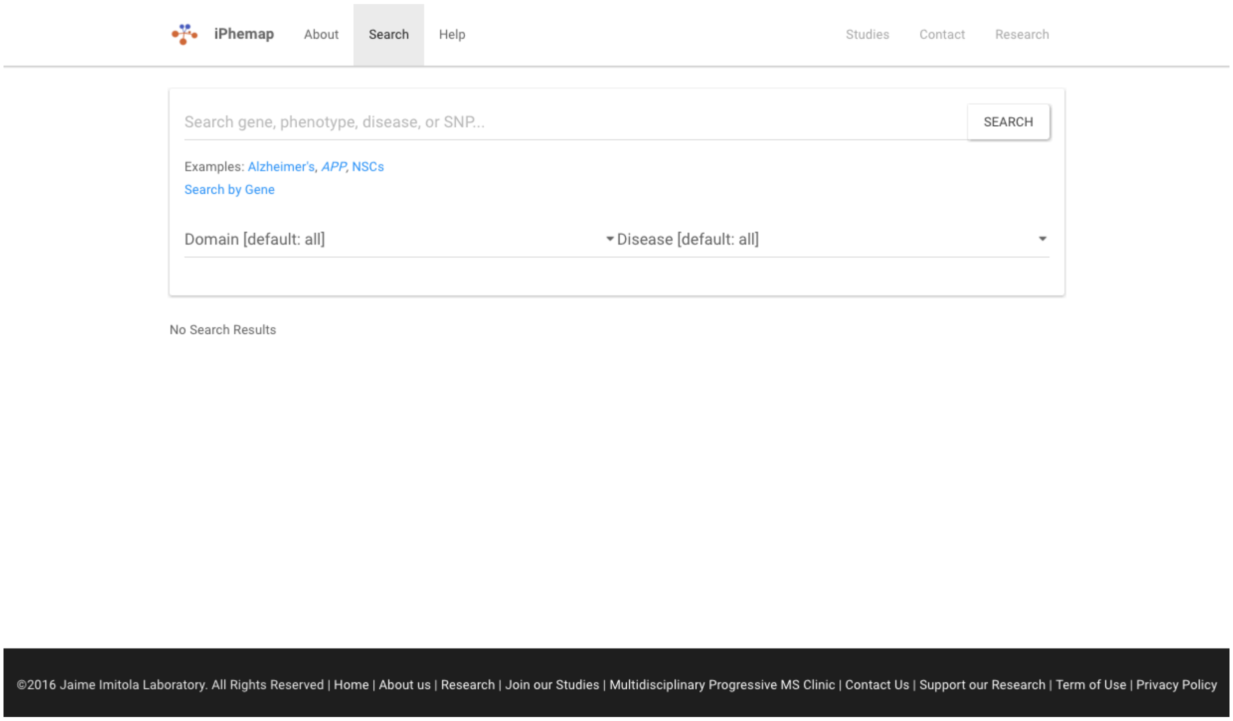

2. *Phenotypic Information*- After the user enters their desired search criteria and presses search, a page containing information matching their search is rendered. Disease name, gene, mutation, PMID number, and phenotypes are generated based on catalogued information with the searched words highlighted in yellow. (The gene *APP* is used as an example search below).

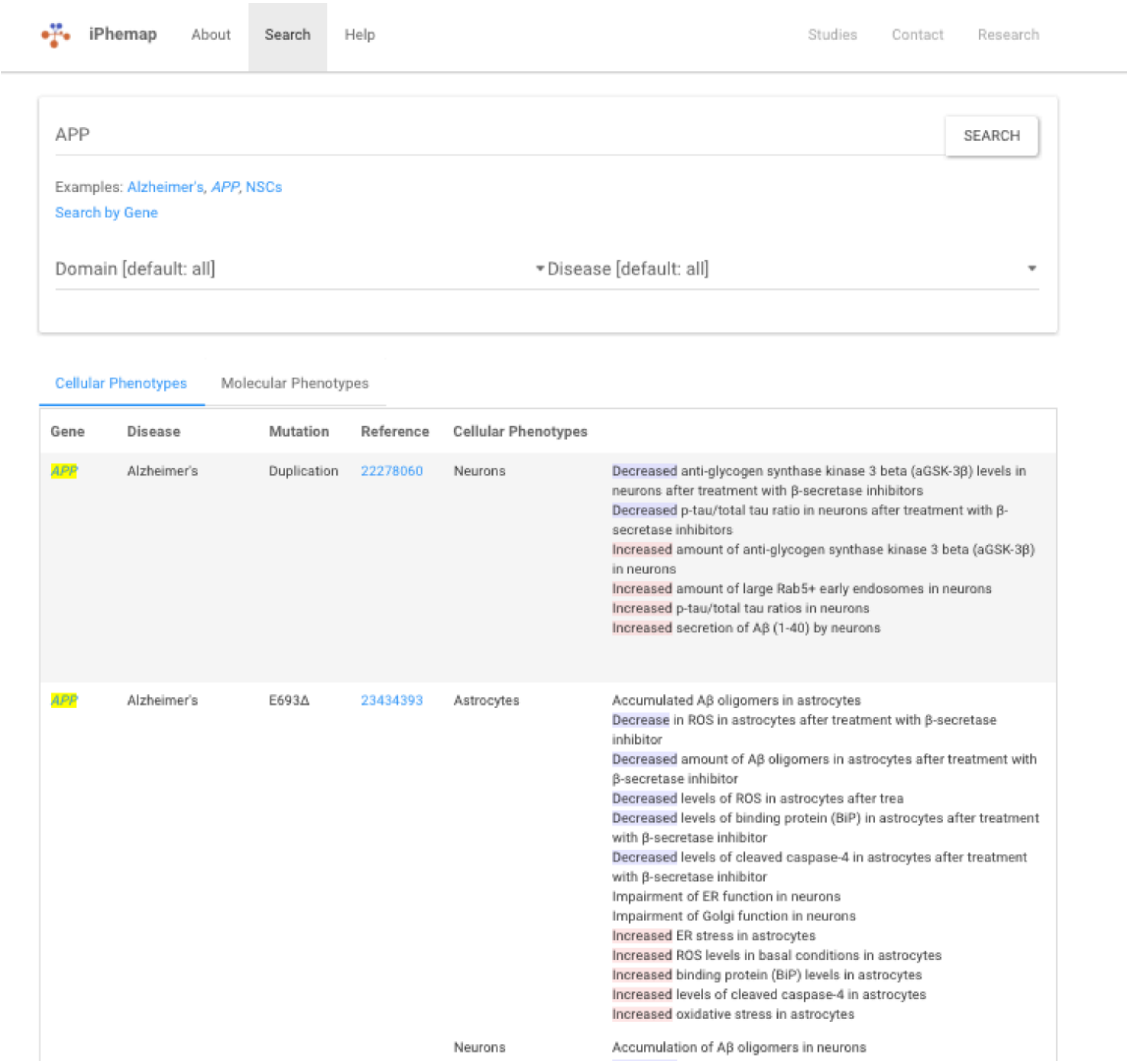

3. *Narrowing Search*- Users can narrow their results further after searching by using drop-down menus. Specification of cell type and/or disease name by drop-down menus can yield fewer

but more desirable results. (a) Specifying astrocytes for cell type to the above search is used as an example below to yield (b) the narrowed search results.

(a).

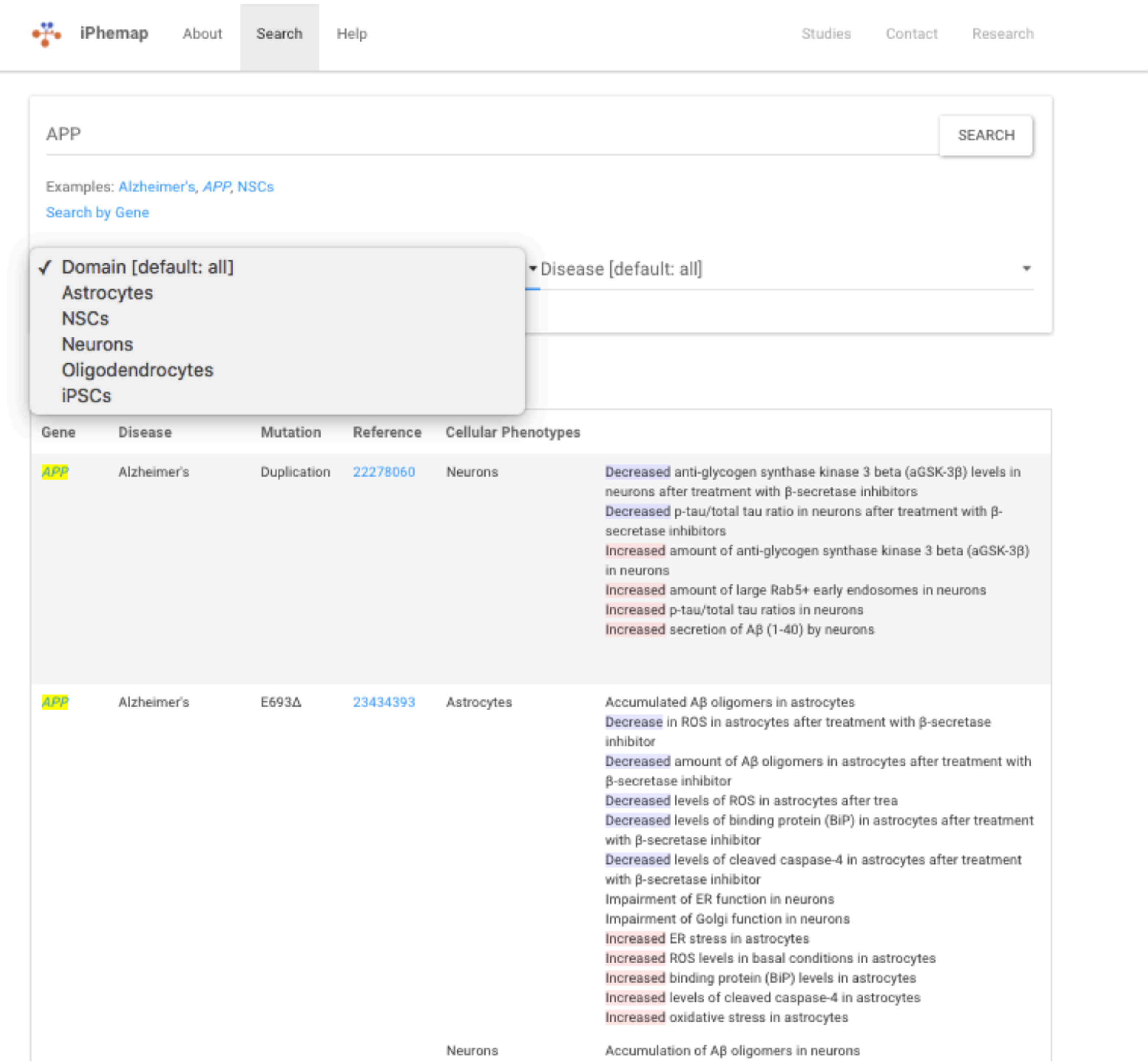

(b).

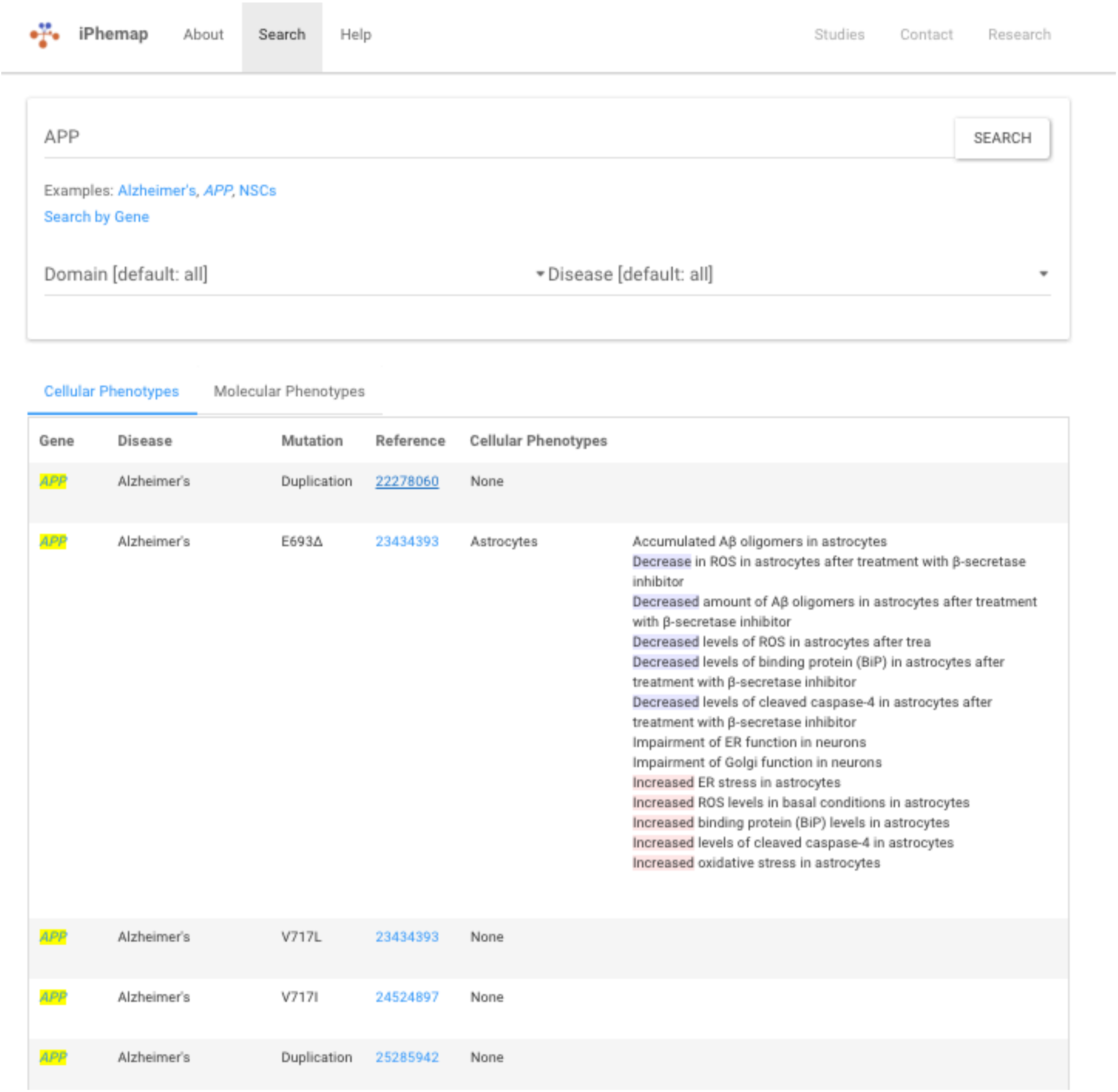
iPhemap
About
Search
Help
Studies
Contact
Research
APP
SEARCH
Examples: Alzheimer's, APP, NSCs
Search by Gene
Domain [default: all]
Disease [default: all]
Cellular Phenotypes
Molecular Phenotypes
Gene | Disease | Mutation | Reference | Cellular Phenotypes
APP | Alzheimer's | Duplication | 22278060 | None
APP | Alzheimer's | E693Δ | 23434393 | Astrocytes
Accumulated Aβ oligomers in astrocytes
Decrease in ROS in astrocytes after treatment with β-secretase inhibitor
Decreased amount of Aβ oligomers in astrocytes after treatment with β-secretase inhibitor
Decreased levels of ROS in astrocytes after trea
Decreased levels of binding protein (BiP) in astrocytes after treatment with β-secretase inhibitor
Decreased levels of cleaved caspase-4 in astrocytes after treatment with β-secretase inhibitor
Impairment of ER function in neurons
Impairment of Golgi function in neurons
Increased ER stress in astrocytes
Increased ROS levels in basal conditions in astrocytes
Increased binding protein (BiP) levels in astrocytes
Increased levels of cleaved caspase-4 in astrocytes
Increased oxidative stress in astrocytes
APP | Alzheimer's | V717L | 23434393 | None
APP | Alzheimer's | V717I | 24524897 | None
APP | Alzheimer's | Duplication | 25285942 | None

4. *Molecular Phenotypes*- Additional information regarding molecular phenotypes of the user's search results may be accessed by selecting the "Molecular Phenotypes" tab. Any information present in the database is provided in the form of a treemap. Upregulation and downregulation of molecular network pathways and gene ontology are seperated for easier viewing. Maps are clickable for zoomed in viewing. (Search criteria of "Gene: *APP*" is entered and the molecular tab is selected and used as an example below.)

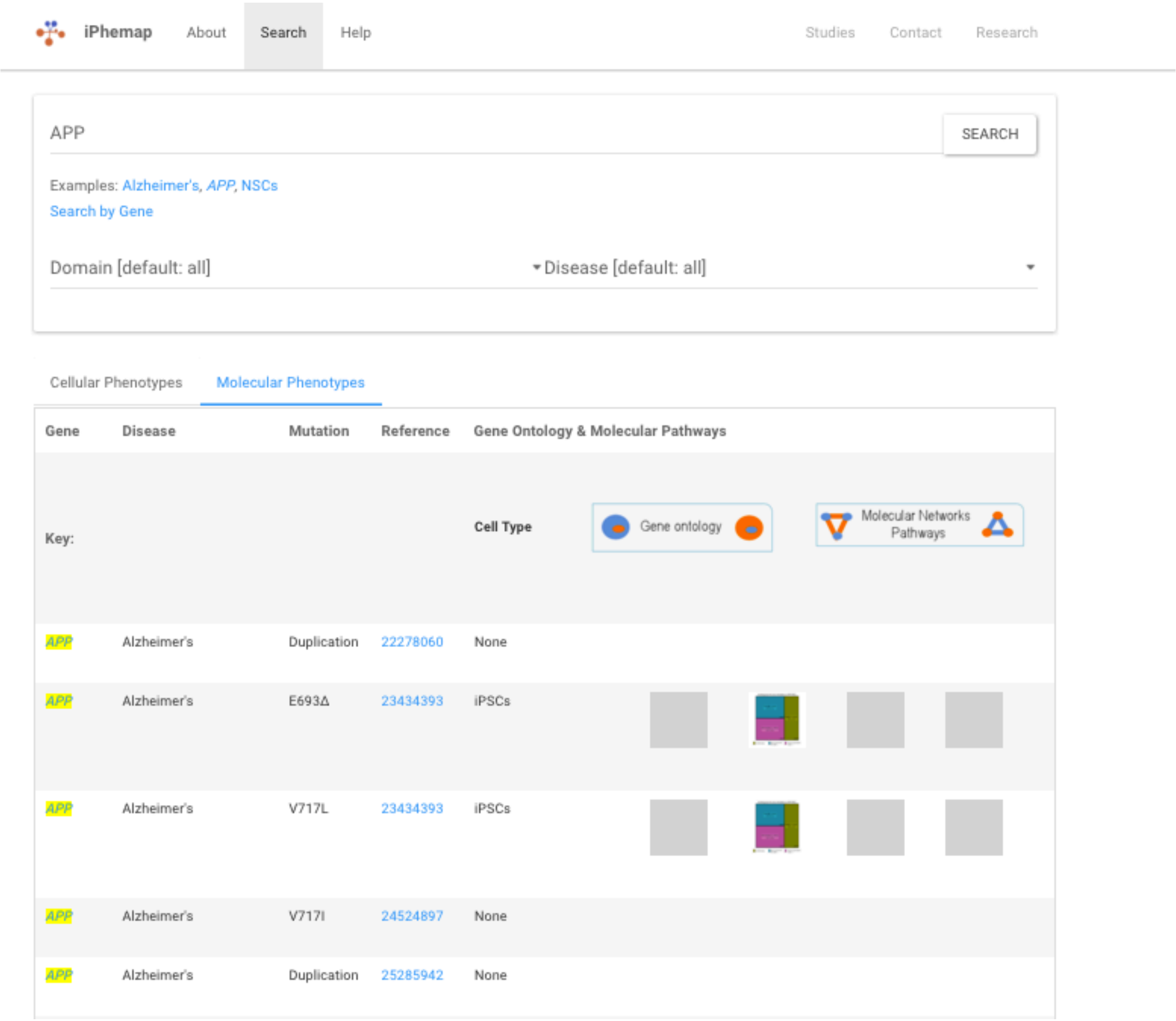

5. *Localization of Dysregulated Gene Expression*

Additional information regarding the localization of dysregulated gene expression of the user's search results may be accessed by selecting the "Dysregulated Gene Expression" tab. Any information present in the database is provided in the form of a heatmap. Heatmaps of the developmental transcriptome, prenatal and adult human brain are separated for easier viewing. Maps are clickable for zoomed in viewing. (Search criteria of "Gene: *FXN*" is entered and the corresponding tab is selected and used as an example below.)

6. *Analyzed Studies*- a list of all articles and studies analyzed in the database is accessible by clicking the studies tab in the top right corner of every page. A variety of information is present including the authors of each study, disease studied, year published, reprogramming method, starting and target cell type, number of disease and control patients, and GEO information for any including such information.

iPhemap About Search Help Studies Contact Research

Studies

Published Studies.

First Author	Disease	Gene/Mutation	Species	Year	Reprogramming	Starting Cell Type	Generated Celltype	Number of Disease Patients	Number of Control Patients	Utilization of Gene-Editing or Isogenetic Cell Lines	GEO ID	Platform Name	Platform GEO	PMID
Ebert	Spinal Muscular Atrophy	SMN1/ Not Specified	Homo sapiens	2009	Lentiviral Infection of Primers for OCT 4, SOX 2, NANOG, LIN 28, HoxB4, SMN, and GAPDH	Fibroblast	Neurons, Astrocytes	1	1	False	GSE13828	HG-U133_Plus_2	GPL570	19098894
Marchetto	Rett Syndrome	MeCP2/ Q244X	Homo sapiens	2010	Retrovirus vectors containing the Oct4, c-Myc, Klf4 and Sox2	Fibroblast	Neurons	4	5	False	GSE21037	HuGene-1_0-st	GPL6244	21074045
Zhang	Huntington's Disease	HTT/ CAG repeats	Homo sapiens	2010	VSVg retroviruses of Sox2, Klf4, Oct3/4, and/or c-Myc	Fibroblast	NSCs	1	1	False				21037797
Sherman	Friedreich's Ataxia	FXN/ Long GAA·TTC repeats	Homo sapiens	2010	VSV-G Retrovirus Vectors for Oct3/4, Sox2, Klf4, and c-Myc	Fibroblast	iPSCs	2	1	False	GSE22651	Illumina HumanHT-12 V3.0	GPL6947	21040903
Yagi	Alzheimer's Disease	PSEN1/ A246E, PSEN2/ N141I	Homo sapiens	2011	retroviral transduction of OCT4, SOX2, KLF4, LIN28 and NANOG	Fibroblast	Neurons	1, 1	1	False	GSE28450	Agilent-022060	GPL10123	21900357
Nguyen	Parkinson's Disease	LRRK2/ G2019S	Homo sapiens	2011	retroviruses of OCT4, SOX2, and KLF4	Fibroblast	Neurons	1	1	False				21362567
Byers	Parkinson's Disease	SNCA/ Triplication	Homo sapiens	2011	Retroviral gene insertion of OCT4, SOX2, KLF4, and c-MYC	Fibroblast	Neurons	1	1	False				22110584
Devine	Parkinson's Disease	SNCA/ Triplication	Homo sapiens	2011	pMXs-cMyc #13375, pMXs-Klf4 #13370, pMXs-Oct4 #13366, pMXs-Sox2 #13367 reprogramming factors from Addgene	Fibroblast	Neurons	1	1	False	GSE28366.	HumanOmni1-Quad	GPL8882	21863007
Seibler	Parkinson's Disease	PINK1/ Q456X, PINK1/ V170G	Homo sapiens	2011	Retroviral pMIG vectors OCT4, SOX2, cMYC and KLF4	Fibroblast	Neurons	3	1	False				21508222
Chang	Spinal Muscular Atrophy	SMN1/ not specified	Homo sapiens	2011	retroviral vectors containing Oct4, Sox2, c-Myc, Klf4	Fibroblast	Neurons	1	1	False				21956898
Kim	Rett Syndrome	MeCP2/ Q244X	Homo sapiens	2011	pMIG retrovirus expressing OCT4, SOX2, KLF4, and MYC	Fibroblast	Neurons, NSCs, iPSCs	5	5	False				21807996

6. *Study Specific Access*- In order to access a specific study from a results page generated by a search, the user may click the PMID number. The user will be guided to the studies page with their

desired study contained inside a black box. (The top study from an *APP* search is used as an example below. (a) Initial *APP* search, (b) studies page with highlighted study.

(a).

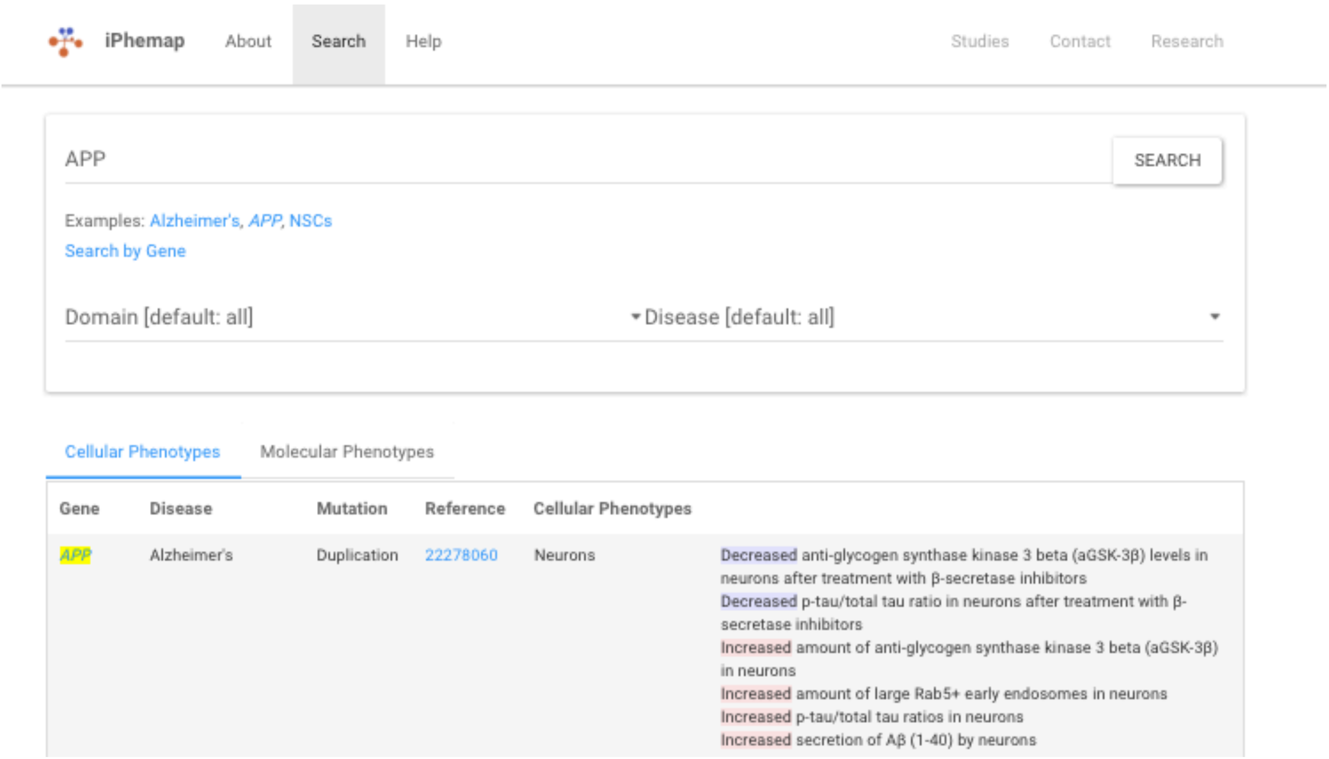

(b).

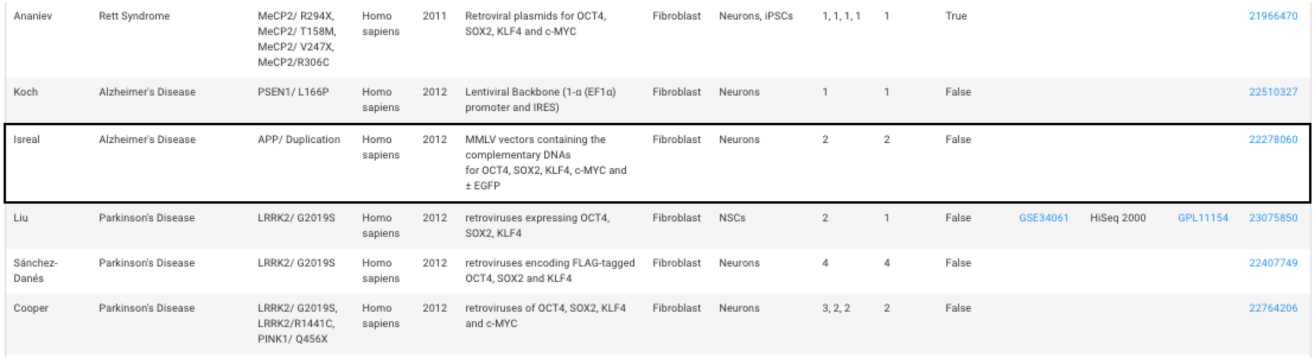

Ananiev	Rett Syndrome	MeCP2/ R294X, MeCP2/ T158M, MeCP2/ V247X, MeCP2/R306C	Homo sapiens	2011	Retroviral plasmids for OCT4, SOX2, KLF4 and c-MYC	Fibroblast	Neurons, iPSCs	1, 1, 1, 1	1	True				21966470
Koch	Alzheimer's Disease	PSEN1/ L166P	Homo sapiens	2012	Lentiviral Backbone (1-α (EF1α) promoter and IRES)	Fibroblast	Neurons	1	1	False				22510327
Isreal	Alzheimer's Disease	APP/ Duplication	Homo sapiens	2012	MMLV vectors containing the complementary DNAs for OCT4, SOX2, KLF4, c-MYC and ± EGFP	Fibroblast	Neurons	2	2	False				22278060
Liu	Parkinson's Disease	LRRK2/ G2019S	Homo sapiens	2012	retroviruses expressing OCT4, SOX2, KLF4	Fibroblast	NSCs	2	1	False	GSE34061	HiSeq 2000	GPL11154	23075850
Sánchez-Danés	Parkinson's Disease	LRRK2/ G2019S	Homo sapiens	2012	retroviruses encoding FLAG-tagged OCT4, SOX2 and KLF4	Fibroblast	Neurons	4	4	False				22407749
Cooper	Parkinson's Disease	LRRK2/ G2019S, LRRK2/R1441C, PINK1/ Q456X	Homo sapiens	2012	retroviruses of OCT4, SOX2, KLF4 and c-MYC	Fibroblast	Neurons	3, 2, 2	2	False				22764206

7. *About iPhemap*- in order to access general information regarding the iPhemap database, click the “about” tab at the top left corner of every webpage. A page with a brief description regarding the initial study and its results is generated, along with citation information, related links, announcements and updates.

Landing page legend

iPhemap landing page. Online, searchable database containing all 663 reported cellular phenotypes, molecular phenotypes and spatiotemporal localization of dysregulated gene expression, when available.

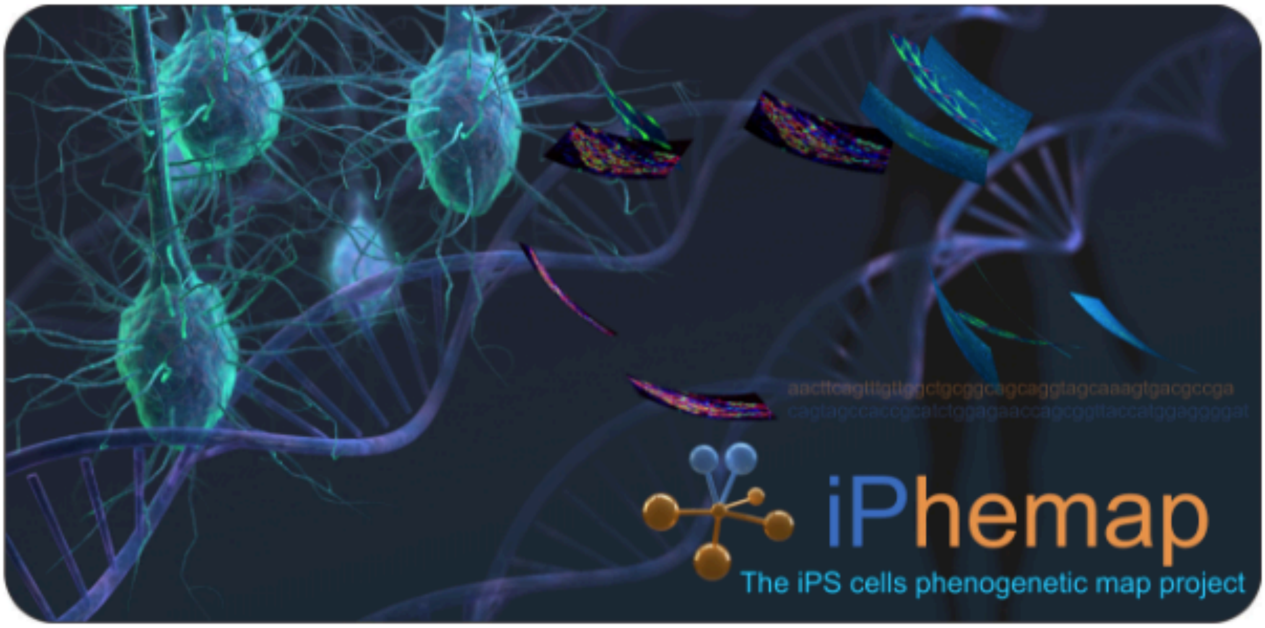

The **iPS cell phenogenetic map project "iPhemap"** is a comprehensive, continuously updated database that aims to provide a field synopsis and catalog all of the *in vitro* CNS cell-derived disease phenotypes from induced pluripotent stem cells (iPSCs) derived from patients with neurological diseases. You can search cellular and molecular phenotypes from 93 published reports. We characterized 663 distinct cellular phenotypes and the resulting relationships between genotypes and phenotypes into a phenogenetic map that can be used to build new hypotheses in the field of neurological disease modeling, and to identify potential new opportunities to design novel drug strategies. The project comprises a comprehensive catalog of phenogenetic profiles from patient derived-iPSCs from highly curated, published reports and returns: 1) Cellular phenotypes from iPSCs, neural stem cells, oligodendrocytes, astrocytes, and neurons with genetic mutations linked to neurological diseases. 2) Molecular phenotypes and dysregulated pathways, when available, from gene ontology analyses of gene expression profiles. 3) Spatial and temporal expression patterning of dysregulated genes during development and in the prenatal and adult human brain, when available, from the Allen Brain Atlas and visualized in heatmaps.

Citing iPhemap

Hollingsworth E, Vaughn JE, Orack JC, Skinner C, Khouri J, Lizarraga SB, Hester ME, Watanabe F, Kosik KS, Imitola J. iPhemap: Phenotype to genotype relationships of human iPSC models of neurological diseases (2017) *Submitted*

Announcements

May 10, 2017: Please read our recent publication in Cell Stem Cell. Big-Data-Driven Stem Cell Science and Tissue Engineering: Vision and Unique Opportunities. Del Sol A, Thiesen HJ, Imitola J*, Carazo Salas RE. Cell Stem Cell. 2017 Feb 2;20(2):157-160. doi: 10.1016/j.stem.2017.01.006

Links

- NIMH Repository and Genomics Resource
- The NIMH Stem Cell Center, hiPSC Cell Line Data
- Biorepositories
- Database of Genotypes and Phenotypes (dbGaP)
- Clinical Phenotyping Data through the NIMH Data Archive
- Genome Browser
- KEGG Genome
- ENCODE
- NIH Connectome
