# Supplementary material for: iPhemap: an atlas of phenotype to genotype relationships of human iPSC models of neurological diseases
Source: EMBO Mol Med. 2017 Oct 19;9(12):1742–62. doi: 10.15252/emmm.201708191 (PMC5731211; doi:10.15252/emmm.201708191)
Supplement: Supplementary file 4 — Movie EV1 [file EMMM-9-1742-s004.zip › EMM-2017-08191-Movie-EV1/Movie EV1_Legend.docx]

**Movie EV1.** Step by step, the movie explains the principles of phenotype to genotype relationships, or phenogenetics, and details the phenogenetic findings from our meta-analysis of the neurological disease modeling field using patient-derived iPSCs. The movie also includes a brief “how-to-walkthrough” of iPhemap, the online database where we deposited all curated iPSC disease phenotypes and will continually update as a tool for the research community.
